# Copolymerization of single-cell nucleic acids into balls of acrylamide gel

**DOI:** 10.1101/gr.253047.119

**Published:** 2020-01

**Authors:** Siran Li, Jude Kendall, Sarah Park, Zihua Wang, Joan Alexander, Andrea Moffitt, Nissim Ranade, Cassidy Danyko, Bruno Gegenhuber, Stephan Fischer, Brian D. Robinson, Herbert Lepor, Jessica Tollkuhn, Jesse Gillis, Eric Brouzes, Alex Krasnitz, Dan Levy, Michael Wigler

**Affiliations:** 1 Cold Spring Harbor Laboratory, Cold Spring Harbor, New York 11724, USA;; 2 Department of Pathology and Laboratory Medicine, Weill Medical College of Cornell University, New York, New York 10021, USA;; 3 Department of Urology, New York University Langone Medical Center, New York, New York 10017, USA;; 4 Department of Biomedical Engineering, Stony Brook University, Stony Brook, New York 11794, USA

## Abstract

We show the use of 5′-Acrydite oligonucleotides to copolymerize single-cell DNA or RNA into balls of acrylamide gel (BAGs). Combining this step with split-and-pool techniques for creating barcodes yields a method with advantages in cost and scalability, depth of coverage, ease of operation, minimal cross-contamination, and efficient use of samples. We perform DNA copy number profiling on mixtures of cell lines, nuclei from frozen prostate tumors, and biopsy washes. As applied to RNA, the method has high capture efficiency of transcripts and sufficient consistency to clearly distinguish the expression patterns of cell lines and individual nuclei from neurons dissected from the mouse brain. By using varietal tags (UMIs) to achieve sequence error correction, we show extremely low levels of cross-contamination by tracking source-specific SNVs. The method is readily modifiable, and we will discuss its adaptability and diverse applications.

Single-cell analyses are increasingly used for understanding the patterns of gene expression and genomic variation in complex populations of cells and tissues (Navin et al. 2011; Patel et al. 2014; Tirosh et al. 2016; Villani et al. 2017). Many droplet-based technologies have emerged as high-throughput ways to study DNA (Lan et al. 2017; Pellegrino et al. 2018) or RNA (Klein et al. 2015; Macosko et al. 2015) of single cells. However, these methods often lack the breadth of coverage (see Supplemental Text; Ding et al. 2019). Droplet merging and breakage give rise to cross-contamination. Moreover, some droplet-based methods suffer from inefficient use of samples. Therefore they are not the ideal choice for analyzing rare and valuable samples, such as cells from biopsy washes or cells microdissected from tissue samples. To address these and other needs, we developed and describe here a method that has advantages in coverage, quantitation, the efficient use of samples, sequence accuracy, and flexibility without sacrificing scalability. The set-up requires only inexpensive standard equipment and reagents, and the cost of preparing single-cell libraries is negligible compared with sequencing.

The central concept in this protocol has broad applicability. The underlying principle is the encapsulation of single cells or single nuclei in aqueous droplets containing acrylamide monomer in an oil emulsion, followed by conversion of each droplet into a ball of acrylamide gel (BAG) by polymerization. Primers containing 5′-Acrydite copolymerize with the acrylamide. Through annealing and extension, the information content of the cell is captured as nucleic acids covalently bound to the polyacrylamide matrix. After removing the oil, each BAG serves as an independent reaction vessel, accessible by diffusion in an aqueous environment to polymerases and other reagents. BAGs are then individually barcoded by split-and-pool methods, first used during the production of peptide libraries (Fodor et al. 1991), then used as a method to encode beads (Ohlmeyer et al. 1993), and finally for single-cell analysis (Cusanovich et al. 2015; Rosenberg et al. 2018). Our method has great flexibility. By varying designs of primers, enzymes, and conditions, the BAGs can be used as sources for libraries for single-cell DNA or RNA, or possibly even proteins. In this report, we show and characterize the applications for single-cell DNA copy number and RNA profiling from simple and complex mixed populations.

## Results

### Converting single cells into BAG libraries

**Figure 1.**
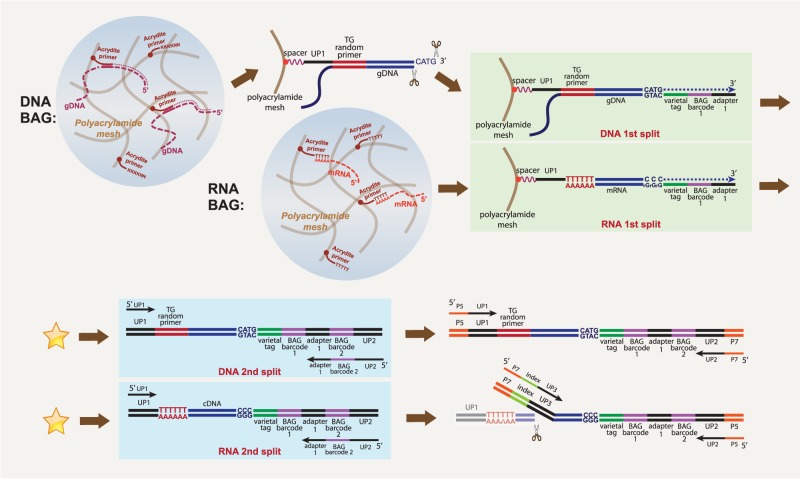
Schematic of single-cell DNA or RNA BAG-seq workflow. The star between first split and second split indicating the place where more cycles of split-and-pool can be added.

Figure 1 illustrates our protocol, which we outline here. First, we create a suspended aqueous droplet in oil containing single-cell contents and reagents and then convert that droplet into a polyacrylamide bead. By using 5′-Acrydite primers, some of the contents of single cells become linked to the bead matrix. To achieve this, we use a single-cell DroNc device (Habib et al. 2017), with one stream (aqueous phase 1) carrying the single cells or nuclei and another stream (aqueous phase 2) carrying reagents, combining both as an aqueous droplet in oil. Aqueous phase 2 contains acrylamide monomers, bis-acrylamide cross-linker, ammonium persulfate, 5′-Acrydite capture primers, and detergents in a buffer. For single-cell DNA analysis, we also include Proteinase K in aqueous phase 2. For RNA analysis, we include RNase inhibitor and omit Proteinase K. The oil phase contains TEMED, an accelerator of polymerization. During incubation in oil, the aqueous droplet forms a gel ball with the Acrydite primer covalently incorporated into the matrix.

For DNA, we use 5′-Acrydite primers made of essentially random Ts and Gs. We tested other primers, including *Alu* repeat sequence and random N primers, but these T and G primers performed best (Supplemental Fig. S1). We melt and cool to allow annealing to the 5′-Acrydite primers. For RNA, the Acrydite primers are poly(T) (see Methods; Fig. 1). We remove the oil, and the BAGs are subsequently processed in the aqueous phase. Annealing to primers is essential, as without linkage to the matrix, all nucleic acids leak out of the bead.

After removal from the oil, each BAG functions as a reaction vessel, with the nucleic acid tethered to the bead matrix. Once in the aqueous phase, we extend the primers on the captured templates, and thereby link copies of templates to the bead matrix. In subsequent steps, including split-and-pool, we add varietal tags (unique molecular identifiers) (Kivioja et al. 2012; Hicks et al. 2016) and BAG barcodes, but the method details differ if the initial template is DNA or RNA.

If the template is DNA, we make the second strand in the pool stage and cleave with a restriction endonuclease to make an adaptable end. In the first split, we use the cleavage site to add a varietal tag and the first split barcode. We pool the BAGs and redistribute them into wells for the second split, during which the second split barcode is added by PCR. We pool the PCR product from the second split and amplify using modified Illumina sequencing adapters to make the final sequencing library (for details, see Supplemental Method S1).

If the template is RNA, copying takes place in the first split yielding a DNA–RNA hybrid, and by using a “template switch oligo,” the DNA strand acquires a varietal tag and first-split barcode. The second split can be performed as with DNA (see Methods), or additional cycles of split can be performed through a denaturation–hybridization–extension procedure and the final split is performed by PCR (see Supplemental Method S2). After pooling the PCR product, tagmentation followed by PCR is used to make the final sequencing library.

For DNA or RNA, sequencing libraries are prepared from pooled BAGs. The combination of first and second split barcodes gives almost all BAGs a unique bead barcode. We partition all the reads by this barcode. We then tally the captured templates with a given barcode by counting the nearly unique combination of varietal tag and captured sequence.

Not all detected barcodes derive from BAGs with single-cell contents. To determine which barcodes correspond to BAGs with cells, we only use barcodes with high read count. To do this, we plot a cumulative sum of the barcode counts, ordered by their magnitude. We typically observe a sharp inflection point, as illustrated in Supplemental Figure S2, and use the barcodes to the left of the inflection point.

In general, we observe efficient use of input cells (see Methods). Upon loading into the microfluidic device anywhere from 0.3 to 1 mL, we can recover up to 85% of cells in the final library. Some of the input fluid is retained by the device, so yields are higher for larger input volume.

### Copy number profiles from mixed populations

**Figure 2.**
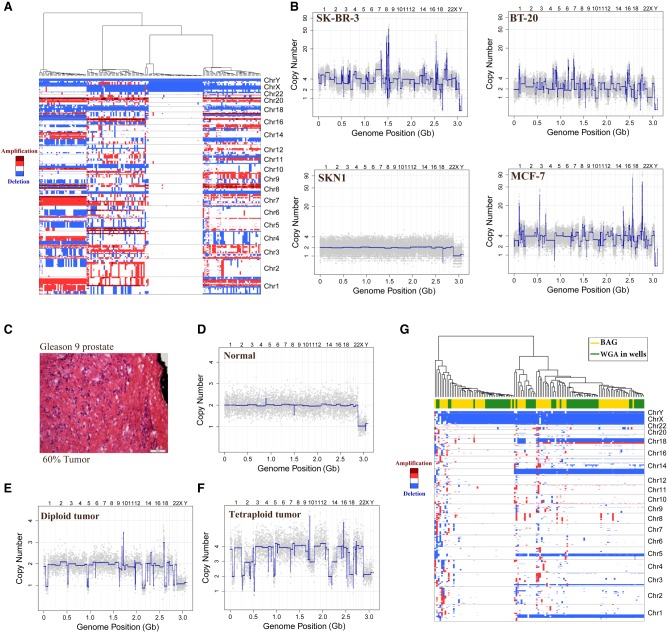
Copy number variation analysis of single-nucleus DNA (snDNA) BAG in cell lines and frozen prostate tumor. (*A*) Hierarchical clustering of four cell types SKN1, SK-BR-3, MCF-7, and BT-20 at a resolution of 20,000 bins (150 kbp per bin). Red indicates amplification, whereas blue indicates deletion. (*B*) The 20,000-bin copy number profiles from each of the four clusters in *A*. (*C*) Pathology image showing the region of Gleason 9 prostate cancer, which was estimated by pathologist as 60% tumor. Scale bar, 100 µm. (*D*–*F*) Representative snDNA BAG copy number profiles from this region: (*D*) a representative normal copy number profile; (*E*) a representative diploid tumor profile; and (*F*) a representative tetraploid tumor profile. (*G*) Hierarchical clustering of this region by combining data from both the BAG method and 96-well WGA method.

As a demonstration of our method, we applied it to mixtures of four cell sources: three breast tumor cell lines (SK-BR-3, MCF-7, and BT-20) and one normal cell strain (SKN1). We computed the copy number profiles of each BAG using empirically normalized bins (see Methods). We display the results in Figure 2, A and B, at a resolution 20,000 bins or ∼150 kbp per bin. After hierarchical clustering, we observe four clusters (Fig. 2A). One representative of each cluster is displayed as a conventional copy number profile in Figure 2B. Supplemental Figure S3 shows profiles from two BAGs with the SK-BR-3 pattern, illustrating the consistency of the method.

The method works on nuclei isolated from frozen tumor biopsies, and we illustrate this using previously published clinical material (Alexander et al. 2018). Figure 2C shows the pathology image of a region from a prostate with a Gleason 9 lesion, assessed by pathologists as 60% cancer. The BAG profiles from that region are displayed in a hierarchical cluster (Supplemental Fig. S4A). There are two clusters, one with 26 “normal” profiles and one with 39 tumor profiles. Figure 2, D through F, shows the representative copy number profiles from each clone of this region using BAG technology at a resolution of 5000 bins (Supplemental Data S1, S2). Supplemental Figure S4B compares three BAG profiles with three single-nucleus profiles obtained from an earlier published whole genome amplification (WGA) method in 96-well plates. We combined profiles from our current (BAG) and previous (WGA) methods and display the hierarchical clustering in Figure 2G. The individual profiles obtained by the two methods are largely indistinguishable.

**Figure 3.**
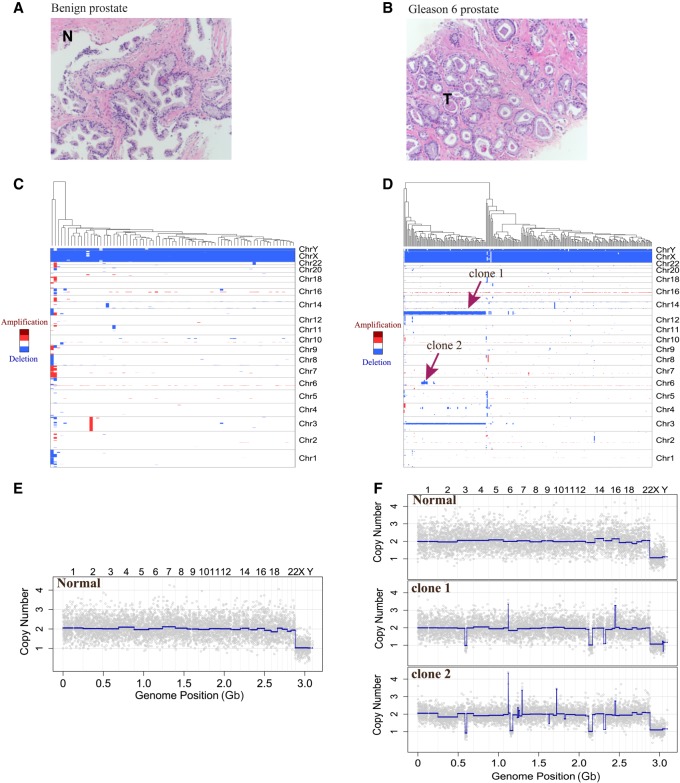
CNV study of prostate tumor biopsy wash samples from a benign region and a Gleason 6 cancer region. (*A*) A 20× magnification pathology image of a benign region of the prostate. (*B*) Pathology image of a Gleason 6 cancer region from the same patient at the same resolution. (*C*) Hierarchical clustering of biopsy wash sample from the benign region. (*D*) Hierarchical clustering of biopsy wash sample from the Gleason 6 region showing a normal clone and two tumor clones based on CNV patterns. Red arrows indicate the major (clone 1) and minor (clone 2) tumor clones. (*E*) A representative normal single-nucleus copy number profile from the biopsy wash of this benign region. (*F*) Representative single-nucleus copy number profiles from one normal clone and two tumor clones from the biopsy wash of the Gleason 6 cancer region.

To illustrate that the method works with small amounts of precious sample, we also studied two biopsy wash samples from one patient. One sample was defined by the pathologist as benign (Fig. 3A; Supplemental Data S3, S4), and the other region was Gleason 6 cancer (Fig. 3B; Supplemental Data S5, S6). We examined 75 nuclei from the biopsy wash of this benign region, all normal profiles, and 269 nuclei from the Gleason 6 region. Hierarchical clustering trees from these two biopsy wash samples are shown in Figure 3, C and D. From the Gleason 6 region, we detected one major tumor clone as 35% of the cells, and among them possibly a minor clone (seven nuclei) that possesses all the features of the major clone but also has additional unique features (Fig. 3D). Representative copy number profiles of these two samples are shown in Figure 3, E and F.

### Statistics of coverage

To measure the coverage of the method, we used SKN1, a diploid cell strain prepared from a healthy donor. The BAGs had 96 × 96 possible barcodes. We obtained 342 million read pairs, of which 84% were mapped to 88 BAGs with the largest counts (Supplemental Fig. S2A). From these 88, we characterized each BAG with respect to the numbers of read pairs, mapped reads pairs, uniquely tagged templates, and reads per template. We also characterize the total genome coverage per BAG. The data are found in Supplemental Table S1.

We sequenced about 300 million read pairs from the library made from 88 cells. We determined that 53% of paired-end reads could be properly mapped to the genome, meaning that both ends mapped within 2 kb apart had the proper orientation to each other, and Read 2 had the expected restriction endonuclease site. Because each template is captured as a single strand, with the NlaIII cleavage site marking the 3′ end, we could determine that 38.8% of the genome is captured from the plus strand, 38.9% captured from the minus strand, and 15.7% from both strands. In total, uniquely mapped reads cover a total of 60% of the human genome, with 40% of the genome mapping to at least two BAGs (Supplemental Fig. S5A).

We obtained a median number of 1.3 million reads per BAG, and a median absolute deviation (MAD) of 0.5 million. We have a multiplicity of about 3.5 reads per uniquely tagged template, yielding on average 492,490 unique templates. The median BAG barcode covered ∼1.6% of the genome. These statistics are a function of read depth, so we sampled from 10% to 100% of reads and recomputed them. Supplemental Figure S5A shows total genome coverage, as well as the proportion of the genome seen in at least two BAGs as a function of reads sampled. Supplemental Figure S5B shows the shape of coverage for each of the 88 BAGs on downsampling, normalized to total counts at 100%. The 88 curves are very similar, as shown by the small error bars at each downsampling position, and indicate the limiting return of additional sequence.

### Sequence error correction

Single-cell genome sequencing has been used for variant analysis (Wang et al. 2012; Xu et al. 2012; Zong et al. 2012). To use our method in this fashion requires an understanding of its sequence error rate. To measure error, we examined the single-cell sequence data from SKN1 for differences to the donor genome obtained from his blood DNA. We restricted analysis to those regions of the genome where the donor was well covered and homozygous to the human reference, and we sought variant sequence in the individual reads of the BAG libraries that were not reference bases. We then determined if the variant sequence was seen in multiple reads from the same template and in more than one BAG. We also examined the trinucleotide sequence context and the variant base.

**Figure 4.**
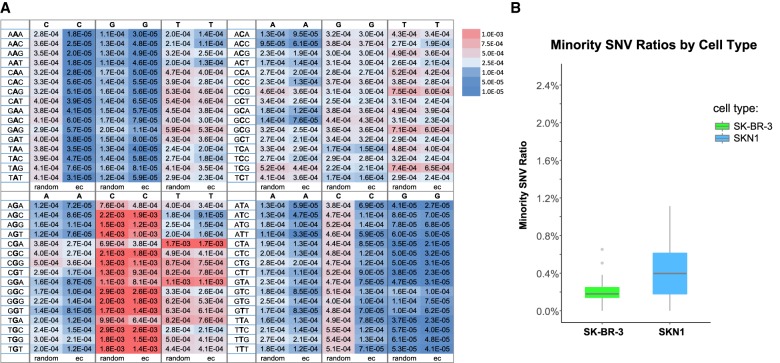
Sequence error correction (ec) and analysis of cross-contamination using error-corrected SNVs. (*A*) Comparison of error rates between random sampling and ec in trinucleotide context. The number in each box indicates the error rate and is colored by its intensity. The middle base in the trinucleotide context is the “source” base, and the single base on *top* of each column is the “destination” base. For each “destination” base, the first column corresponds to random sampling method, and the second column corresponds to the ec method. (*B*) Minority SNV ratios of SK-BR-3 nuclei and SKN1 nuclei from the four-nuclei mixing experiment using the ec method showing very low contaminations between BAGs.

Figure 4A summarizes error rates in single and multiple reads per uniquely tagged template. There are 64 trinucleotide contexts with three possible variants for the central nucleotide. Without error correction, some nucleotide contexts have low error (A or T to G, below 10^−4^) and others high (G to C, about 10^−3^). Using only multiple reads for a template, and then only when they are concordant, reduces some error rates on the order of 10-fold (e.g., A or T to C) (Fig. 4A). Some nucleotide contexts are not corrected by multiple reads in consensus (e.g., G to C, and C to G). We assume that if an error is not corrected by the concordance method, the error is due largely to initial template damage, for example, from depurination or deamination. Moreover, we observed that the error rate is lowered by reducing polymerization time or decreasing polymerization temperature. We infer this low-frequency damage may be induced by heat or the chemicals needed for polymerization, in particular the presence of persulfate, as has been previously noted (Wang et al. 2017).

One can further lower background errors to a large extent by demanding to see a variant as concordant in at least two BAGs. For applications such as studying cell lineage based on variants, one would require mutations shared by at least two cells. In Supplemental Table S2, we note the incidence of variant reads occurring at all homozygous reference positions on all chromosomes, the positions of which are seen in at least two BAGs and in each BAG with concordance. We note that if we restrict to variants appearing in at least two BAGs, then such variants occur with a frequency of 10^−5^ to 10^−6^.

Let us call a variant that appears in two BAGs, in each with concordance, a “candidate” variant. We have thus put an impressive upper bound on the error rate for candidate variants. But this is not a least upper bound, because some of these variants might actually be somatic variation between these fibroblast cells and the blood DNA, and not sequence error. To explore this further, we consider the two genome strands since we can tell them apart. In this data set, if a position is captured on one strand in at least two BAGs (in each with concordance), ∼40% of the time that position is captured on the opposite strand in at least one BAG. When the position of a candidate variant can be observed on the complementary captured strand, the complement of the variant is seen ∼56% of the time. Thus, the candidate is most likely a somatic variant between the fibroblast and blood DNA, not sequencing error. Thus, one may make discovery of somatic mutation from single-cell genome data obtained using our current protocol.

### Measuring cross-contamination

BAGs are semiclosed systems. They are porous and accessible by diffusion, but the trapped contents of the cell, once covalently linked to the polyacrylamide skeleton, will not leak out. Were this otherwise, we would not obtain the distinct copy number profiles that we see in mixed cell populations. However, we can now make this a quantitatively precise conclusion. To examine just how little cross-contamination does occur, we used SNV analysis from the four genome mixing experiments (illustrated in Fig. 2A), but looking only at the two genomes for which we had complete genome sequence data, SK-BR-3 and SKN1. We considered only “mutually distinct” variants and used the error-correction rules just described. Then we looked for consensus reads of distinct variants found in one or more BAGs that match the wrong genome (Supplemental Table S3). It is evident that there are very few distinct variants from the SK-BR-3 genome that are seen as consensus reads in SKN1 BAGs (110 out of 29,360 observed variant positions), and the reverse (261 from SKN1 out of 125,468 seen in SK-BR-3 BAGs). The average minority SNV ratio is 0.2% for SK-BR-3 nuclei and 0.4% for SKN1 nuclei (Fig. 4B), and the minority SNV ratios (0.2% to 0.4%) agree with the ploidy of SKN1 (diploid) versus SK-BR-3 (tetraploid).

### Single-cell mRNA BAG libraries

BAG technology is flexible and can also be used for single-cell mRNA analysis using a 5′-Acrydite primer containing a 5′ PCR primer 1 (UP1) with a poly(T) tail (see Fig. 1). After capture and pooling, we split BAGs into a 96-well plate and used a reverse transcriptase with terminal deoxynucleotidyl transferase (TdT) function to add to the cDNA a varietal tag, first BAG barcode, and a common adapter sequence. We pooled BAGs again and split into another 96-well plate, where the second round of BAG barcodes with Primer 2 (UP2) was hybridized to molecules with the common adapter, and amplified by PCR (see Methods; Fig. 1; for details, Supplemental Methods). Following amplification, we fragmented the cDNA amplification product using the Nextera XT kit. After final amplification using the Nextera primer and P5-UP2 primer, “Read 1” corresponds to the 5′ end of the captured nucleic acid, the varietal tag, and the BAG barcodes and is used for deconvolution and counting of templates. “Read 2” is used for mapping to the genome.

**Figure 5.**
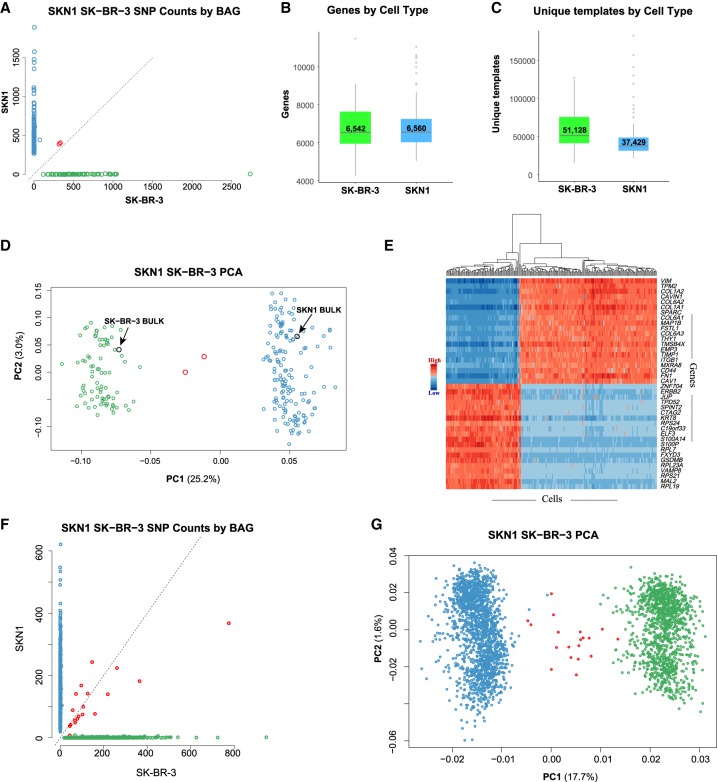
Single-cell RNA (scRNA) BAG showing high yield, low contamination, and consistent expression profiles. (*A*–*E*) A two-cycle split-pool experiment including 235 cells. (*F*,*G*) A three-cycle split-pool experiment including 2875 cells. (*A*) Scatter plot showing the number of SKN1-specific and SK-BR-3–specific SNVs found in exons for each cell. BAGs with majority SKN1 or SK-BR-3 SNVs are colored blue or green. Two (0.85% of total) BAGs without clear majority SNVs are labeled as red. (*B*) Boxplot showing the number of genes detected per cell. (*C*) Boxplot showing the number of unique templates captured per cell. (*D*) Scatter plot of PC1 versus PC2. The scRNA BAGs are colored by their majority SNVs defined in *A*. Two bulk RNA data sets for SKN1 and SK-BR-3 clusters with their respective single-cell data. The contribution of PC1 is more than eight times that of PC2 (25.2%/3.0%). (*E*) Heatmap based on 40 (20+, 20−) genes with the most positive and negative correlations to PC1. (*F*) Scatter plot showing the number of SKN1-specific and SK-BR-3–specific SNVs found in exons for each cell in the three-cycle split-pool experiments. Nineteen (0.66% of total) cells without clear majority SNVs are labeled as red. (*G*) PC1 versus PC2 from the 2875 cells in the three-cycle split-pool experiment illustrated in *F*.

To show the method works for RNA, we executed an assay on mixed cell populations of SKN1 and SK-BR-3 cells. From Read 2, we found that 70% of the bases were mapped to the exons (40% coding and 30% UTR), 17% were mapped to the introns, and 13% were mapped to the intergenic regions. These figures are similar to those we obtain using bulk RNA sequencing data. We used the cell-specific SNVs found in exons from these two cell sources to identify the captured cell, shown in Figure 5A. From 235 cells (Supplemental Fig. S2B), 233 (>99%) had a clear major source for its SNVs. We observe little to no cross-contamination judged by the SNV analysis. From these 233 BAGs, the median minority SNV ratio is 0.5% whether the minority source is SK-BR-3 or SKN1.

Two of the BAG barcodes appear to be associated with two cells. We expect this is the result of “barcode collision.” The split-and-pool method does not guarantee that each BAG receives a unique barcode. The collision rate is driven by the number of BAGs and the number of possible barcodes. If there were 96 × 96 ∼10^4^ possible barcodes and if we picked barcodes at random 235 times, we expect the 2.9 barcodes to be picked twice, producing a collision. Collisions can be reduced by increasing the number of possible barcodes, as we will discuss.

The reads were then collapsed by their varietal tags to count how many uniquely tagged templates and genes were captured in each BAG. The median number of genes captured from 152 single SKN1 cells and 81 single SK-BR-3 cells was 6560 and 6542, respectively (Fig. 5B), and the median number of uniquely tagged templates captured from single SKN1 cells and SK-BR-3 cells was 37,429 and 51,128, respectively (Fig. 5C). These numbers compare favorably to what we obtained from previously used or commercially available methods.

The average number of reads per uniquely tagged template (RPT) was 5.9 for SK-BR-3 and 6.1 for SKN1. To estimate whether we have sequenced to saturation, we downsampled the reads and recomputed the unique templates and genes detected from these reads. From the shape of the downsampling curves (see Supplemental Fig. S6), more new templates would be observed by deeper sequencing of the libraries.

To study the consistency of gene expression between single cells of the same type, we performed PCA analysis after normalizing and centering the expression matrix. The first principal component (PC1) dominates and clearly separates SKN1 cells from SK-BR-3 cells (Fig. 5D). Two SKN1 and SK-BR-3 bulk RNA expression profiles from the conventional RNA sequencing method fit well among the single-cell RNA (scRNA) expression profiles (Fig. 5D). We calculated the correlation coefficients between gene expression and PC1 and plotted a heatmap composed of the top 20 positively correlated genes and the top 20 negatively correlated genes (Fig. 5E). Among the genes most correlated with PC1 in the fibroblast cells are collagen genes, and in the epithelial cancer cell line, SK-BR-3, are the keratin 8 (*KRT8*), *ERBB2*, and *ERBB2* signaling pathway genes.

To show scalability, we implemented three cycles of split-and-pool by adding a denaturation–hybridization–extension step after the first split-and-pool (see Supplemental Method S2). This generates around 1 million (96 × 96 × 96) different BAG barcodes. We showed performance in a mixture of SKN1 and SK-BR-3 cells surveying a total of approximately 3000 cells (Supplemental Fig. S2C). After counting cell line–specific SNPs and removing BAGs with fewer than 5000 unique tags, we identified 1663 BAGs as SKN1 cells and 1193 BAGs as SK-BR-3 cells. We identified 19 BAGs as having mixed identity, showing SNP ratios between 15% and 85% (Fig. 5F). The observed barcode collision rate is 0.66%. The SKN1 and SK-BR-3 populations are easily separable in the first component of PCA analysis (Fig. 5G).

**Figure 6.**
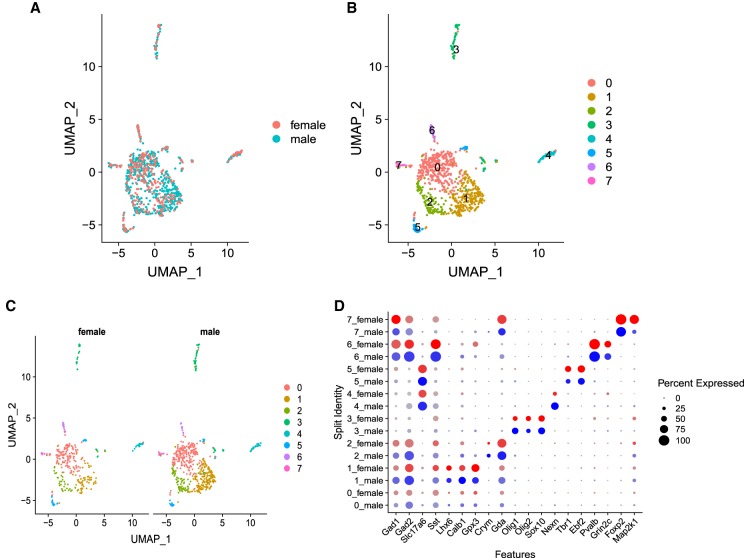
Comparison of single-nuclei RNA clusters distinguishing sexes. (*A*) UMAP clustering of 860 nuclei from brain BNSTp region, and colored by sex. (*B*) Eight clusters in *A* are distinguished and labeled using different colors. (*C*) Nuclei are split by sex. There are 540 nuclei from males and 320 nuclei from females. (*D*) Dotplot showing features expression across all clusters. The size of the dot indicates the percentage of cells within a cluster, and the brightness of color indicates the expression level in a cluster.

One key advantage of BAG-seq is its high cell-capture efficiency, making it an ideal technique for studying a rare cell population. To show this important feature and also to show its performance on complex cell populations, we performed single-nuclei RNA BAG-seq on a specific region of mouse brain. In this experiment, we collected a subset of the bed nucleus of the stria terminalis (BNST) called the principal nucleus (BNSTp) from five male mice and five female mice and pooled the nuclei from male or female mice, respectively. The area is sexually dimorphic in both mice and humans, with ∼45% more neurons present in the BNSTp of male mice compared with females (Allen and Gorski 1990; Forger et al. 2004; Sokolowski and Corbin 2012; Welch et al. 2019). We studied 540 male mouse nuclei and 320 female mouse nuclei in this experiment. After clustering, we obtained eight cell populations (Fig. 6A–C). By comparing the number of nuclei between sexes in each cluster, we identified that in one of the eight clusters, the number of nuclei from males is significantly larger than that from females (Supplemental Table S4). We identified several marker genes that distinguish between each cluster and showed that this method distinguishes between neuronal and nonneuronal cells and between excitatory and inhibitory neurons (Fig. 6D).

## Discussion

We have continued the evolution of methods for single-cell analysis. The first methods required isolating single cells into microwells (Navin et al. 2011). This idea was then extended by robotics (Wang et al. 2012; Gao et al. 2017; Gierahn et al. 2017). The method also evolved by using droplets in oil to isolate cells and then transferring the cell contents to beads coencapsulated with the cells (Klein et al. 2015; Macosko et al. 2015). Other methods used nuclei or fixed cells as vehicles for combinatorial indexing (Cusanovich et al. 2015; Rosenberg et al. 2018). Each of these methods has drawbacks, in the form of either expensive machinery, low yield per cell, limitations of Poisson sampling, requirements of nuclei isolation or cell fixation, or costly barcoded beads and reagents. In our present embodiment, we use aqueous droplets to isolate the cells, but the cell and its contents are polymerized and form the bead itself. By including Acrydite-modified primers, the nucleic acid sequences of the cell become bound to the acrylamide matrix. We call these cells in a ball of acrylamide gel (C-BAGs). Unlike other “cell bead” ideas (Tamminen and Virta 2015; Andor et al. 2018), in C-BAGs the first copy of nuclei acids is covalently bound to the gel matrix and individually tagged by a split-and-pool strategy.

The apparatus is inexpensive, and we estimate the cost per cell to be $0.50 when performed on the scale of a few hundred cells, with costs asymptotic to zero on a larger scale, which is a general feature for split-and-pool technologies. Unlike other droplet methods in which the barcoded bead is much smaller than the size of a droplet, in our method, the capture bead becomes the size of the droplet so, in theory, capture should be more efficient. The nucleic acid capture reagents that fix the cell contents to the BAG skeleton can be custom designed. The BAG that forms is permeable, and its contents can then be processed easily and cheaply in an aqueous environment. We exploit this feature to generate BAG barcodes, subsequent to their formation, by split-and-pool synthesis, obviating the need for expensive reagents or kits. We observe virtually no cross-contamination between BAGs. We do have occasional barcode collisions, as expected. These can be minimized by increasing the number of barcodes as we showed in the Results.

We have illustrated the BAG-seq method for single-cell DNA. For this purpose, we used a non-self-annealing TG primer to trap the cell DNA. The sequence distribution is sufficiently uniform (Supplemental Fig. S1B), and we use varietal tags for accurate counting of the initial templates, so that with empirical bin normalization we obtain a genome copy number resolution equivalent to our best previous manual methods. There is virtually no cross-contamination between BAGs. Sequence error can be reduced with template varietal tags and the template concordance method so that single-cell variant calling is feasible.

With minor modifications of the capture primer and the barcoding method, the C-BAGs capture RNA and can produce scRNA libraries. At our sequence depth, about 250 cells per lane of Illumina NextSeq for the two-cycle SKN1-SB-BR-3 mixing BAG-seq experiment, we capture about 50,000 unique templates per cell and on the order of 6500 expressed genes per cell. Deeper sequencing would yield higher numbers (Supplemental Fig. S6). By integrating over all BAGs, we obtain the full spectrum of genes found by bulk sequencing. We observe little to no cross-contamination, as judged by SNV analysis. For comparison to other scRNA technologies, we performed a scRNA BAG-seq using HEK293 cells (Supplemental Fig. S7). We show that the BAG-seq method exceeds the number of genes and unique templates captured per cell and has fewer barcode collisions compared with other high-throughput methods (Supplemental Text). Many other variations in methods for RNA trapping, extension, and barcoding might be tested in the future such as encapsulating beads in BAGs. The present method should keep pace with or exceed future developments in commercially available methods and at lower costs.

The protocol makes efficient use of sample cells since it does not depend on two simultaneous Poisson events: one sampling cells; the other, beads. Thus, we can examine a few hundred cells, which is important if the sample is scarce and precious. By the same token, running devices in parallel could generate millions of C-BAGs and, after split-and-pool tagging, still achieve a one-to-one correspondence between tag and cell.

As this last example illustrates, BAG technology is very flexible. Although we have presented two applications, many other possible uses of BAGs merit development. For some of these, alternative ways of linking the cell nucleic acids, or proteins, to the gel matrix will need to be developed. In principle, BAGs could capture both DNA and RNA for making dual libraries from single cells. Capturing RNA and/or protein, followed by reaction with fluorescent probes, could enable enrichment for BAGs containing the content of rare subtypes of cells, for example, by fluorescence-activated cell sorting (FACS). In principle, the BAGs are reusable, and one can select BAGs for deeper analysis as desired. It should be possible to freeze and store them until one is ready for sequencing. We might be able to reencapsulate BAGs into oil if further biochemical steps required reisolation.

## Methods

### Samples

In the experiments, we used a normal skin fibroblast cell strain (SKN1) and three breast tumor cell lines (SK-BR-3, BT-20, and MCF-7). Clinical specimens were as previously cited (Alexander et al. 2018). In particular, a tissue biopsy was obtained from a patient (COR003.GS9.2) undergoing radical prostatectomy (RP) at New York–Presbyterian/Weill Cornell Medical Center. The Gleason score (GS) at RP was GS9 (4 + 5). One-millimeter-diameter cores of frozen tissue were placed into a sterile tube and maintained on dry ice to transfer to Cold Spring Harbor Laboratory (CSHL) for further processing. Clinical and pathological data were collected and maintained in a database curated by the Weill Cornell Medical College Center's Prostate Cancer Biobank. Also, tissue biopsies were obtained from a patient (NYU005) undergoing prostate biopsy at the Smilow Comprehensive Prostate Cancer Center (SCPCC) at NYU Langone Medical Center. This patient underwent a systematic transrectal ultrasound prostate biopsy (TRUS-Bx) and an MRI-TRUS fusion-targeted biopsy (TBx). Individual cores of prostate tissue were placed in site-separated vials filled with 5 mL of sterile wash buffer (1× PBS containing 0.5% BSA [Thermo Fisher Scientific B14] and 2 mM EDTA) and gently inverted several times for 60 sec to enhance exfoliation of prostate cells. After inversion, prostate cores were removed from the wash solution using disposable single-use sterile forceps and transferred to site-separated containers with formalin fixative for histological processing and pathological evaluation. The biopsy GS for NYU005 was GS7 (3 + 4). The presence of perineural invasion (PNI) was noted in the final diagnostic pathology report. Prostate biopsy washings were kept on wet ice for 1–2 h during transfer to CSHL, where the cell suspensions were briefly centrifuged to pellet the cells and lysed using NST-DAPI buffer as described in the previous study (Navin et al. 2011). This patient underwent a RP in which the GS7 at biopsy was downgraded to GS6 at RP.

Mouse experiments were performed under the guidelines of the CSHL Institutional Animal Care and Use Committee (IACUC). Esr1cre (Lee et al. 2014) and ROSA26CAG-Sun1/sfGFP (INTACT) (Mo et al. 2015) mice were obtained from the Jackson Laboratory and crossed to generate Esr1cre; INTACT animals.

### Isolating nuclei

Nuclei were prepared from four sources. First, nuclei from cell lines were isolated using nuclei EZ Lysis buffer from a nuclei EZ prep kit (Sigma-Aldrich NUC101). Second, nuclei from frozen tissue were isolated using the protocol described by Habib et al. (2017). The nuclei were stored in ice-cold PBS-BSA (0.05%) buffer. Third, nuclei from biopsy washings were isolated using NST-DAPI buffer. Nuclei from prostate biopsy washes were prepared by gently centrifuging washings at 1000 rpm for 5 min to pellet the exfoliated cells followed by removal of supernatant and addition of 1.0 mL of NST-DAPI buffer to the cell pellet. All nuclei suspensions were filtered through a 35-µm cell strainer before flow sorting. Single nuclei regardless of ploidy were sorted into an Eppendorf tube using a BD Biosystems SORP flow cytometer. Fourth, nuclei were isolated from the mouse BNSTp as described previously (Mo et al. 2015) with minor modifications. BNSTp tissue was microdissected from 500-mm brain sections after rapid decapitation of anesthetized animals. Tissue was pooled from five P14 male and female animals heterozygous for the Esr1cre and INTACT alleles. The tissue was dounce homogenized 15× with a loose pestle in a glass homogenizer containing homogenization medium (250 mM sucrose, 25 mM KCl, 5 mM MgCl_2_, 20 mM Tricine-KOH, 1 mM DTT, 0.15 mM spermine, 0.5 mM spermidine, 1× EDTA-free protease inhibitor cocktail [Roche], 0.2 U/μL RNaseOUT [Thermo Fisher Scientific], adjusted to pH 7.8). We added 0.3% IGEPAL CA-630, and the tissue was further dounced 5× with a tight pestle. The homogenate was then filtered through a 40-µm strainer and mixed 1:1 with 50% OptiPrep solution (Millipore Sigma), prepared in dilution buffer (150 mM KCl, 30 mM MgCl_2_, 120 mM Tricine-KOH, adjusted to pH 7.8). The homogenate was underlaid with 5 mL of 30% and 40% OptiPrep solution, respectively, and centrifuged at 9200 RPM for 18 min at 4°C in an ultracentrifuge using a Beckman SW-28 swinging bucket rotor. After centrifugation, ∼2 mL of nuclei solution was removed from the 30%–40% OptiPrep interface by tube puncture using a 3-mL syringe attached to an 18-gauge needle.

### Single-nucleus/cell encapsulation

Nuclei were encapsulated into aqueous droplets using a 70-µm-channel DroNc-seq device (Nanoshift), and the droplets were polymerized into BAGs. The droplet formation requires two aqueous phases and one oil phase. The oil phase recipe and acrylamide monomer concentration were adapted from the published protocol (Zilionis et al. 2017), but 5% surfactant (Ran Technologies 008-FluoroSurfactant) was used in HFE-7500 oil (Oakwood Chemical 051243). Aqueous phase 1 is nuclei or cells in PBS-BSA (0.05%) buffer.

For the single-cell DNA experiments and for the purpose of forming a BAG, 1 mL of aqueous phase 2 contains 180 µL of 40% AA/bis-acrylamide solution (Sigma-Aldrich A9926), 129 µL of 40% Acrylamide solution (Sigma-Aldrich A4058), 160 µL of 500 µM Acrydite-random (TG) primer (“/5ACryd//iSp18/TGTGTTGGGTGTGTTTGGKKKKKKKGKKKKKKKKNN,” Integrated DNA Technologies), 100 µL of 1M Tris-HCl (pH 7.5), 50 µL of 0.5M EDTA, 10 µL of 20% sarkosyl (Sigma-Aldrich L7414), 20 µL of Proteinase K (Sigma-Aldrich P4850), 20 µL of 0.1M DTT, 60 µL of freshly made 10% APS, and 271 µL of H_2_O. The output of the device was collected into 1.5-mL Eppendorf tubes preloaded with 300 µL mineral light oil. Upon collection, a milky-colored droplet layer forms between the heavy and light transparent oil layers in the tube.

The tubes were then incubated overnight at 50°C. After incubation, the bottom heavy oil layer was replaced by a new oil layer consisting of FC-40 oil (Sigma-Aldrich F9755) with 5% surfactant. The tubes were then transferred to a heating block preset at 95°C for melting DNA. After heating for 12 min, the tubes were incubated for 1 h at 55°C and then for another 10 min at room temperature to allow annealing. The top and bottom layers of oil were removed, and the BAGs were washed twice using a mixture of 600 µL 6× SSC solution (Thermo Fisher Scientific 15557036) and 150 µL of 1H,1H,2H,2H-Perfluoro-1-octanol (Sigma-Aldrich 370533). The BAGs were then washed once using 6× SSC solution and once with 1× NEBuffer 2.

For the scRNA experiments, 1 mL of aqueous phase 2 contains 180 µL of 40% AA/bis-acrylamide solution, 129 µL of 40% Acrylamide solution, 80 µL of 500 µM Acrydite-poly(T) primer (“/5ACryd//iSp18/AAGCAGTGGTATCAACGCAGAGTNNWNNNSTTTTTTTTTTTTTTTTTTTTTTTTTTTTTT,” Integrated DNA Technologies), 70 µL of 1M Tris-HCl (pH 7.5), 50 µL of 0.5M EDTA, 10 µL of 20% sarkosyl (Sigma-Aldrich L7414), 100 µL of SUPERase•In RNase inhibitor (Thermo Fisher Scientific AM2696), 100 µL of 10% NP-40 (Thermo Fisher Scientific 28324), 30 µL of 0.1M DTT, 60 µL of freshly made 10% APS, and 191 µL of H_2_O.

The tubes were incubated for 2.5 h at room temperature for polymerization and then transferred to a heat block for 5 min at 50°C. The tubes were then incubated for another 10 min at room temperature. The BAGs were collected as previously described for DNA experiments but were washed using 5× RT buffer instead of 1× NEBuffer 2 in the DNA experiment.

### DNA BAG first split-and-pool barcoding

Immediately after the BAGs were collected and washed, a linear extension step using DNA polymerase I (NEB M0210) was performed for 1.5 h at room temperature and then for 30 min at 37°C. That was followed by a 3′ exonuclease treatment using exonuclease I (NEB, M0293) to chew up unused single-stranded primers. The DNA in BAGs were cut using NlaIII (NEB R0125) to generate a 3′CATG overhang. The BAGs were then distributed into a 96-well plate. In each well, we added dNTP (Sigma-Aldrich 11814362001) and well-specific primers with the following structure: 5′ adapter 1–BAG barcode 1–varietal tag–CATG. The last two bases (T and G) are locked nucleic acid (LNA) to improve annealing to the four-base overhang. We then perform what we call a ligation-extension reaction, in which ligation and extension occur in the same reaction. To be specific, after the BAGs were first incubated with 1 µL of 100-µM well-specific primers in 1× Quick Ligation buffer (NEB M2200) in a total volume of 14 µL for 20 min at 4°C with rotation, 0.75 µL of quick ligase (NEB M2200) and 0.75 µL of Klenow fragment (NEB M0212) in 4.5 µL of 1× Quick Ligation buffer were added. The plate was incubated for another 20 min at 4°C with rotation and then for 30 min at 10°C to promote ligation. Following that, the plate was then rotated for 40 min at room temperature and then for another 40 min at 37°C to allow linear extension. The reaction was stopped by high-EDTA buffer STOP-25. The beads were then pooled together and washed by STOP-10 (Supplemental Method S1).

### RNA BAG first split-and-pool barcoding

For scRNA experiments, immediately after we collected and washed the gel balls from oil, we distributed them into 96-well plates for reverse transcription using Maxima H Minus Reverse Transcriptase (Thermo Fisher Scientific EP0751). During reverse transcription, each well has a well-specific template-switch-oligo with the structure: 5′ adapter 1–BAG barcode 1–varietal tag–rGrGrG. Here “rG” means a ribonucleotide guanine base. The BAG barcode 1 sequence and varietal tag sequence were copied to the cDNA by reverse transcriptase. The reaction was stopped by high-SDS buffer TE-SDS (Supplemental Method S2) and then pooled and washed by STOP-10 buffer. An exonuclease reaction was followed to chew up the free primers in the BAGs.

### The last split-and-pool barcoding

For both single-cell DNA and RNA experiments, the last round of BAG barcode was added in the same way. Many strategies can be used to perform split-and-pool (Cao et al. 2017; Vitak et al. 2017; Rosenberg et al. 2018; Cao et al. 2019), and additional rounds of BAG split-barcodes can be added based on the common sequence from the previous round (Supplemental Method S2). Here we used PCR to add 96 different BAG barcodes in the last split. In each well, there is a universal PCR primer and a well-specific primer containing different barcodes. BAGs from the last split-and-pool were evenly distributed into 96 wells. DNA BAGs were amplified using NEBNext ultra II Q5 master mix (NEB M0544). RNA BAGs were amplified using KAPA HiFi HotStart ReadyMix (Roche KK2602). The PCR product was pooled together and purified using AMPure XP magnetic beads (Bechman Coulter A63881).

### Single-nuclei WGA method

We used single-nuclei DNA data previously generated from a previous WGA method (Alexander et al. 2018). Briefly, single nuclei were deposited into individual wells in a 96-well plate and amplified using GenomePlex WGA4 kit (Sigma-Aldrich WGA4-50RXN) according to the manufacturer's instructions. WGA DNA was sonicated using a Covaris focus acoustics system. The Covaris E210 300 ± sonication program generated WGA DNA inserts of the desired length, ∼300 bp (range 200–400 bp), for library construction. Customized well-specific barcodes were ligated to the fragments in each well. Multiple libraries were combined into pools ranging from eight to 12 libraries to pools of 96 libraries for 76-bp single-read sequencing on single lanes of Illumina's GAIIx and HiSeq flowcells, respectively. The first 30 bases of each read were trimmed to remove any WGA primer sequence.

### Bulk RNA sequencing

Total RNA from each cell line was extracted using Direct-zol RNA MiniPrep plus kit (Zymo Research R2070). mRNA isolation was performed using a NEBNext Poly(A) mRNA magnetic isolation module (NEB E7490). Sequencing library was prepared using a NEBNext ultra II directional RNA library prep kit for Illumina (NEB E7760).

### Estimating cell capture efficiency

First, we measured the cell solution volume and cell concentration using a cell counter. We loaded a one:one ratio of cell solution (aqueous phase 1) and aqueous phase 2 into the microfluidic device. After collecting and polymerizing the droplets, we measured the volume of the recovered aqueous phase containing BAGs, as well as the total number of BAGs by counting under a stereo-microscope. At this time point, we measured the cell occupancy rate in BAGs by DAPI staining and counting through a fluorescent microscope. BAGs with a cell in them were much brighter than empty BAGs in DAPI channel. By using the cell occupancy rate, we estimated the total number of recovered cells in BAGs.

We also measured the number of BAGs by counting under a stereo-microscope before we made the cDNA library, and recorded the number of cells in the final sequencing library with high read counts. There was little loss of BAGs during the split-and-pool procedure, and essentially every BAG with a cell yielded a single-cell library.

### Read alignment

For DNA data, Illumina sequence files were preprocessed before mapping to remove reads that do not conform to expectation, to retain those that do, and to trim away sequences not needed for mapping. In particular, read pairs were removed if the CATG NlaIII cut site sequence was not in base positions 31 to 34 on Read 2 with a maximum of one mismatched base. Reads 1 and 2 were then both trimmed to remove 3′ bases that matched Illumina adapter sequence or universal primer sequence. Read pairs with both ends at least 100 bases after trimming were retained for mapping. Reads were mapped to the UCSC hg19 reference genome using HISAT2 version 2.1.0 (Kim et al. 2015) with default parameters except for the following: -3 25, -X 2000, --no-spliced-alignment. Aligning reads to genome assembly GRCh38 would not impact our study, as the updates to the genome assembly are primarily related to population variation and filling of gaps (Schneider et al. 2017). The identifying sequence in the DNA protocol is on Read 2. The BAG barcode, base positions one through six appended to base positions 22 through 26, and the varietal tag, base positions 27 through 30, were appended the read ID in the FASTQ file. This allowed read pair identity to be tracked through subsequent processing.

The first three steps in the RNA data processing pipeline are as follows: (1) select reads with valid sequence structure on Read 1 to be included in the analysis, (2) extract identifying BAG barcode and varietal tag sequences and append these to the read ID in the FASTQ files, and (3) map reads. The different RNA libraries in the paper were processed in slightly different ways.

#### Step 1 (check sequence structure)

For the SKN1, SK-BR-3 2 split-pool library, reads with GGG in positions 38 through 40 and positions seven through 25 matching the primer sequence, AGTGGAAAAGGAAGGTGGT, up to two mismatches, were included in further processing. For the HEK293 and the BNSTp libraries, reads with GGG in positions 38 through 40, allowing up to one mismatch, and positions one through six and 26 through 31 having valid BAG barcodes were included for further processing. For the SKN1, SK-BR-3 3 split-pool sample, reads with GGG in positions 67 through 69, allowing up to one mismatch, and positions one through six, 30 through 35, and 55 through 60 having valid BAG barcodes were included for further processing.

#### Step 2 (extract BAG barcode and varietal tag)

For the SKN1, SK-BR-3 2 split-pool, HEK293, and BNSTp libraries, BAG barcodes are positions one through six and 26 through 31 from Read 1. Varietal tags are positions 32 through 37 and 43 through 48 from Read 1. For the SKN1, SK-BR-3 3 split-pool, BAG barcodes are positions one through six, 30 through 35, and 55 through 60 from Read 1. Varietal tags are positions 61 through 66 and 72 through 77 from Read 1.

#### Step 3 (map to reference genome)

For the SKN1, SK-BR-3 2 split-pool, SKN1, SK-BR-3 3 split-pool, and HEK293 libraries, 76 bases of Read 2 were mapped to the UCSC hg19 reference genome with UCSC refGene annotations for known splice sites using HISAT2 version 2.1.0 with default parameters. For the BNSTp library, 50 bases of Read 2 were mapped to the UCSC mm9 reference genome with UCSC refGene annotations for known splice sites using HISAT2 version 2.1.0 with default parameters. The updates in GRCm38 (mm10) from mm9 mainly filled gaps and finished the sequence of repetitive genomic regions, so the genic sequence in mm9 is sufficiently complete for the analysis presented here.

### Copy number analysis

For the purpose of copy number analysis, the genome was divided into either 5000 or 20,000 bins. The bin boundaries were determined empirically from the data to generate a uniform distribution for the number of tags mapping to each bin assuming a constant copy number (Supplemental Fig. S1B). For this purpose, all the reads from the good SKN1 single-cell libraries were used.

Bincount data for all BAG barcodes with at least 100,000 unique tags, based on varietal tag and mapping location, were normalized by first computing log(bincount + 1)/mean(bincount + 1) and then further normalized for GC content by lowess normalization in R programming language (R Core Team 2018) with parameter *f* = 0.05. The normalized bincount vectors were then segmented using DNAcopy version 1.50.5 (Olshen et al. 2004). DNAcopy parameters used were alpha = 0.02, nperm = 1000, undo.SD = 0.5, and min.width = 3. Copy number heatmaps were made using the heatmap function in R using the segmented bin values. The distance function used was “manhattan,” and the hierarchical clustering agglomeration method used was “ward.D2.”

Individual genome plots were made after estimating ploidy. After normalization, the segmentation vectors have a mean value of one. Ploidy was estimated by multiplying these vectors by 1.5, 1.55, 1.6, …, 4.5 and using the multiplier that minimizes the sum of square error from the multiplied vector to the multiplied vector rounded to nearest integers. This multiplier is the ploidy estimate. The segmentation vector is multiplied by the ploidy estimate to get a segmentation that has as much as possible of the genome on segments close to integer values. For SKN1, the frozen tumor sample, and the biopsy washes, these segmentation values are the *y*-axis values on the genome plots. For the cell line samples in the four-nuclei mixing library, there are copy number values ranging from zero to almost 100. To visualize these values more clearly, the *y*-axis values are log(*y* + 1) with horizontal lines corresponding to copy numbers 1, 2, 3, 4, 20, 50, and 80 displayed.

### Base calling error rate analysis

To assess the sequence error in libraries made by the BAG method, we used assays based on SKN1. This is a cell strain for which we have the whole-genome sequence (WGS) of the donor from his blood DNA. Illumina sequence data from the donor were mapped to the reference genome using Bowtie 2 version 2.3.2-legacy (Langmead and Salzberg 2012) with indel realignment using GATK version 1.6-13 (McKenna et al. 2010). Mapped reads were selected for error rate analysis provided they had a read mapping quality at least 30, bases called with a base quality at least 30, genome positions with a read depth of at least 20, and no SNPs or indels called in this region. This resulted in 1.25 Gb (1.25 billion bases) used for this analysis. At these 1.25 Gb, the BAG data were evaluated for mismatches to the reference genome, as follows. We first determined “template read sets” as the set of reads sharing identical BAG barcode, varietal tags, and map position. We called positions from template read sets of at least two members and then only if at least 80% of reads agreed. Most of the nonconsensus base positions had exactly two reads, one of which did not match the reference genome.

### Cell source–specific SNVs

Cell-specific SNVs were called as follows. Illumina WGS data for the cell sources SKN1 and SK-BR-3 were mapped using Bowtie 2 version 2.3.2-legacy. Variants are called using reads with mapping quality of at least 30 where a nonreference base (with base quality at least 20) is seen at least three times and in at least 5% of reads covering this position. A variant is considered to be specific to one cell line if the variant is not seen in the other cell line where there are at least 12 reads of mapping quality at least 30 and requiring a base quality at least 20. There were 617,608 SNVs specific to SK-BR-3 and 561,443 specific to SKN1. BAG data were evaluated at these SNV sites after removing six SNV sites prone to anomalous mapping artifacts. These positions were Chr 1: 569874, Chr 6: 58777419, Chr 6: 58778584, Chr 6: 58779097, Chr 7: 61969087, and Chr 10: 42385520.

### Gene expression analysis

For bulk RNA analysis, reads that mapped completely within exons for a transcript in UCSC refGene annotations were counted and assigned to that transcript. Values at reads per kilobase per million reads (RPKM) were then computed for all transcripts. To get RPKM values for a gene with multiple transcripts, the transcript with the highest RPKM value was used.

For single-cell analysis, reads that mapped with ≥50% of the read length within exons for a transcript in UCSC refGene annotations were counted for that transcript's gene.

### RNA principal component analysis and clustering

For the SKN1, SK-BR-3 experiments, expression level values were first normalized by the mean for each sample, and then the log of the expression level +1 was centered for each gene using the center function in R with the scale parameter = F. The principal components were computed using the “prcomp” function in R with parameters center = T and scale = T. The coefficients of the first two principal components were plotted in a scatter plot with points colored according to their cell type as assessed by SNV analysis. The genes selected for the heatmap were the 40 genes with the most extreme correlations (20 most positive and 20 most negative) to the loadings on principal component 1. The data were clustered by sample and displayed using the R heatmap function with clustering parameters, distance function “Euclidean,” and hierarchical clustering agglomeration method “complete.”

For the mouse BNSTp experiment, we used R package Seurat v3 (Stuart et al. 2019) and UMAP clustering method (McInnes et al. 2018) to cluster digital expression data for the 860 nuclei. We used the default parameters for the Seurat package and used the first 30 PCA components for the UMAP function.

All aspects of the research were performed with Institutional Review Board approval.

## Data access

The raw sequencing reads from the cell lines in this study have been submitted to the NCBI Sequence Read Archive (SRA; https://www.ncbi.nlm.nih.gov/sra/) under accession number PRJNA566441.

## Supplementary Material

Supplemental Material

## References

[GR253047LIC1] Alexander J, Kendall J, McIndoo J, Rodgers L, Aboukhalil R, Levy D, Stepansky A, Sun G, Chobardjiev L, Riggs M, 2018 Utility of single-cell genomics in diagnostic evaluation of prostate cancer. Cancer Res 78: 348–358. 10.1158/0008-5472.CAN-17-1138 29180472 PMC5771881

[GR253047LIC2] Allen LS, Gorski RA. 1990 Sex difference in the bed nucleus of the stria terminalis of the human brain. Journal of Comparative Neurology 302: 697–706. 10.1002/cne.903020402 1707064

[GR253047LIC3] Andor N, Lau BT, Catalanotti C, Kumar V, Sathe A, Belhocine K, Wheeler TD, Price AD, Kang M, Stafford D. 2018 Joint single cell DNA-Seq and RNA-Seq of gastric cancer reveals subclonal signatures of genomic instability and gene expression. bioRxiv 10.1101/445932

[GR253047LIC4] Cao J, Packer JS, Ramani V, Cusanovich DA, Huynh C, Daza R, Qiu X, Lee C, Furlan SN, Steemers FJ, 2017 Comprehensive single-cell transcriptional profiling of a multicellular organism. Science 357: 661–667. 10.1126/science.aam8940 28818938 PMC5894354

[GR253047LIC5] Cao J, Spielmann M, Qiu X, Huang X, Ibrahim DM, Hill AJ, Zhang F, Mundlos S, Christiansen L, Steemers FJ, 2019 The single-cell transcriptional landscape of mammalian organogenesis. Nature 566: 496–502. 10.1038/s41586-019-0969-x 30787437 PMC6434952

[GR253047LIC6] Cusanovich DA, Daza R, Adey A, Pliner HA, Christiansen L, Gunderson KL, Steemers FJ, Trapnell C, Shendure J. 2015 Multiplex single-cell profiling of chromatin accessibility by combinatorial cellular indexing. Science 348: 910–914. 10.1126/science.aab1601 25953818 PMC4836442

[GR253047LIC7] Ding J, Adiconis X, Simmons SK, Kowalczyk MS, Hession CC, Marjanovic ND, Hughes TK, Wadsworth MH, Burks T, Nguyen LT, 2019 Systematic comparative analysis of single cell RNA-sequencing methods. bioRxiv 10.1101/632216

[GR253047LIC8] Fodor SP, Read JL, Pirrung MC, Stryer L, Lu AT, Solas D. 1991 Light-directed, spatially addressable parallel chemical synthesis. Science 251: 767–773. 10.1126/science.1990438 1990438

[GR253047LIC9] Forger NG, Rosen GJ, Waters EM, Jacob D, Simerly RB, De Vries GJ. 2004 Deletion of *Bax* eliminates sex differences in the mouse forebrain. Proc Natl Acad Sci 101: 13666–13671. 10.1073/pnas.0404644101 15342910 PMC518810

[GR253047LIC10] Gao R, Kim C, Sei E, Foukakis T, Crosetto N, Chan L-K, Srinivasan M, Zhang H, Meric-Bernstam F, Navin N. 2017 Nanogrid single-nucleus RNA sequencing reveals phenotypic diversity in breast cancer. Nat Commun 8: 228 10.1038/s41467-017-00244-w 28794488 PMC5550415

[GR253047LIC11] Gierahn TM, Wadsworth MH II, Hughes TK, Bryson BD, Butler A, Satija R, Fortune S, Love JC, Shalek AK. 2017 Seq-Well: portable, low-cost RNA sequencing of single cells at high throughput. Nat Methods 14: 395–398. 10.1038/nmeth.4179 28192419 PMC5376227

[GR253047LIC12] Habib N, Avraham-Davidi I, Basu A, Burks T, Shekhar K, Hofree M, Choudhury SR, Aguet F, Gelfand E, Ardlie K, 2017 Massively parallel single-nucleus RNA-seq with DroNc-seq. Nat Methods 14: 955–958. 10.1038/nmeth.4407 28846088 PMC5623139

[GR253047LIC13] Hicks J, Navin N, Troge J, Wang Z, Wigler M. 2016 Varietal counting of nucleic acids for obtaining genomic copy number information. U.S. patent no. US20140065609A.

[GR253047LIC14] Kim D, Langmead B, Salzberg SL. 2015 HISAT: a fast spliced aligner with low memory requirements. Nat Methods 12: 357–360. 10.1038/nmeth.3317 25751142 PMC4655817

[GR253047LIC15] Kivioja T, Vähärautio A, Karlsson K, Bonke M, Enge M, Linnarsson S, Taipale J. 2012 Counting absolute numbers of molecules using unique molecular identifiers. Nat Methods 9: 72–74. 10.1038/nmeth.1778 22101854

[GR253047LIC16] Klein AM, Mazutis L, Akartuna I, Tallapragada N, Veres A, Li V, Peshkin L, Weitz DA, Kirschner MW. 2015 Droplet barcoding for single-cell transcriptomics applied to embryonic stem cells. Cell 161: 1187–1201. 10.1016/j.cell.2015.04.044 26000487 PMC4441768

[GR253047LIC17] Lan F, Demaree B, Ahmed N, Abate AR. 2017 Single-cell genome sequencing at ultra-high-throughput with microfluidic droplet barcoding. Nat Biotechnol 35: 640–646. 10.1038/nbt.3880 28553940 PMC5531050

[GR253047LIC18] Langmead B, Salzberg SL. 2012 Fast gapped-read alignment with Bowtie 2. Nat Methods 9: 357–359. 10.1038/nmeth.1923 22388286 PMC3322381

[GR253047LIC19] Lee H, Kim D-W, Remedios R, Anthony TE, Chang A, Madisen L, Zeng H, Anderson DJ. 2014 Scalable control of mounting and attack by Esr1^+^ neurons in the ventromedial hypothalamus. Nature 509: 627–632. 10.1038/nature13169 24739975 PMC4098836

[GR253047LIC20] Macosko EZ, Basu A, Satija R, Nemesh J, Shekhar K, Goldman M, Tirosh I, Bialas AR, Kamitaki N, Martersteck EM, 2015 Highly parallel genome-wide expression profiling of individual cells using nanoliter droplets. Cell 161: 1202–1214. 10.1016/j.cell.2015.05.002 26000488 PMC4481139

[GR253047LIC21] McInnes L, Healy J, Melville J. 2018 UMAP: Uniform Manifold Approximation and Projection for dimension reduction. arXiv:1802.03426 [stat.ML].

[GR253047LIC22] McKenna A, Hanna M, Banks E, Sivachenko A, Cibulskis K, Kernytsky A, Garimella K, Altshuler D, Gabriel S, Daly M, 2010 The Genome Analysis Toolkit: a MapReduce framework for analyzing next-generation DNA sequencing data. Genome Res 20: 1297–1303. 10.1101/gr.107524.110 20644199 PMC2928508

[GR253047LIC23] Mo A, Mukamel EA, Davis FP, Luo C, Henry GL, Picard S, Urich MA, Nery JR, Sejnowski TJ, Lister R, 2015 Epigenomic signatures of neuronal diversity in the mammalian brain. Neuron 86: 1369–1384. 10.1016/j.neuron.2015.05.018 26087164 PMC4499463

[GR253047LIC24] Navin N, Kendall J, Troge J, Andrews P, Rodgers L, McIndoo J, Cook K, Stepansky A, Levy D, Esposito D, 2011 Tumour evolution inferred by single-cell sequencing. Nature 472: 90–94. 10.1038/nature09807 21399628 PMC4504184

[GR253047LIC25] Ohlmeyer M, Swanson RN, Dillard LW, Reader JC, Asouline G, Kobayashi R, Wigler M, Still WC. 1993 Complex synthetic chemical libraries indexed with molecular tags. Proc Natl Acad Sci 90: 10922–10926. 10.1073/pnas.90.23.10922 7504286 PMC47893

[GR253047LIC26] Olshen AB, Venkatraman E, Lucito R, Wigler M. 2004 Circular binary segmentation for the analysis of array-based DNA copy number data. Biostatistics 5: 557–572. 10.1093/biostatistics/kxh008 15475419

[GR253047LIC27] Patel AP, Tirosh I, Trombetta JJ, Shalek AK, Gillespie SM, Wakimoto H, Cahill DP, Nahed BV, Curry WT, Martuza RL, 2014 Single-cell RNA-seq highlights intratumoral heterogeneity in primary glioblastoma. Science 344: 1396–1401. 10.1126/science.1254257 24925914 PMC4123637

[GR253047LIC28] Pellegrino M, Sciambi A, Treusch S, Durruthy-Durruthy R, Gokhale K, Jacob J, Chen TX, Geis JA, Oldham W, Matthews J, 2018 High-throughput single-cell DNA sequencing of acute myeloid leukemia tumors with droplet microfluidics. Genome Res 28: 1345–1352. 10.1101/gr.232272.117 30087104 PMC6120635

[GR253047LIC29] R Core Team. 2018 R: a language and environment for statistical computing. R Foundation for Statistical Computing, Vienna https://www.R-project.org/.

[GR253047LIC30] Rosenberg AB, Roco CM, Muscat RA, Kuchina A, Sample P, Yao Z, Graybuck LT, Peeler DJ, Mukherjee S, Chen W, 2018 Single-cell profiling of the developing mouse brain and spinal cord with split-pool barcoding. Science 360: 176–182. 10.1126/science.aam8999 29545511 PMC7643870

[GR253047LIC31] Schneider VA, Graves-Lindsay T, Howe K, Bouk N, Chen H-C, Kitts PA, Murphy TD, Pruitt KD, Thibaud-Nissen F, Albracht D, 2017 Evaluation of GRCh38 and de novo haploid genome assemblies demonstrates the enduring quality of the reference assembly. Genome Res 27: 849–864. 10.1101/gr.213611.116 28396521 PMC5411779

[GR253047LIC32] Sokolowski K, Corbin JG. 2012 Wired for behaviors: from development to function of innate limbic system circuitry. Front Mol Neurosci 5: 55 10.3389/fnmol.2012.00055 22557946 PMC3337482

[GR253047LIC33] Stuart T, Butler A, Hoffman P, Hafemeister C, Papalexi E, Mauck WM III, Hao Y, Stoeckius M, Smibert P, Satija R. 2019 Comprehensive integration of single-cell data. Cell 177: 1888–1902.e21. 10.1016/j.cell.2019.05.031 31178118 PMC6687398

[GR253047LIC34] Tamminen MV, Virta MP. 2015 Single gene-based distinction of individual microbial genomes from a mixed population of microbial cells. Front Microbiol 6: 195 10.3389/fmicb.2015.00195 25814987 PMC4356102

[GR253047LIC35] Tirosh I, Izar B, Prakadan SM, Wadsworth MH, Treacy D, Trombetta JJ, Rotem A, Rodman C, Lian C, Murphy G, 2016 Dissecting the multicellular ecosystem of metastatic melanoma by single-cell RNA-seq. Science 352: 189–196. 10.1126/science.aad0501 27124452 PMC4944528

[GR253047LIC36] Villani A-C, Satija R, Reynolds G, Sarkizova S, Shekhar K, Fletcher J, Griesbeck M, Butler A, Zheng S, Lazo S, 2017 Single-cell RNA-seq reveals new types of human blood dendritic cells, monocytes, and progenitors. Science 356: eaah4573 10.1126/science.aah4573 28428369 PMC5775029

[GR253047LIC37] Vitak SA, Torkenczy KA, Rosenkrantz JL, Fields AJ, Christiansen L, Wong MH, Carbone L, Steemers FJ, Adey A. 2017 Sequencing thousands of single-cell genomes with combinatorial indexing. Nat Methods 14: 302–308. 10.1038/nmeth.4154 28135258 PMC5908213

[GR253047LIC38] Wang J, Fan HC, Behr B, Quake SR. 2012 Genome-wide single-cell analysis of recombination activity and de novo mutation rates in human sperm. Cell 150: 402–412. 10.1016/j.cell.2012.06.030 22817899 PMC3525523

[GR253047LIC39] Wang Y, Liu C, Hong T, Wu F, Yu S, He Z, Mao W, Zhou X. 2017 Application of ammonium persulfate for selective oxidation of guanines for nucleic acid sequencing. Molecules 22: 1222 10.3390/molecules22071222 PMC6152272 28753999

[GR253047LIC40] Welch JD, Kozareva V, Ferreira A, Vanderburg C, Martin C, Macosko EZ. 2019 Single-cell multi-omic integration compares and contrasts features of brain cell identity. Cell 177: 1873–1887.e17. 10.1016/j.cell.2019.05.006 31178122 PMC6716797

[GR253047LIC41] Xu X, Hou Y, Yin X, Bao L, Tang A, Song L, Li F, Tsang S, Wu K, Wu H, 2012 Single-cell exome sequencing reveals single-nucleotide mutation characteristics of a kidney tumor. Cell 148: 886–895. 10.1016/j.cell.2012.02.025 22385958 PMC7458411

[GR253047LIC42] Zilionis R, Nainys J, Veres A, Savova V, Zemmour D, Klein AM, Mazutis L. 2017 Single-cell barcoding and sequencing using droplet microfluidics. Nat Protoc 12: 44–73. 10.1038/nprot.2016.154 27929523

[GR253047LIC43] Zong C, Lu S, Chapman AR, Xie XS. 2012 Genome-wide detection of single-nucleotide and copy-number variations of a single human cell. Science 338: 1622–1626. 10.1126/science.1229164 23258894 PMC3600412

