# Supplementary material for: Copolymerization of single-cell nucleic acids into balls of acrylamide gel
Source: Genome Res. 2020 Jan;30(1):49–61. doi: 10.1101/gr.253047.119 (PMC6961581; doi:10.1101/gr.253047.119)
Supplement: Supplemental Material [file supp_30_1_49__index.html]

Copolymerization of single-cell nucleic acids into balls of acrylamide gel — Supplemental Material 

# Copolymerization of single-cell nucleic acids into balls of acrylamide gel

## Supplemental Material

- Supplemental\_Figures.pdf
- Supplemental\_Tables.pdf
- Supplemental\_Text.pdf
- Supplemental\_Methods.pdf
- Supplemental\_Data\_S1.xlsx
- Supplemental\_Data\_S2.xlsx
- Supplemental\_Data\_S3.xlsx
- Supplemental\_Data\_S4.xlsx
- Supplemental\_Data\_S5.xlsx
- Supplemental\_Data\_S6.xlsx
