# Supplementary material for: Copolymerization of single-cell nucleic acids into balls of acrylamide gel
Source: Genome Res. 2020 Jan;30(1):49–61. doi: 10.1101/gr.253047.119 (PMC6961581; doi:10.1101/gr.253047.119)
Supplement: Supplemental Material [file supp_gr.253047.119_Supplemental_Figures.pdf]

Supplemental Figure S1

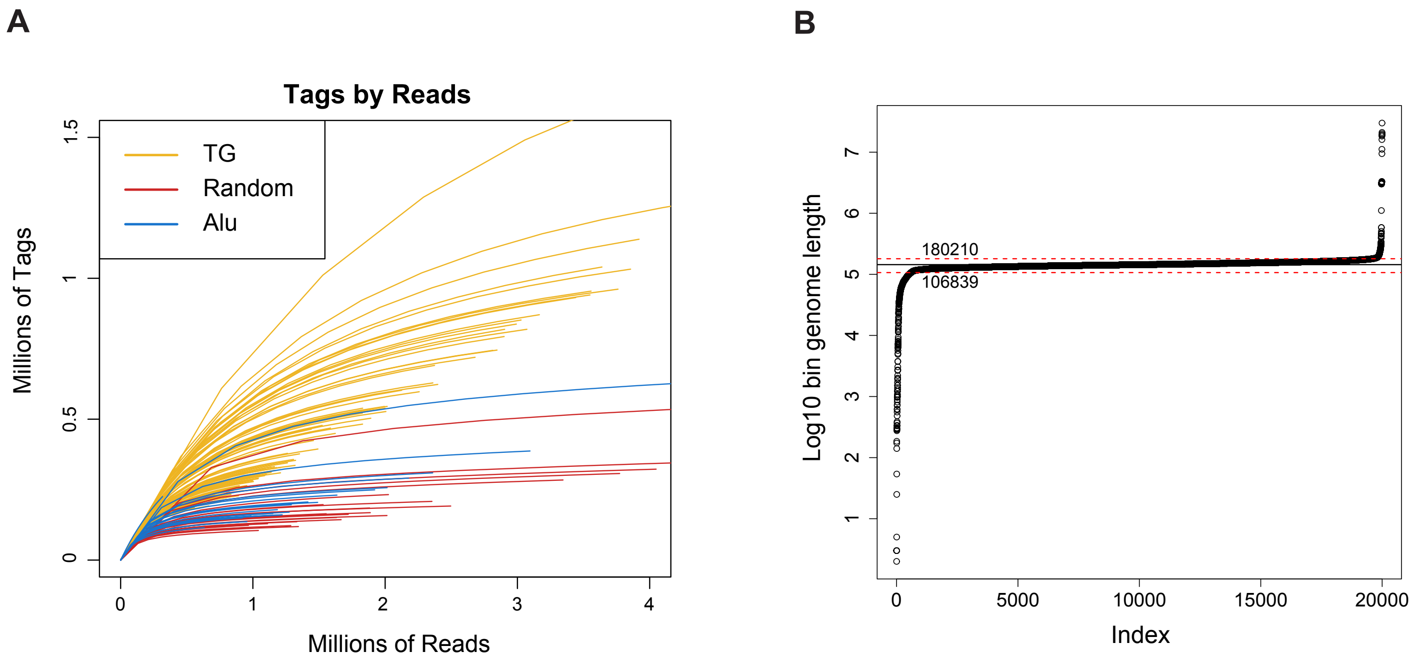

Supplemental Figure S1. The features of random TG primer for DNA capture. (A) Comparison of the three different primers (TG primer, random N primer, and *Alu* sequence primer) showing that TG primer can capture more unique templates than the other two primers given the same number of sequencing reads. Each line is a read-downsampling curve from a DNA BAG. (B) Sorted empirical bin size distribution based on TG primers showing that TG primers relatively randomly capture genomic DNA at this resolution (20k-bins). The bin boundaries were determined empirically from the SKN1 data to generate a uniform distribution for the number of tags mapping to each bin assuming a constant copy number. 95% of the bin sizes are between the two red dash lines.

Supplemental Figure S2

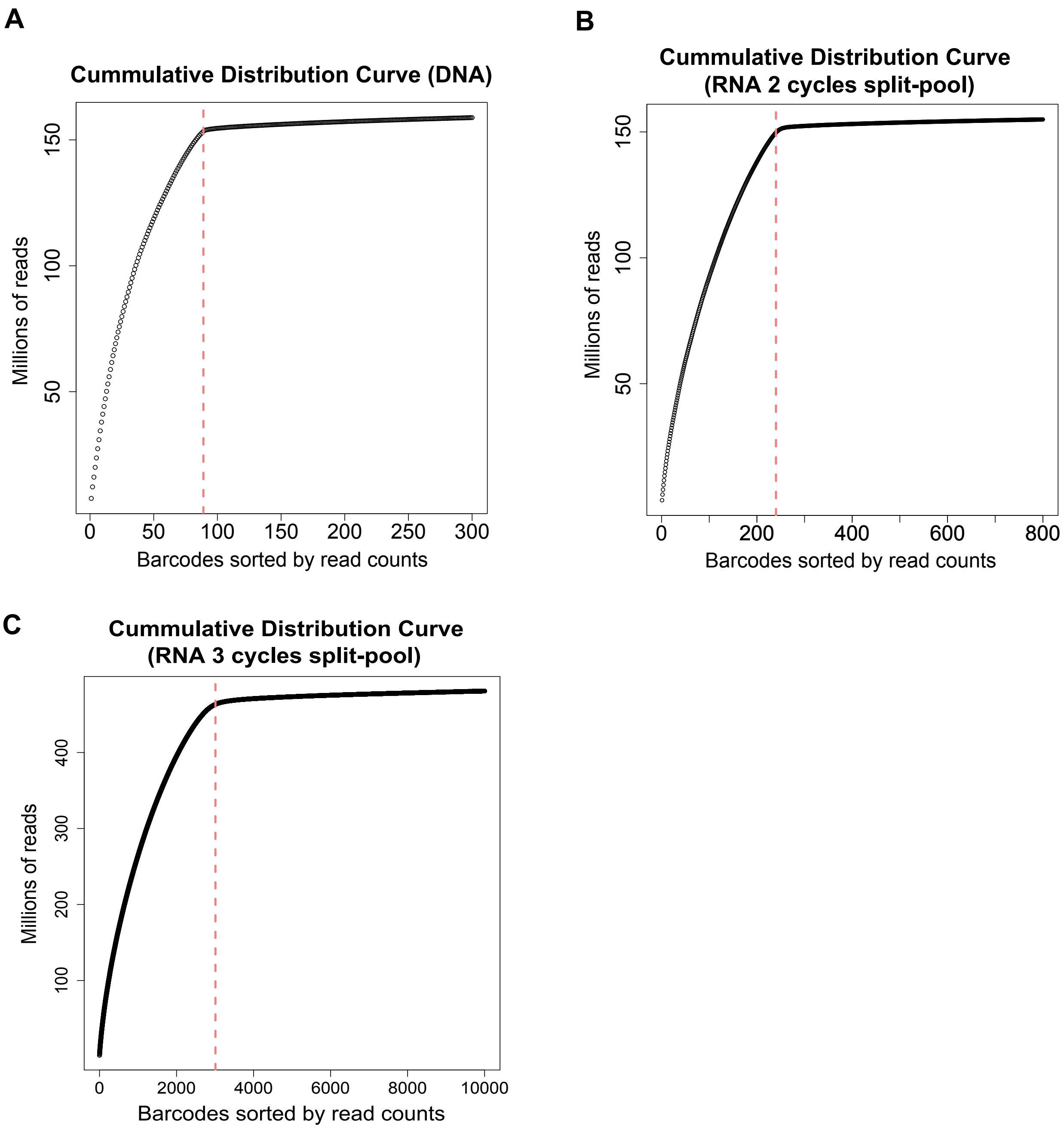

Supplemental Figure S2. Cumulative distribution curves of mapped reads in BAG experiments. (A) snDNA BAG experiment using SKN1 nuclei. A vertical line crossing the turning point indicating 88 nuclei in this experiment. (B) scRNA BAG experiment using a mixture of SKN1 and SK-BR-3 cells based on 2 cycles of split-pool. A vertical line crossing the turning point indicating 235 cells in this experiment. (C) scRNA BAG experiment using a mixture of SKN1 and SK-BR-3 cells based on 3 cycles of split-pool. A vertical line crossing the turning point indicating 3,010 cells in this experiment.

Supplemental Figure S3

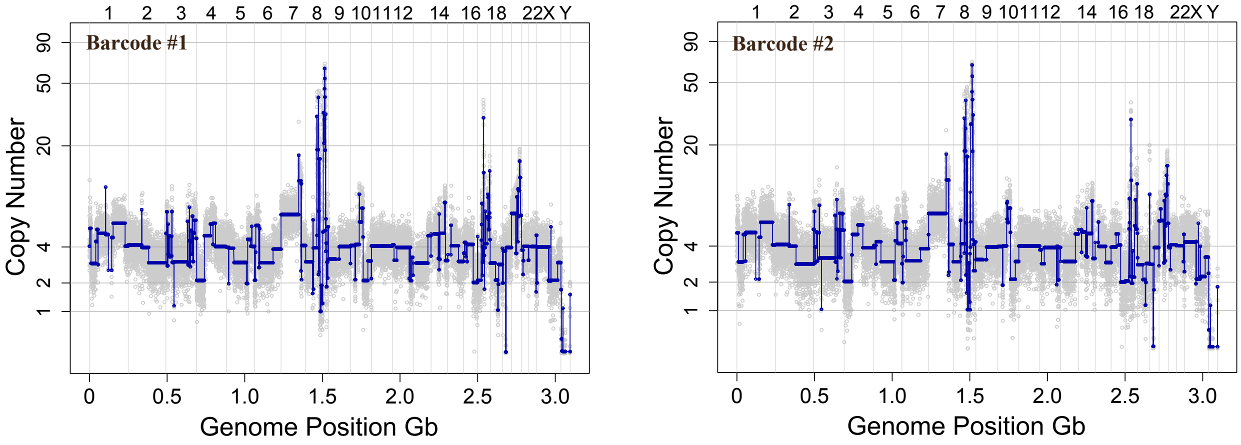

Supplemental Figure S3. Two 20k-bin SK-BR-3 copy number profiles from the 4-nuclei mixing experiment.

Supplemental Figure S4

A

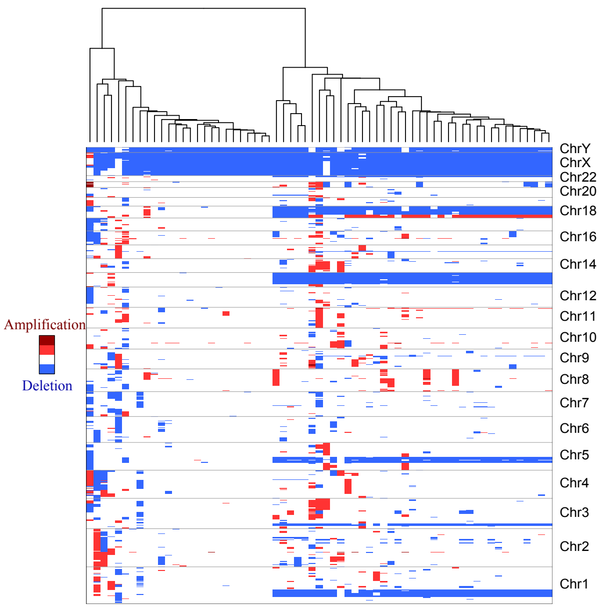

B

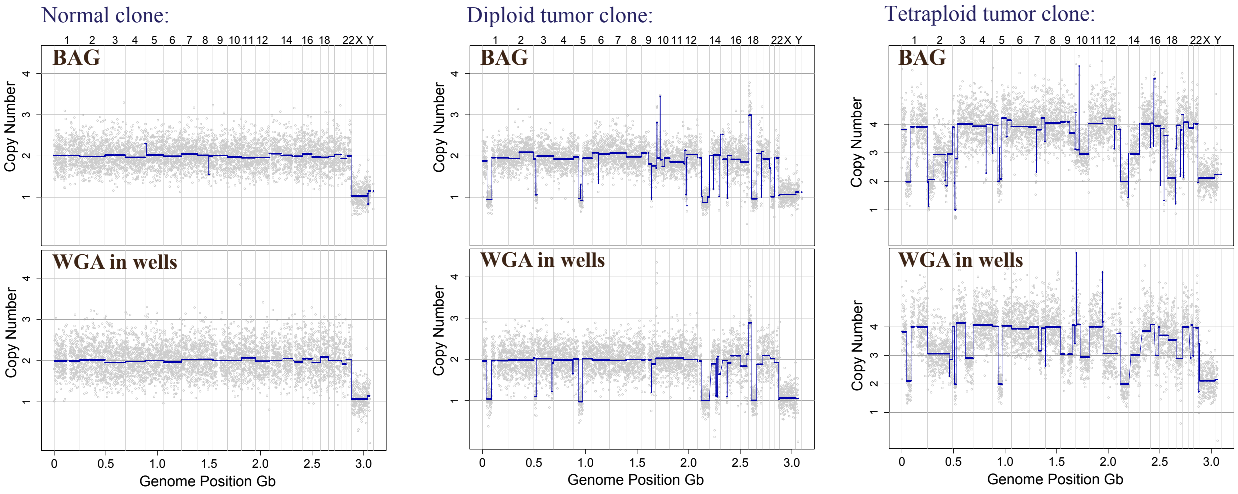

Supplemental Figure S4. (A) Hierarchical clustering of the Gleason 9 frozen prostate tumor based on BAG method. (B) Comparison of 5k-bin copy number profiles from BAG method and well-based single-cell WGA method on a normal clone and two tumor clones.

Supplemental Figure S5

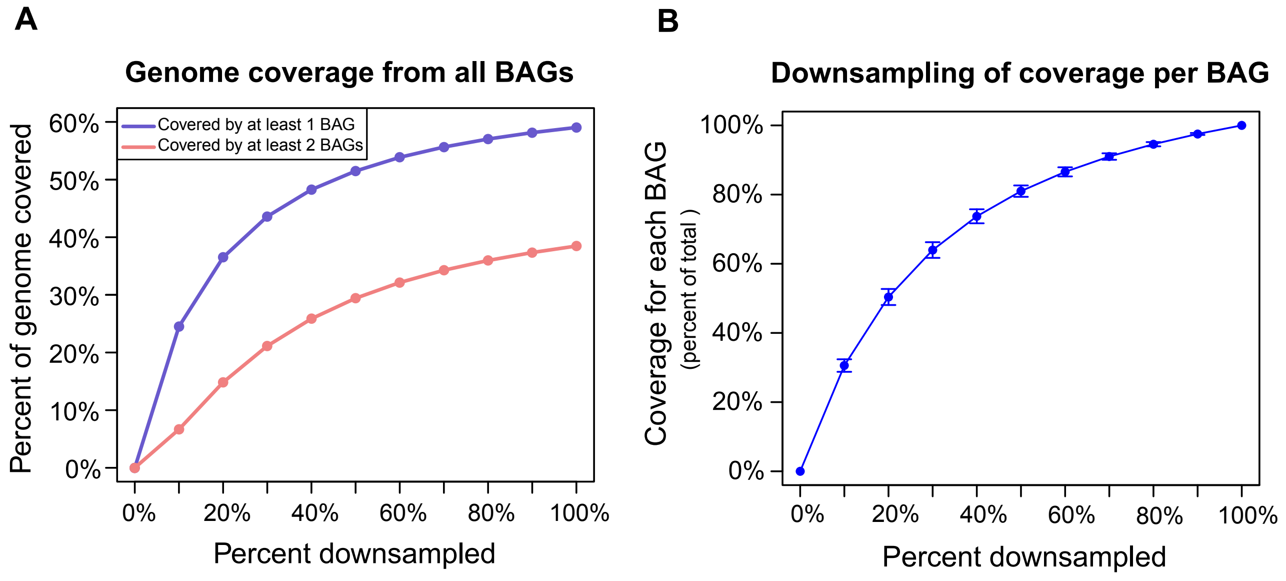

Supplemental Figure S5. Downsampling of the reads from SKN1 snDNA BAG experiment. We randomly choose 10%, 20%, 30%, ... , 90%, 100% of the reads for downsampling. At 100% downs-ampling, we use all the reads and the reads per template (RPT) is about 3.5. (A) Genome coverage by at least one BAG (blue) or at least two BAGs (red) at each downsampling position. (B) Coverage for each of the 88 BAGs relative to its total coverage on downsampling. Error bars indicate the standard deviation on each downsampling position for the 88 BAGs.

Supplemental Figure S6

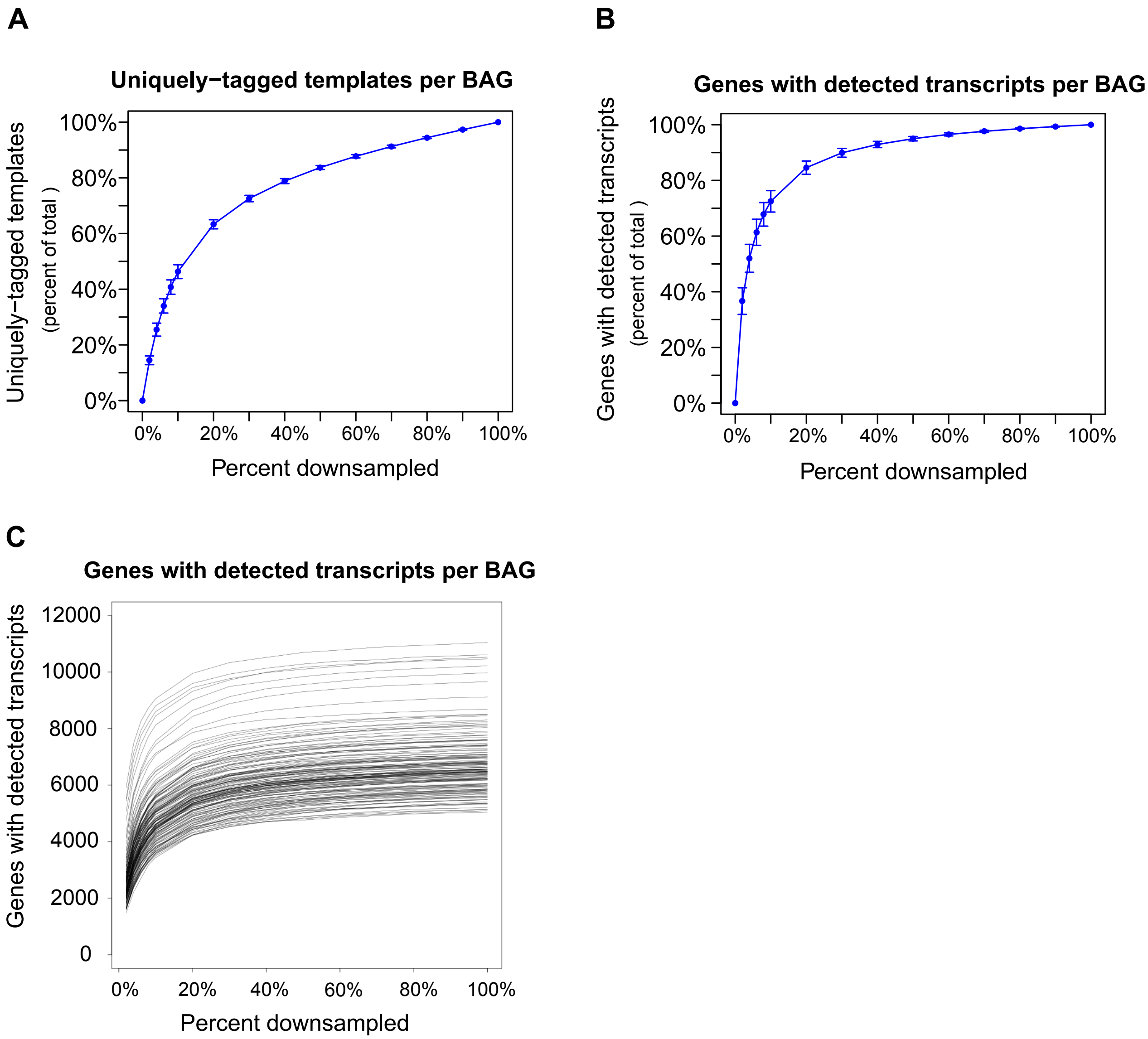

Supplemental Figure S6. Downsampling of the reads from SKN1 cells in SKN1 SK-BR-3 mixing scRNA BAG experiment. We randomly chose 2%, 4%, 6%, 8%, 10%, 20%, 30%, ... , 90%, 100% of the reads for downsampling. At 100% downsampling, we use all the reads and the reads per template (RPT) is about 6. (A) Percentage of transcripts captured of each SKN1 BAG relative to its total coverage on downsampling. Error bars indicate the standard deviation on each downsampling position. (B) Percentage of genes captured of each SKN1 BAG related to its total coverage on downsampling. (C) The number of genes each SKN1 BAG captured at each downsampling percentage.

Supplemental Figure S7

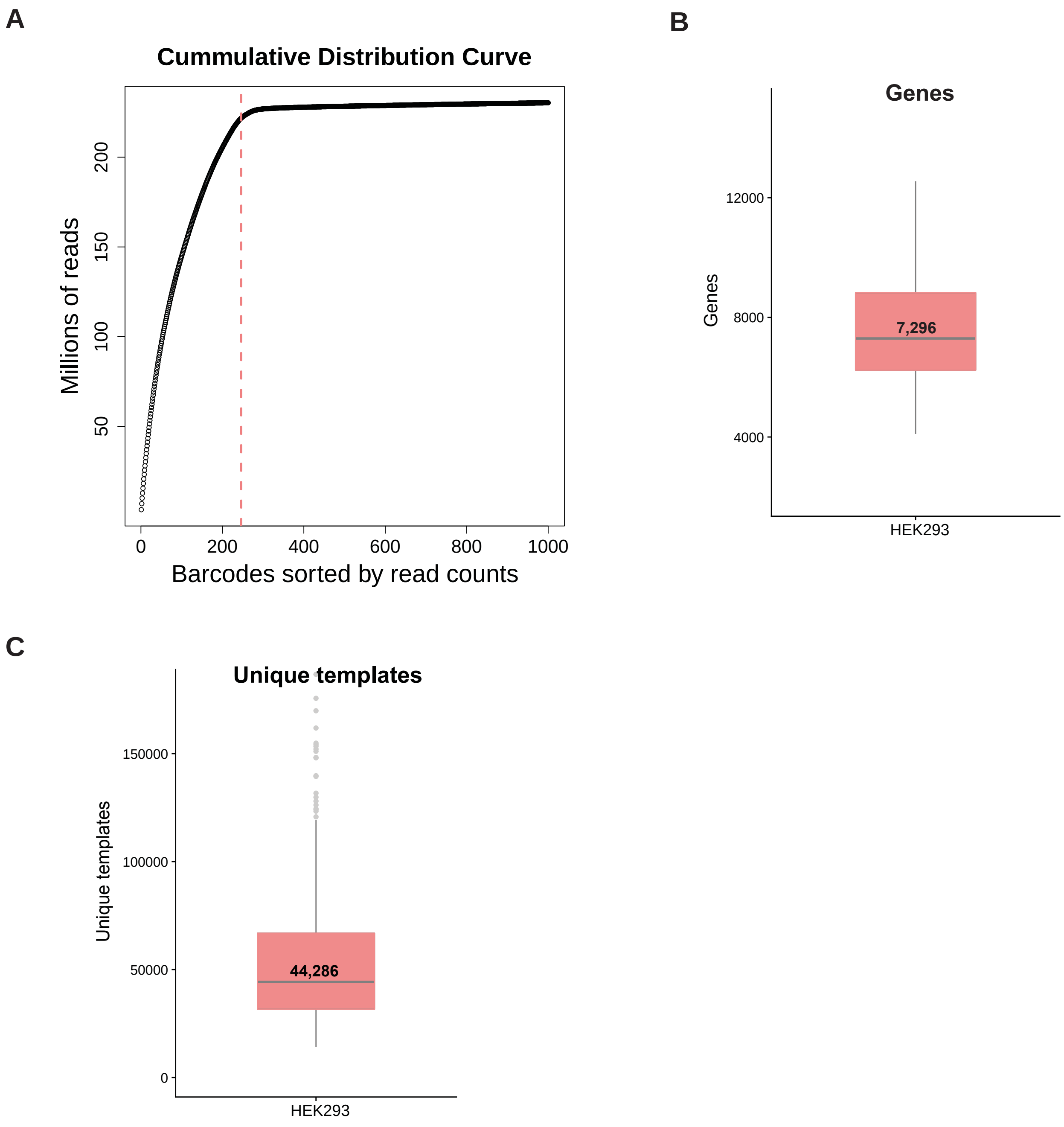

Supplemental Figure S7. scRNA BAG-seq using HEK293 cells. (A) We identified 245 barcodes on the left of the turning point of the cumulative distribution curve. (B) Boxplot showing the number of genes captured per HEK293 cell. (C) Boxplot showing the number of unique templates captured per HEK293 cell.
