# Supplementary material for: Copolymerization of single-cell nucleic acids into balls of acrylamide gel
Source: Genome Res. 2020 Jan;30(1):49–61. doi: 10.1101/gr.253047.119 (PMC6961581; doi:10.1101/gr.253047.119)
Supplement: Supplemental Material [file supp_gr.253047.119_Supplemental_Tables.pdf]

Supplemental Table S1

	BAG barcodes (sorted by total unique templates)	Total read pairs	Mapped read pairs	Unique templates	Reads per template	Genome coverage
1	ATTCCTCTACT	16077933	7635336	2087974	3.66	8.37%
2	CAAAGTGTTCT	9105339	4550849	1315784	3.46	5.62%
3	CATAATTCAAG	7418418	3919711	1164651	3.37	5.03%
4	CCTAGTTGAAC	7039734	3639037	1062977	3.42	4.65%
5	GTACTAAGAAG	7276520	3855493	1054858	3.65	4.54%
6	ATTCCTTCAGA	7108210	3760542	983943	3.82	4.28%
7	CAGAAGTCATC	6901533	3556979	976760	3.64	4.25%
8	GATATTAATAT	6722759	3548833	962843	3.69	4.19%
9	CACATAGCAAC	6779536	3440941	952106	3.61	4.17%
10	AGTTCGCGTGA	5784957	3165964	889878	3.56	3.88%
11	AAGTCATCTTA	5474677	3027753	869502	3.48	3.80%
12	AGCAGTGCTAA	5787047	2992010	856011	3.50	3.82%
13	AATTAGGCAGT	5332006	2903604	836609	3.47	3.68%
14	CAGAAGTCACT	5786830	3071809	836285	3.67	3.68%
15	AAGATGAGTGT	5419152	2899824	811577	3.57	3.57%
16	ATTCCTCGTCT	5454782	2845908	762022	3.73	3.38%
17	CCTTCAGTAGC	5248430	2813452	759570	3.70	3.33%
18	GGAACTTTAGG	5007195	2680142	736275	3.64	3.26%
19	CAAGCAACTAG	4461881	2373543	705348	3.37	3.12%
20	GTGTCTTCAAG	4213796	2360712	644147	3.66	2.89%
21	CAGAAGGTAAT	4806294	2397885	637347	3.76	2.85%
22	GTGTCTACAGC	4049982	2124910	616135	3.45	2.79%
23	AGCAACGTTAG	4057289	2257777	610460	3.70	2.69%
24	AACGTACTTGT	3537936	2015630	558227	3.61	2.50%
25	GTGATGAGTTA	3819212	1994950	557658	3.58	2.51%
26	CATAGACAAGT	3748126	1999150	551095	3.63	2.47%
27	ATCGTGCAAGT	3427397	1830061	550647	3.32	2.50%
28	CCAATAATACG	3707658	1893947	549562	3.45	2.48%
29	ATGGCTATTGG	3620876	1915009	545654	3.51	2.46%
30	AAGGTTAGAGA	3426121	1899547	541318	3.51	2.39%
31	ATGAATTGAGT	3629501	2005908	539979	3.71	2.43%
32	GGTCTATCTGT	3304347	1828523	533038	3.43	2.39%
33	CATTTGACTGA	3351552	1891525	514494	3.68	2.28%
34	AACGTATGATA	3383189	1829280	493420	3.71	2.23%
35	ATGGAAATTCC	2853257	1524108	488656	3.12	2.22%
36	ATCAAGTATTC	2944450	1586526	478058	3.32	2.15%
37	AGTGTGTATTC	2624449	1442040	474159	3.04	2.15%
38	CTCTCTACAAT	2973647	1623298	471142	3.45	2.16%
39	CCTAAGGAAGA	2678477	1458433	433208	3.37	1.97%
40	AAAGGGTCAGA	2490914	1491880	401993	3.71	1.77%
41	AAGATGCAACG	2448387	1352779	388644	3.48	1.79%
42	ATGGAATATAG	2377397	1261700	388248	3.25	1.80%
43	ATGGCTGTAAT	2429371	1307379	364989	3.58	1.69%
44	CATTTGTTACC	2393986	1322912	361597	3.66	1.65%
45	CCAATAACTCT	2201114	1261080	352664	3.58	1.58%
46	GGTTCTCTTCG	2498434	1319128	344129	3.83	1.60%
47	CATAATAATAT	2123323	1162943	339614	3.42	1.54%
48	CTCTCTAGTGT	2110745	1137844	338078	3.37	1.54%
49	GATTATGAATC	2139870	1179318	335994	3.51	1.52%

	BAG barcodes (sorted by total unique templates)	Total read pairs	Mapped read pairs	Unique templates	Reads per template	Genome coverage
50	ATAGTTTGTAA	2091657	1212518	335911	3.61	1.53%
51	CAGATTCGTTC	1964080	1087732	332221	3.27	1.48%
52	CATAGACGACA	2202146	1170726	325761	3.59	1.48%
53	ATAGTTTATCT	2081229	1154891	324171	3.56	1.47%
54	GGAACTGTTCT	2182498	1138460	318608	3.57	1.46%
55	CAGAAGTGTAA	2165475	1208916	317243	3.81	1.45%
56	CCTAAGTATCT	1905296	1053861	311570	3.38	1.43%
57	AGGTTTGTTAG	2041178	1102441	311090	3.54	1.42%
58	CAAAGTCTTGT	1971732	1090411	309564	3.52	1.42%
59	AACTTGGATTA	1810925	1021712	309223	3.30	1.38%
60	CCTTCAATTGG	2018826	1088956	307253	3.54	1.40%
61	CCTGAATCACT	1773178	1050324	302989	3.47	1.37%
62	CAAAGTGAATC	2012251	1041132	298023	3.49	1.37%
63	AGCAGTGTAGC	1954141	1039979	295275	3.52	1.37%
64	CAAAGTGATCG	1946793	998403	289290	3.45	1.32%
65	GTGTCTGGTCA	1892513	1004133	284574	3.53	1.32%
66	AGGTACTAAGC	1719227	954241	269990	3.53	1.23%
67	CTAACAGGTCA	1597603	897688	269660	3.33	1.22%
68	GGAACTTAATG	1632807	978295	268462	3.64	1.24%
69	AAGATGTAATG	1636922	965599	266950	3.62	1.24%
70	AGGTTTTATTC	1760545	950448	266459	3.57	1.22%
71	CATACGCGTTC	1612978	863582	264244	3.27	1.20%
72	CACATACGTGA	1736304	930170	251970	3.69	1.16%
73	AGTACATAACA	1388166	824650	250210	3.30	1.14%
74	ATTGACTGTGG	1333091	805572	250105	3.22	1.14%
75	AGCAGTTATTC	1615960	834235	238377	3.50	1.11%
76	GATGGAGCTAA	1265552	712311	236392	3.01	1.08%
77	CTTAGGTTATT	1599295	891261	233486	3.82	1.06%
78	ATGAGGACAAT	1436007	803602	232910	3.45	1.05%
79	GGTAATATTCC	1293924	744541	229005	3.25	1.03%
80	ATCGCATGACG	1272915	734017	225078	3.26	1.02%
81	CTTAGGCGATG	1266206	794914	220980	3.60	1.02%
82	GTCAGTAGATC	1135166	639652	214317	2.98	0.98%
83	CAAAGTGTAAT	1442942	760270	212993	3.57	0.99%
84	GGGAAAAGTCG	1101254	606382	200223	3.03	0.92%
85	GGTCTATATAG	1173143	667057	196796	3.39	0.91%
86	GGGAAACAAGT	991437	557538	188464	2.96	0.84%
87	AGCAGTCTAAG	1236589	666013	187459	3.55	0.87%
88	GGTTTCGTAAT	847978	488088	162480	3.00	0.75%

Supplemental Table S1. The read pairs, mapped read pairs, uniquely-tagged templates, mapped reads per template (RPT), and the coverage of the genome for each of the 88 BAGs in SKN1 single-nucleus DNA BAG-seq experiments.

Supplemental Table S2

Source base	Destination base	Error rate in at least 1 BAG	Error rate in at least 2 BAGs
A	C	1.4E-04	1.1E-06
A	G	2.4E-04	3.5E-06
A	T	7.6E-04	2.1E-06
C	A	1.1E-03	3.7E-06
C	G	2.9E-03	1.2E-05
C	T	6.9E-04	4.6E-06
G	A	6.9E-04	4.8E-06
G	C	2.9E-03	1.2E-05
G	T	1.1E-03	3.9E-06
T	A	7.6E-04	2.0E-06
T	C	2.4E-04	3.6E-06
T	G	1.4E-04	1.3E-06

Supplemental Table S2. Post error correction (errors corrected by multiple reads with consensus in each BAG), the chance of an error appearing in at least 1 BAG or in at least two BAGs.

Supplemental Table S3

Barcode	SKN1-SNV	SK-BR-3-SNV	Minority Ratio	Barcode	SKN1-SNV	SK-BR-3-SNV	Minority Ratio
CCTAGTCGTCT	17	2586	0.65%	CATAGATGATA	572	4	0.69%
ATAACGATACG	8	2598	0.31%	ATGGCTTGACG	675	2	0.30%
GAAACGCGATG	0	1478	0.00%	CAGATTTGATA	862	1	0.12%
ATTCAGTCTTA	5	3045	0.16%	ATCGCATCATC	703	3	0.42%
ATTCCTAATTG	10	1959	0.51%	AAAGGGGCATA	952	4	0.42%
ATTGTATCTAC	17	7383	0.23%	CATCCTACTGA	560	1	0.18%
CCAATAAGTGT	4	2889	0.14%	GTGTCTGCACG	890	0	0.00%
GGTTTCCATAA	3	2212	0.14%	AATCGAGGTTG	575	4	0.69%
AGGTACGCAAC	4	3078	0.13%	CTTAGGCGTTC	521	3	0.57%
GAAGGTAGTTA	7	2550	0.27%	AAGTCAACTGA	622	7	1.11%
GGTCTATCTCG	3	1757	0.17%	CAGTAAGGTAT	737	7	0.94%
CATAGAGGTCA	4	2437	0.16%	GGAACTTTACC	949	6	0.63%
ATTGACAGTAC	4	2659	0.15%	CATTTGCTATC	762	1	0.13%
CAAAGTGCATA	2	2752	0.07%	AATCGAAGAGA	596	5	0.83%
CCAATAAGTAC	11	3229	0.34%	AGTGTGTAACA	827	7	0.84%
GGGAAACGACA	16	6120	0.26%	CTAACAAATGC	693	3	0.43%
GAAACGACTTC	3	5604	0.05%	GAAACGTGTGG	595	0	0.00%
CTCTCTCTAGA	5	2360	0.21%	GAGTTCCTTAC	1306	3	0.23%
AGTATTTTAGG	4	2005	0.20%	AAGTCACAACG	485	1	0.21%
AGGTACGTACA	13	5413	0.24%	CTCTCTAGAAG	529	1	0.19%
AGCTAGACACA	12	3129	0.38%	GTGTCTATAAC	1831	0	0.00%
AAGCATAGAGA	9	4922	0.18%	CCAATAAATCA	757	5	0.66%
AAGCATAGTCG	3	2547	0.12%	CAAGCATAACA	608	1	0.16%
ATAACGGATGT	3	2105	0.14%	AGGTTTCATAA	641	3	0.47%
CAAAGTTTAGG	3	2734	0.11%	CAGAAGAATCA	531	2	0.38%
AGGTTTTCTCG	8	4661	0.17%	CACATAGTACA	730	4	0.54%
GGTTCTTTACC	5	2781	0.18%	AGAGCACTTCG	876	4	0.45%
ATATTACTTAC	21	9414	0.22%	CTCTCTTGATA	838	4	0.48%
GATGGAGATAC	8	4957	0.16%	GATATTCGACA	832	4	0.48%
AAGGTTGAATC	6	4992	0.12%	CTAACAGTTGA	1713	5	0.29%
ATTGTAACACA	6	3446	0.17%	CTCAATTTATT	778	1	0.13%
AAGTCATCTAC	13	6596	0.20%	CAACTTGATCG	871	1	0.11%
GGTTCTTGTAA	11	3283	0.33%	CATTTGAATCA	852	0	0.00%
AAAGGGGCACG	6	2821	0.21%	AGAGCAGGTAT	559	1	0.18%
ATTAGTGGTTG	7	2705	0.26%	CAGATTGCATA	694	5	0.72%
				CTAACATGTGG	490	1	0.20%
				CCTAAGATTAA	628	2	0.32%
				CAACACAGTCG	610	4	0.65%

Supplementary Table S3. The number of SKN1-specific SNVs, SK-BR-3-specific SNVs and minority SNV ratio in each SKN1 or SK-BR-3 BAG from the 4-nuclei mixing DNA BAG-seq experiment.

Supplemental Table S4

	male count	female count	binomial test code	p-value
**Cluster #0**	192	136	binom.test(192, (192+136), p = 0.628)	**0.1101**
**Cluster #1**	166	55	binom.test(166, (166+55), p = 0.628)	**0.0001201**
**Cluster #2**	71	36	binom.test(71, (71+36), p = 0.628)	**0.4848**
**Cluster #3**	45	25	binom.test(45,(45+25), p = 0.628)	**0.9017**
**Cluster #4**	26	22	binom.test(26,(26+22), p = 0.628)	**0.2334**
**Cluster #5**	18	24	binom.test(18,(18+24), p = 0.628)	**0.009998**
**Cluster #6**	10	15	binom.test(10,(10+15), p = 0.628)	**0.02252**
**Cluster #7**	12	7	binom.test(12,(12+7), p = 0.628)	**1**
**Total Nuclei**	540	320	540 / (540 + 320) = 0.628	

Supplemental Table S4. Binomial test showing the difference in nuclei numbers between sexes in each cluster. The red color highlights the most significant difference among all the clusters.
