# Supplementary material for: Copolymerization of single-cell nucleic acids into balls of acrylamide gel
Source: Genome Res. 2020 Jan;30(1):49–61. doi: 10.1101/gr.253047.119 (PMC6961581; doi:10.1101/gr.253047.119)
Supplement: Supplemental Material [file supp_gr.253047.119_Supplemental_Text_.pdf]

Supplemental Text

Comparison to other high-throughput methods on the following aspects using HEK293 cell

1. **RNA coverage**: Based on (Ding et al. 2019) (Fig. 2A,B), we chose to use HEK293 because it serves as a common test.

   BAG-seq has a median of **7,296** genes from single HEK293 cells at the current sequencing depth (Supplemental Fig. S7B), and has a median of **5,153** genes if using 10% of the reads for downsampling to achieve a median reads per cell of 70K. At the sequencing depth of 50K-100K reads per cell, 10x Chromium (v2), Drop-seq, Seq-Well, inDrops have **4,374, 2,581, 3,936, 1,632** genes, respectively, from their replicate with the best median.

   BAG-seq has a median of **44,286** unique templates from single HEK293 cells at the current sequencing depth (Supplemental Fig. S7C), and has a median of **21,121** unique templates if using 10% of the reads for downsampling to achieve a median reads per cell of 70K. At the sequencing depth of 50K-100K reads per cell, 10x Chromium (v2), Drop-seq, Seq-Well, inDrops have **18,879, 5,106, 10,154, 3,011** unique templates, respectively, from their replicate with the best median.

2. **Barcode collision rate**: At 250-cell level, BAG-seq has a barcode-collision rate of **0.85%**, and at 3,000-cell level BAG-seq has a rate of **0.67%**. Based on (Ding et al. 2019) (Fig. 2C), at 3,000-cell level, the 10x Chromium v2's barcode collision (multiplet) rate is ~ **3.5%** (replicate #1) or **1.2%** (replicate #2); Drop-seq's is around **3.3%** (replicate #1) or **3.5%** (replicate #2); Seq-Well's is around **2.6%** (replicate #1) or **3.2%** (replicate #2), and inDrop's is around **8.0%** (replicate #1) or **3.4%** (replicate #2).

Reference:

Ding J, Adiconis X, Simmons SK, Kowalczyk MS, Hession CC, Marjanovic ND, Hughes TK, Wadsworth MH, Burks T, Nguyen LT, et al. 2019. Systematic comparative analysis of single cell RNA-sequencing methods. *BioRxiv*: 632216.
