# Supplementary material for: Copolymerization of single-cell nucleic acids into balls of acrylamide gel
Source: Genome Res. 2020 Jan;30(1):49–61. doi: 10.1101/gr.253047.119 (PMC6961581; doi:10.1101/gr.253047.119)
Supplement: Supplemental Material [file supp_gr.253047.119_Supplemental_Methods_.pdf]

Supplemental Method S1

Single-Cell DNA BAG-seq Laboratory Protocol

Equipment (similar to Drop-seq/DroNc-seq set-up):

Name	Company	Catalog Number
Inverted Microscope	Motic	AE31
Legato 100 Syringe Pumps (3)	KD Scientific	788100
3 mL Syringes	Becton Dickinson	309657
10 mL Syringes	Becton Dickinson	302995
PE-2 Tubing	Scientific Commodities	BB31695-PE/2
26 Gauge Needles	Becton Dickinson	305111
22 Gauge Needles	Becton Dickinson	305155
16 Gauge Needles	Becton Dickinson	305197
PDMS co-flow microfluidic droplet generation device “DroNc-seq device”	Nanoshift, LLC	
40 µm cell strainers	Corning	352340
70 µm cell strainers	Corning	431751
100 µm cell strainers	Corning	352360

Primers:

Name	Sequence
Linker TG	/5ACryd//iSp18/TGTGTTGGGTGTGTTTGGKKKKKKKGKKKKKKKKNN
Adapter1-BC1-vt-#DNAx	ACGCAGAGTCCGCTC <barcode1> NNNN CA+T+G (detailed sequences at bottom)
Adapter2-BC2-Adapter1-#DNAx	GTCTCGTGGGCTCGGAGATGTGTATAAGAGACAG <barcode2> ACGCAGAGTCCGCTC (detailed sequences at bottom)
TG primer	TGTGTTGGGTGTGTTT*G*G
P5-TG	AATGATACGGCGACCACCGAGATCTACAC GGAGATGTG TGTGTTGGGTGTGTTT*G*G
Read1-TG	GGAGATGTG TGTGTTGGGTGTGTTTGG

Chemical Reagents:

Name	Company	Catalog Number
**Nuclei Preparation**		
Nuclei PURE lysis buffer (only for nuclei prep from tumor sample)	Sigma-Aldrich	NUC201-1KT
Nuclei EZ lysis buffer	Sigma-Aldrich	NUC101-1KT
10X PBS	Thermo Fisher Scientific	AM9624
UltraPure BSA	Thermo Fisher Scientific	AM2618
Trypsin-EDTA(0.25%, phenol red)	Thermo Fisher Scientific	25200056
**Aqueous Phase 2**		
Acrylamide/bis-acrylamide, 40% solution	Sigma-Aldrich	A9926
Acrylamide solution, 40%	Sigma-Aldrich	A4058
0.5 M EDTA	Thermo Fisher Scientific	AM9260G
20% Sarkosyl	Sigma-Aldrich	L7414

0.1M DTT	Thermo Fisher Scientific	707265ML
Ammonium persulfate	Sigma-Aldrich	09913-100G
10% NP-40	Thermo Fisher Scientific	28324
Proteinase K	Sigma-Aldrich	P4850
**Oil Phase**		
HFE-7500	Oakwood Chemical	051243
FC-40	Sigma-Aldrich	F9755
008-Fluorosurfactant	Ran Technologies	008-FluoroSurfactant-5G
TEMED	Bio-Rad	1610801
**Post-Droplet Generation**		
Mineral Oil	Sigma-Aldrich	69794-500ML
20X SSC	Thermo Fisher Scientific	15557044
Perfluorooctanol (PFO)	Sigma-Aldrich	370533
10 mM dNTP	Sigma-Aldrich	11814362001
DNA Polymerase I, Large (Klenow) Fragment	New England BioLabs	M0210M
Exonuclease I	New England BioLabs	M0293
NlaIII	New England BioLabs	R0125L
Klenow Fragment (3-5 exo minus)	New England BioLabs	M0212L
Quick Ligase	New England BioLabs	M2200
KAPA HiFi HotStart ReadyMix	Roche	KK2602
Agencourt AMPure XP beads	Beckman Coulter	A63880
Nextera XT DNA Library Prep Kit	Illumina	FC-131-1024

Chemical Solutions to Prepare

PBS-BSA (1X PBS, 0.05% BSA)
20% Surfactant in HFE-7500
20% Surfactant in FC-40
10% Ammonium persulfate
6X SSC
TE-SDS (10 mM Tris pH 8.0, 1 mM EDTA, 0.5% SDS)
STOP-25 (10 mM Tris pH 8.0, 25 mM EDTA, 0.1% Tween-20, 0.1 M KCl)
STOP-10 (10 mM Tris pH 8.0, 10 mM EDTA, 0.1% Tween-20, 0.1 M KCl)
STOP-1 (10 mM Tris pH 8.0, 1 mM EDTA, 0.1% Tween-20, 0.1 M KCl)
HBW (10 mM Tris pH 8.0, 1 mM EDTA, 0.1% Tween-20)

scDNA BAG-seq Experiment:

Nuclei Preparation from Tumor Sample

1. Keep reagents on ice and tumor sample on dry ice. Set centrifuge temperature to 4°C.
2. Use scalpels to mince sample on a sterile plate. Add 500 µL of Nuclei PURE lysis buffer onto the sample.
3. Transfer sample using pipette from plate to a glass tissue grinder. Use pestles to further mince the sample while keeping the grinder on ice.
4. Add 3.5 mL of EZ lysis buffer, then transfer all to a 15 mL conical. Pipet up and down.
5. Once in 15 mL conical tube, keep on ice for 5 minutes.
6. Centrifuge the conical tube at 500 rcf for 5 minutes at 4°C, then discard supernatant. Re-suspend the pellet in 700 µL of EZ lysis buffer. Pipet up and down, and gently vortex.
7. Add 3.3 ml of EZ buffer and pipet up and down. Wait 5 minutes. Gently vortex. Spin down using the same settings, then discard supernatant.
8. Add 600 µL of PBS-BSA, pipet up and down, pass through a 70 µm filter, and count nuclei.
9. Use PBS-BSA to create a final dilution of 2.5E5 nuclei in 1 mL volume.

Nuclei Preparation from Cell Culture

1. Follow standard cell harvest protocol to harvest cells from plates. Collect cell solution in a 15 mL conical and spin down at 500 rcf for 5 minutes.
2. Discard supernatant, then re-suspend pellet in 8 mL of 1x PBS. Spin once more using same settings, and aspirate supernatant.
3. Add 4 ml of EZ lysis buffer into pellet, pipet up and down, and wait 5 minutes. Lightly vortex, spin down again, and discard supernatant
4. Add 700 µL of EZ lysis buffer to break pellet, pipet up and down, lightly vortex. Then add 3.3 mL of EZ lysis buffer, pipet up and down, and keep on ice for 5 minutes. Lightly vortex, spin down, and discard supernatant.
5. Add 1.5 mL of PBS-BSA. Pass through a 40 µm filter, and count.
6. If preparing a 1:1 mix of the two cell types, use PBS-BSA to create a final dilution of 2.5E5 cells in 1 mL.

Oil Phase Preparation

1. Combine the following; vortex to mix (*enough for 2 samples*):

600 µL	20% surfactant
9.6 µL	TEMED
1.8 mL	HFE7500

Aqueous Phase 2 Preparation

1. Combine the following; pipet to mix (*enough for 2 samples*):

**Aqueous Phase 2 Solution**	**Volume (µL)**	**Final Concentration**
AA/bis-acrylamide, 40%	180	
Acrylamide solution, 40%	129	
H_2O	271	
500 µM Linker TG primer	160	40 µM
0.5M EDTA	50	12.5 mM
1M Tris pH 7.5	100	50 mM
20 % Sarkosyl	10	0.1%

Proteinase K	20	
0.1M DTT	20	1 mM
10% APS	60	0.3%
**Total**	**1 mL**	

Microfluidics Setup & Oil Droplet Collection

1. Turn on microscope. Place microfluidic device on microscope stage.
2. Insert a piece of PE/2 tubing into the output well and place the other end into a waste container (15 mL conical tube).
3. Use the following syringes to collect the three solutions.
   a. **Oil Phase:** Use a 10 mL syringe with a 16 G needle to aspirate solution into syringe.
   b. **Cell Solution or Aqueous Phase 2**: Use a 3 mL syringe and a 22 G needle to aspirate solution into syringe.
4. For each syringe, change the needle to a 26 G needle and insert into PE/2 tubing.
5. Place syringes into their designated syringe pumps and insert tubing into the corresponding wells of the microfluidics chamber
   a. From left to right: Oil – Aqueous 2 – Cell Solution (Aqueous 1) – Output
6. Once all syringes and tubing are in place, set the following flow rates:
   a. **Oil Phase:** 3,000 µL/hr
   b. **Cell Solution:** 750 µL/hr
   c. **Aqueous Phase 2:** 750 µL/hr
7. Begin by first turning on the cell solution pump.
8. Once cell solution is flowing, turn on the aqueous phase 2 pump.
9. Once the interphase between cell solution and aqueous phase two has stabilized, turn on the oil phase pump and wait for the droplet formation to stabilize.
10. Once the flow has stabilized, place the output tubing on a glass slide under the microscope to assess the size uniformity of oil droplets.
11. Once droplet size is uniform, collect droplets in 300 µL of mineral oil in 1.5 mL tubes.
12. After collection, incubate BAGs at 50°C overnight.

Oil Change and Incubation

1. Discard bottom layer of the tube using 22 G needle. Add 300 µL of FC-40 oil with 5% surfactant into the bottom of the tube.
2. Incubate at 95°C for 12 minutes to denature genomic DNA.
3. Incubate at 55°C for 1 hour, then incubate at room temperature for at least 10 minutes.

Droplet Breakage

1. Remove mineral oil in top layer using pipet. Remove mineral oil in bottom layer using a 3 mL syringe and 22G needle.
2. Add 600 µL 6X SSC buffer and 150 µL perfluorooctanol (PFO) in a fume hood. Shake the tube by hand to break the droplets and release balls of acrylamide gel (BAGs).
3. Centrifuge 1000 rcf for 1.5 minutes.
4. Discard the top and bottom layer, leaving the translucent BAG layer in the tube.
5. Add 600 µL 6x SSC and shake tube multiple times. Centrifuge 1000 rcf for 1.5 minutes. Discard the top layer and any visible PFO from the bottom of the tube.

Linear Extension

1. Wash BAG layer using 1X NEB2 buffer. Pipet to mix. Centrifuge 1000 rcf for 1.5 minutes, discard the supernatant.
2. Prepare the following linear extension solution; pipet up and down to mix:

**Linear Extension Solution**	**Volume (µL)**
H_2O	830
NEB2 buffer	100
dNTP	60
DNA Polymerase I, Large (Klenow) Fragment	10
**Total**	**1 mL**

3. Transfer linear extension solution to BAG layer. Pipet up and down ~10x to mix.
4. Incubate at room temperature for 1.5 hours with rotation.
5. Incubate at 37°C for 30 minutes with rotation.
6. Stop the reaction with the following steps:
    a. Add 1:1 ratio of STOP-25 buffer. Mix by pipetting.
    b. Centrifuge at 1000 rcf for 1 minute. Discard supernatant.
    c. Add ~500 µL of STOP-25 buffer to each tube. Combine contents into one tube.
    d. Centrifuge at 1000 rcf for 1 minute. Discard supernatant.
    e. Add 1 mL of STOP-10 buffer, mix, and pass solution through 100 µm cell strainer, and transfer the solution to a new 1.5 mL Eppendorf tube.
    f. Continue to exonuclease treatment or re-suspend in 800 µL STOP-10 and store at 4°C.

--------------------Safe Stopping Point: Store BAGs in STOP-10 at 4°C overnight--------------------

Exonuclease Treatment

1. If stored in STOP-10, centrifuge 1000 rcf for 1.5 minutes and discard the supernatant.
2. Wash once with STOP-1.
3. Wash once with 1x Exo I buffer. Discard supernatant.
4. Prepare the following exonuclease treatment solution and vortex.

**Exonuclease Treatment Solution**	**Volume (µL)**
H_2O	680
10x Exo I buffer	80
Exo I enzyme	40
**Total**	**800 µL**

5. Transfer 800 µL Exonuclease Treatment Solution to the tube of BAGs; pipet to mix.
6. Incubate 37°C with rotation for 1 hour.
7. Centrifuge at 1000 rcf for 1 minute. Discard supernatant.
8. Stop the reaction with the following washes:
    a. STOP-25 twice
    b. STOP-10 once.
9. Continue to NlaIII cutting or re-suspend in 800 µL STOP-10 and store at 4°C.

--------------------Safe Stopping Point: Store BAGs in STOP-10 at 4°C overnight--------------------

NlaIII cutting

1. If stored in STOP-10, centrifuge 1000 rcf for 1.5 minutes and discard the supernatant.
2. Wash once with STOP-1.

3. Wash once with 1x CutSmart buffer.
4. Prepare the following NlaIII solution 1 and vortex.

**NlaIII Solution 1**	**Volume (µL)**
H_2O	840
10x CutSmart buffer	100
NlaIII enzyme	60
**Total**	**1 mL**

5. Transfer NlaIII Solution 1 to the tube of BAGs; pipet to mix.
6. Incubate 37°C with rotation for 1.5 hours.
7. Centrifuge at 1000 rcf for 1.5 minutes. Discard supernatant
8. Prepare the following NlaIII solution 2 and vortex.

**NlaIII Solution 2**	**Volume (µL)**
H_2O	880
CutSmart buffer	100
NlaIII enzyme	20
**Total**	**1 mL**

9. Transfer NlaIII Solution 2 to the tube of BAGs; pipet to mix.
10. Incubate 37°C with rotation for 1 hour.
11. Centrifuge at 1000 rcf for 1.5 minutes. Discard supernatant.
12. Stop the reaction with the following washes:
    a. STOP-25 twice
    b. STOP-10 once.
13. Continue to First split-pool or re-suspend in 800 µL STOP-10 and store at 4°C.

--------------------Safe Stopping Point: Store BAGs in STOP-10 at 4°C overnight--------------------

First Split-Pool

1. Preparation:
    a. If stored in STOP-10 overnight, centrifuge 1000 rcf for 1.5 minutes. Discard supernatant.
    b. Wash once with HBW buffer. Discard supernatant.
2. Prepare the following hybridization master mix and vortex.

**Hybridization Master Mix**	**Volume (µL)**
H_2O	400
2x Quick Ligase Buffer	700
dNTP	100
**Total**	**1200 µL**

    a. Transfer Hybridization Master Mix into BAG tube (assuming there is 100 µL of BAG pellet in HBW buffer). Pipet to mix.
    b. Quickly add 162.5 µL into each tube of one PCR strip. Use multipipette to transfer 13 µl of this solution to each tube in a 96-well PCR plate.
    c. Add 1 µL of 100 uM “Adapter1-BC1-vt-#DNAx” primers into each corresponding well. Each well should have a unique well-specific primer. Briefly spin down, and invert plate.
    d. Incubate at room temperature for 4 minutes in inverted position.
    e. Incubate at 50°C for 5 minutes in thermocycler with heated lid.

f. Transfer plates directly to cold racks in 4°C.
g. Keep inverted at 4°C for 10 minutes, then another 10 minutes with rotation. Follow these steps for hybridization:

3. On ice, prepare the following Extension Master Mix; vortex to mix.

Extension Master Mix	Volume (µL)
H_2O	200
2X Quick Ligase Buffer	250
Klenow 3-5 exo minus	75
Quick Ligase enzyme	75
**Total**	**600**

h. Transfer 75 µL of Extension Master Mix into each tube of one PCR strip
i. Put the plate on cold rack, and using multipipette add 6 µL of Extension Master Mix to each tube in BAG plate. Pipet up and down ~10x
j. Transfer plates to thermocycler with the following conditions:

**Gel Extension Program:**
10°C 30 minutes
22°C 10 minutes (ramp rate 0.2°C / second)

k. Incubate at room temperature with rotation for 40 minutes.
l. Incubate 37°C with rotation for 40 minutes.

4. Stop the reaction with the following:
   a. Add 100 µL STOP-25 to each well of the plate. Centrifuge plates 1000 rcf for 2 minutes at room temperature.
   b. Without disturbing the BAG pellet, discard 85 µL from **the top** of each tube.
   a. Add 5 mL STOP-25 into solution basin. Transfer the remaining BAG solutions from the plate into the solution basin (35 µL per tube).
   b. Transfer BAG solution from solution basin into a 15 mL conical tube. Centrifuge 200 rcf for 4 minutes. Discard supernatant.
   c. Add 800 µL STOP-25, pipet up and down, and transfer BAG solution into a 1.5 mL tube. Centrifuge 1000 rcf for 1.5 minutes. Discard supernatant. Re-suspend in 800 uL STOP-10 buffer.

--------------------Safe Stopping Point: Store BAGs in STOP-10 at 4°C overnight--------------------

Second Split-Pool

1. Preparation:
   a. Thaw “TG primer” and “Adapter2-BC2-Adapter1-#DNAx” barcode primers.
   b. Take out BAGs from 4°C, discard supernatant, and perform one STOP-10 wash. Discard supernatant. Re-suspend BAGs in 500 µL HBW.
2. Use a microscope to count the number of BAGs per µL.
3. Create the following TG primer/BAG mix:

TG primer/BAG mix	Volume (µL)
10 µM “TG primer”	100
BAGs in HBW	x
H_2O	$764 - x$
**Total**	**864 µL**

   a. Transfer 108 µL of TG primer/BAG mix into each tube of one PCR strip. Use multipipette to transfer 9 µL of mix into each tube of 96-well plate.

4. Add 1 µL of 96 different 10 µM “Adapter2-BC2-Adapter1-#DNAx” primers into each corresponding well. Each well should have a unique primer. Briefly spin.
5. On ice, add 10 µL of Ultra II Q5 Master Mix to each tube of 96-well plate. Pipet to mix.
6. Proceed to PCR:

Q5 Gel PCR program:
95°C 2 min
98°C 15 s
12 cycles of:
98°C 15 s
63°C 1 min
65°C 1 min
Then:
65°C 5 min
4°C infinite

7. Purify sample twice with AMPure XP beads using the following guidelines:
    a. First Pool: Pool 10 ul from each of the eight tubes in each column into a new PCR tube.

(ex. 1A-1H) into a new PCR tube, as shown.

1A	2	3	4	5	6	7	8	9	10	11	12
B											
C											
D											
E											
F											
G											
H											

1	2	3	4	5	6	7	8	9	10	11	12

(After pool: 80 µL each)

    b. First Purification: Purify these 12 samples with 1.0x ratio (80 µL) of AMPure XP beads, and elute with 25 µL of H_2O using AMPure XP protocol.
    c. Second Pool:

Pool 23 ul from three tubes into one tube.

1	2	3	4	5	6	7	8	9	10	11	12

1	2

(After pool: 69 µL each)

    d. Second Purification: Purify these four samples with another 0.8x ratio (56 µL) of AMPure XP beads, and elute with 21 µL H_2O using AMPure XP protocol.
8. Run a High Sensitivity DNA Chip on an Agilent Bioanalyzer.

Making Final Sequencing Library

1. Prepare following reaction mix for each of the samples

Final PCR Reaction Mix	Volume (µL)
Q5 Ultra Master Mix	20

10 µM “P5-TG” primer	1
10 µM N70x Nextera primer	1
DNA sample	18
**Total**	**40 µL**

2. Proceed to PCR

<u>Final PCR:</u>
95°C 20s
4 cycles of:
98°C 15 s
62°C 40 s
72°C 40 s
3 cycles of:
98°C 10 s
67°C 20 s
72°C 40 s
Then:
72°C 4 min
4°C infinite

3. Post-PCR purification: Purify sample with 0.8x ratio of Ampure XP Beads, and elute with 10 µL H_2O using AMPure XP protocol.
4. Run a High Sensitivity DNA Chip on an Agilent Bioanalyzer to check the final concentration of the sample.
5. Sequence your sample using Illumina platform with custom Read1 sequencing primer “Read1-TG”.

Barcode Sequences

First Split-Pool Barcodes

Oligo name	Sequence
**Adapter1-BC1-vt-#DNA1**	ACGCAGAGTCCGCTCCTAAGNNNNNCA+T+G
**Adapter1-BC1-vt-#DNA2**	ACGCAGAGTCCGCTCACAATNNNNNCA+T+G
**Adapter1-BC1-vt-#DNA3**	ACGCAGAGTCCGCTCTAATGNNNNNCA+T+G
**Adapter1-BC1-vt-#DNA4**	ACGCAGAGTCCGCTCCTTCGNNNNNCA+T+G
**Adapter1-BC1-vt-#DNA5**	ACGCAGAGTCCGCTCTATAGNNNNNCA+T+G
**Adapter1-BC1-vt-#DNA6**	ACGCAGAGTCCGCTCGGTCANNNNNCA+T+G
**Adapter1-BC1-vt-#DNA7**	ACGCAGAGTCCGCTCTGATANNNNNCA+T+G
**Adapter1-BC1-vt-#DNA8**	ACGCAGAGTCCGCTCCGACANNNNNCA+T+G
**Adapter1-BC1-vt-#DNA9**	ACGCAGAGTCCGCTCTGAGTNNNNNCA+T+G
**Adapter1-BC1-vt-#DNA10**	ACGCAGAGTCCGCTCATTCCNNNNNCA+T+G
**Adapter1-BC1-vt-#DNA11**	ACGCAGAGTCCGCTCGATCGNNNNNCA+T+G
**Adapter1-BC1-vt-#DNA12**	ACGCAGAGTCCGCTCTCAGANNNNNCA+T+G
**Adapter1-BC1-vt-#DNA13**	ACGCAGAGTCCGCTCCATGGNNNNNCA+T+G
**Adapter1-BC1-vt-#DNA14**	ACGCAGAGTCCGCTCTCTGTNNNNNCA+T+G
**Adapter1-BC1-vt-#DNA15**	ACGCAGAGTCCGCTCACTGANNNNNCA+T+G
**Adapter1-BC1-vt-#DNA16**	ACGCAGAGTCCGCTCACACANNNNNCA+T+G
**Adapter1-BC1-vt-#DNA17**	ACGCAGAGTCCGCTCAATATNNNNNCA+T+G
**Adapter1-BC1-vt-#DNA18**	ACGCAGAGTCCGCTCTCAAGNNNNNCA+T+G
**Adapter1-BC1-vt-#DNA19**	ACGCAGAGTCCGCTCTTAGGNNNNNCA+T+G
**Adapter1-BC1-vt-#DNA20**	ACGCAGAGTCCGCTCAATGCNNNNNCA+T+G
**Adapter1-BC1-vt-#DNA21**	ACGCAGAGTCCGCTCAGTACNNNNNCA+T+G
**Adapter1-BC1-vt-#DNA22**	ACGCAGAGTCCGCTCCGAATNNNNNCA+T+G
**Adapter1-BC1-vt-#DNA23**	ACGCAGAGTCCGCTCCAACGNNNNNCA+T+G
**Adapter1-BC1-vt-#DNA24**	ACGCAGAGTCCGCTCTAACANNNNNCA+T+G
**Adapter1-BC1-vt-#DNA25**	ACGCAGAGTCCGCTCGAACTNNNNNCA+T+G
**Adapter1-BC1-vt-#DNA26**	ACGCAGAGTCCGCTCATTGGNNNNNCA+T+G
**Adapter1-BC1-vt-#DNA27**	ACGCAGAGTCCGCTCCATCCNNNNNCA+T+G
**Adapter1-BC1-vt-#DNA28**	ACGCAGAGTCCGCTCATAGTNNNNNCA+T+G
**Adapter1-BC1-vt-#DNA29**	ACGCAGAGTCCGCTCTATCTNNNNNCA+T+G
**Adapter1-BC1-vt-#DNA30**	ACGCAGAGTCCGCTCTCTTANNNNNCA+T+G
**Adapter1-BC1-vt-#DNA31**	ACGCAGAGTCCGCTCGTACANNNNNCA+T+G
**Adapter1-BC1-vt-#DNA32**	ACGCAGAGTCCGCTCGTAGCNNNNNCA+T+G
**Adapter1-BC1-vt-#DNA33**	ACGCAGAGTCCGCTCTAAGCNNNNNCA+T+G
**Adapter1-BC1-vt-#DNA34**	ACGCAGAGTCCGCTCATAACNNNNNCA+T+G
**Adapter1-BC1-vt-#DNA35**	ACGCAGAGTCCGCTCCGTAGNNNNNCA+T+G
**Adapter1-BC1-vt-#DNA36**	ACGCAGAGTCCGCTCGATACNNNNNCA+T+G
**Adapter1-BC1-vt-#DNA37**	ACGCAGAGTCCGCTCTGACGNNNNNCA+T+G
**Adapter1-BC1-vt-#DNA38**	ACGCAGAGTCCGCTCGATGTNNNNNCA+T+G
**Adapter1-BC1-vt-#DNA39**	ACGCAGAGTCCGCTCAGTGTNNNNNCA+T+G
**Adapter1-BC1-vt-#DNA40**	ACGCAGAGTCCGCTCAGTCGNNNNNCA+T+G
**Adapter1-BC1-vt-#DNA41**	ACGCAGAGTCCGCTCAATCANNNNNCA+T+G
**Adapter1-BC1-vt-#DNA42**	ACGCAGAGTCCGCTCACTCTNNNNNCA+T+G
**Adapter1-BC1-vt-#DNA43**	ACGCAGAGTCCGCTCAATTGNNNNNCA+T+G
**Adapter1-BC1-vt-#DNA44**	ACGCAGAGTCCGCTCCTATCNNNNNCA+T+G
**Adapter1-BC1-vt-#DNA45**	ACGCAGAGTCCGCTCCGTGANNNNNCA+T+G
**Adapter1-BC1-vt-#DNA46**	ACGCAGAGTCCGCTCTCTACNNNNNCA+T+G
**Adapter1-BC1-vt-#DNA47**	ACGCAGAGTCCGCTCTGTCCNNNNNCA+T+G
**Adapter1-BC1-vt-#DNA48**	ACGCAGAGTCCGCTCGCATANNNNNCA+T+G
**Adapter1-BC1-vt-#DNA49**	ACGCAGAGTCCGCTCACTAGNNNNNCA+T+G
**Adapter1-BC1-vt-#DNA50**	ACGCAGAGTCCGCTCGCACGNNNNNCA+T+G

**Adapter1-BC1-vt-#DNA51**	ACGCAGAGTCCGCTCAGAAGNNNNNCA+T+G
**Adapter1-BC1-vt-#DNA52**	ACGCAGAGTCCGCTCACTTCNNNNNCA+T+G
**Adapter1-BC1-vt-#DNA53**	ACGCAGAGTCCGCTCGATTANNNNNCA+T+G
**Adapter1-BC1-vt-#DNA54**	ACGCAGAGTCCGCTCCGTCTNNNNNCA+T+G
**Adapter1-BC1-vt-#DNA55**	ACGCAGAGTCCGCTCTATTCNNNNNCA+T+G
**Adapter1-BC1-vt-#DNA56**	ACGCAGAGTCCGCTCGTAATNNNNNCA+T+G
**Adapter1-BC1-vt-#DNA57**	ACGCAGAGTCCGCTCATATANNNNNCA+T+G
**Adapter1-BC1-vt-#DNA58**	ACGCAGAGTCCGCTCAGACTNNNNNCA+T+G
**Adapter1-BC1-vt-#DNA59**	ACGCAGAGTCCGCTCATTAANNNNNCA+T+G
**Adapter1-BC1-vt-#DNA60**	ACGCAGAGTCCGCTCTCTCGNNNNNCA+T+G
**Adapter1-BC1-vt-#DNA61**	ACGCAGAGTCCGCTCCGATGNNNNNCA+T+G
**Adapter1-BC1-vt-#DNA62**	ACGCAGAGTCCGCTCGCTAANNNNNCA+T+G
**Adapter1-BC1-vt-#DNA63**	ACGCAGAGTCCGCTCTGTGGNNNNNCA+T+G
**Adapter1-BC1-vt-#DNA64**	ACGCAGAGTCCGCTCGTATGNNNNNCA+T+G
**Adapter1-BC1-vt-#DNA65**	ACGCAGAGTCCGCTCACATGNNNNNCA+T+G
**Adapter1-BC1-vt-#DNA66**	ACGCAGAGTCCGCTCGGATTNNNNNCA+T+G
**Adapter1-BC1-vt-#DNA67**	ACGCAGAGTCCGCTCCAATANNNNNCA+T+G
**Adapter1-BC1-vt-#DNA68**	ACGCAGAGTCCGCTCGCAGTNNNNNCA+T+G
**Adapter1-BC1-vt-#DNA69**	ACGCAGAGTCCGCTCTTACCNNNNNCA+T+G
**Adapter1-BC1-vt-#DNA70**	ACGCAGAGTCCGCTCTCATCNNNNNCA+T+G
**Adapter1-BC1-vt-#DNA71**	ACGCAGAGTCCGCTCAGATCNNNNNCA+T+G
**Adapter1-BC1-vt-#DNA72**	ACGCAGAGTCCGCTCGTTAGNNNNNCA+T+G
**Adapter1-BC1-vt-#DNA73**	ACGCAGAGTCCGCTCGTTGANNNNNCA+T+G
**Adapter1-BC1-vt-#DNA74**	ACGCAGAGTCCGCTCCAAGTNNNNNCA+T+G
**Adapter1-BC1-vt-#DNA75**	ACGCAGAGTCCGCTCCTTGTNNNNNCA+T+G
**Adapter1-BC1-vt-#DNA76**	ACGCAGAGTCCGCTCGAAGANNNNNCA+T+G
**Adapter1-BC1-vt-#DNA77**	ACGCAGAGTCCGCTCTGTAANNNNNCA+T+G
**Adapter1-BC1-vt-#DNA78**	ACGCAGAGTCCGCTCTCACTNNNNNCA+T+G
**Adapter1-BC1-vt-#DNA79**	ACGCAGAGTCCGCTCGCAACNNNNNCA+T+G
**Adapter1-BC1-vt-#DNA80**	ACGCAGAGTCCGCTCACAGCNNNNNCA+T+G
**Adapter1-BC1-vt-#DNA81**	ACGCAGAGTCCGCTCCTACTNNNNNCA+T+G
**Adapter1-BC1-vt-#DNA82**	ACGCAGAGTCCGCTCGAATCNNNNNCA+T+G
**Adapter1-BC1-vt-#DNA83**	ACGCAGAGTCCGCTCAGAGANNNNNCA+T+G
**Adapter1-BC1-vt-#DNA84**	ACGCAGAGTCCGCTCAGTTANNNNNCA+T+G
**Adapter1-BC1-vt-#DNA85**	ACGCAGAGTCCGCTCCATAANNNNNCA+T+G
**Adapter1-BC1-vt-#DNA86**	ACGCAGAGTCCGCTCGGTTGNNNNNCA+T+G
**Adapter1-BC1-vt-#DNA87**	ACGCAGAGTCCGCTCGGTATNNNNNCA+T+G
**Adapter1-BC1-vt-#DNA88**	ACGCAGAGTCCGCTCCTAGANNNNNCA+T+G
**Adapter1-BC1-vt-#DNA89**	ACGCAGAGTCCGCTCATACGNNNNNCA+T+G
**Adapter1-BC1-vt-#DNA90**	ACGCAGAGTCCGCTCCGTTCNNNNNCA+T+G
**Adapter1-BC1-vt-#DNA91**	ACGCAGAGTCCGCTCTGAACNNNNNCA+T+G
**Adapter1-BC1-vt-#DNA92**	ACGCAGAGTCCGCTCGTTCTNNNNNCA+T+G
**Adapter1-BC1-vt-#DNA93**	ACGCAGAGTCCGCTCCTTACNNNNNCA+T+G
**Adapter1-BC1-vt-#DNA94**	ACGCAGAGTCCGCTCTTATTNNNNNCA+T+G
**Adapter1-BC1-vt-#DNA95**	ACGCAGAGTCCGCTCTATGANNNNNCA+T+G
**Adapter1-BC1-vt-#DNA96**	ACGCAGAGTCCGCTCCTAAGNNNNNCA+T+G

Second Split-Pool Barcodes

Oligo name	Sequence
**Adapter2-BC2-Adapter1-#DNA1**	GTCTCGTGGGCTCGGAGATGTGTATAAGAGACAG ATGAAT ACGCAGAGTCCGCTC
**Adapter2-BC2-Adapter1-#DNA2**	GTCTCGTGGGCTCGGAGATGTGTATAAGAGACAG ATATTA ACGCAGAGTCCGCTC
**Adapter2-BC2-Adapter1-#DNA3**	GTCTCGTGGGCTCGGAGATGTGTATAAGAGACAG AATCGA ACGCAGAGTCCGCTC
**Adapter2-BC2-Adapter1-#DNA4**	GTCTCGTGGGCTCGGAGATGTGTATAAGAGACAG CACATA ACGCAGAGTCCGCTC
**Adapter2-BC2-Adapter1-#DNA5**	GTCTCGTGGGCTCGGAGATGTGTATAAGAGACAG GTGATG ACGCAGAGTCCGCTC
**Adapter2-BC2-Adapter1-#DNA6**	GTCTCGTGGGCTCGGAGATGTGTATAAGAGACAG AGGTTT ACGCAGAGTCCGCTC
**Adapter2-BC2-Adapter1-#DNA7**	GTCTCGTGGGCTCGGAGATGTGTATAAGAGACAG ATAACG ACGCAGAGTCCGCTC
**Adapter2-BC2-Adapter1-#DNA8**	GTCTCGTGGGCTCGGAGATGTGTATAAGAGACAG AACGTA ACGCAGAGTCCGCTC
**Adapter2-BC2-Adapter1-#DNA9**	GTCTCGTGGGCTCGGAGATGTGTATAAGAGACAG CAAGCA ACGCAGAGTCCGCTC
**Adapter2-BC2-Adapter1-#DNA10**	GTCTCGTGGGCTCGGAGATGTGTATAAGAGACAG AGGTAC ACGCAGAGTCCGCTC
**Adapter2-BC2-Adapter1-#DNA11**	GTCTCGTGGGCTCGGAGATGTGTATAAGAGACAG GATGGA ACGCAGAGTCCGCTC
**Adapter2-BC2-Adapter1-#DNA12**	GTCTCGTGGGCTCGGAGATGTGTATAAGAGACAG GAGGAA ACGCAGAGTCCGCTC
**Adapter2-BC2-Adapter1-#DNA13**	GTCTCGTGGGCTCGGAGATGTGTATAAGAGACAG CAGATT ACGCAGAGTCCGCTC
**Adapter2-BC2-Adapter1-#DNA14**	GTCTCGTGGGCTCGGAGATGTGTATAAGAGACAG CTAATC ACGCAGAGTCCGCTC
**Adapter2-BC2-Adapter1-#DNA15**	GTCTCGTGGGCTCGGAGATGTGTATAAGAGACAG ATTCCT ACGCAGAGTCCGCTC
**Adapter2-BC2-Adapter1-#DNA16**	GTCTCGTGGGCTCGGAGATGTGTATAAGAGACAG GGTCTA ACGCAGAGTCCGCTC
**Adapter2-BC2-Adapter1-#DNA17**	GTCTCGTGGGCTCGGAGATGTGTATAAGAGACAG GTACTA ACGCAGAGTCCGCTC
**Adapter2-BC2-Adapter1-#DNA18**	GTCTCGTGGGCTCGGAGATGTGTATAAGAGACAG GTTCAC ACGCAGAGTCCGCTC
**Adapter2-BC2-Adapter1-#DNA19**	GTCTCGTGGGCTCGGAGATGTGTATAAGAGACAG CAACAC ACGCAGAGTCCGCTC
**Adapter2-BC2-Adapter1-#DNA20**	GTCTCGTGGGCTCGGAGATGTGTATAAGAGACAG ATAGTT ACGCAGAGTCCGCTC
**Adapter2-BC2-Adapter1-#DNA21**	GTCTCGTGGGCTCGGAGATGTGTATAAGAGACAG AACTAT ACGCAGAGTCCGCTC
**Adapter2-BC2-Adapter1-#DNA22**	GTCTCGTGGGCTCGGAGATGTGTATAAGAGACAG GGGAAA ACGCAGAGTCCGCTC
**Adapter2-BC2-Adapter1-#DNA23**	GTCTCGTGGGCTCGGAGATGTGTATAAGAGACAG GGTTAG ACGCAGAGTCCGCTC
**Adapter2-BC2-Adapter1-#DNA24**	GTCTCGTGGGCTCGGAGATGTGTATAAGAGACAG AGTACA ACGCAGAGTCCGCTC
**Adapter2-BC2-Adapter1-#DNA25**	GTCTCGTGGGCTCGGAGATGTGTATAAGAGACAG ATGGAA ACGCAGAGTCCGCTC
**Adapter2-BC2-Adapter1-#DNA26**	GTCTCGTGGGCTCGGAGATGTGTATAAGAGACAG AGTGTG ACGCAGAGTCCGCTC
**Adapter2-BC2-Adapter1-#DNA27**	GTCTCGTGGGCTCGGAGATGTGTATAAGAGACAG GGTTTC ACGCAGAGTCCGCTC
**Adapter2-BC2-Adapter1-#DNA28**	GTCTCGTGGGCTCGGAGATGTGTATAAGAGACAG CATCAA ACGCAGAGTCCGCTC
**Adapter2-BC2-Adapter1-#DNA29**	GTCTCGTGGGCTCGGAGATGTGTATAAGAGACAG ATCGTG ACGCAGAGTCCGCTC
**Adapter2-BC2-Adapter1-#DNA30**	GTCTCGTGGGCTCGGAGATGTGTATAAGAGACAG CCATAG ACGCAGAGTCCGCTC
**Adapter2-BC2-Adapter1-#DNA31**	GTCTCGTGGGCTCGGAGATGTGTATAAGAGACAG AAACTG ACGCAGAGTCCGCTC
**Adapter2-BC2-Adapter1-#DNA32**	GTCTCGTGGGCTCGGAGATGTGTATAAGAGACAG ATTGTA ACGCAGAGTCCGCTC
**Adapter2-BC2-Adapter1-#DNA33**	GTCTCGTGGGCTCGGAGATGTGTATAAGAGACAG ATGAGG ACGCAGAGTCCGCTC
**Adapter2-BC2-Adapter1-#DNA34**	GTCTCGTGGGCTCGGAGATGTGTATAAGAGACAG CCTAAG ACGCAGAGTCCGCTC
**Adapter2-BC2-Adapter1-#DNA35**	GTCTCGTGGGCTCGGAGATGTGTATAAGAGACAG GTCAGT ACGCAGAGTCCGCTC
**Adapter2-BC2-Adapter1-#DNA36**	GTCTCGTGGGCTCGGAGATGTGTATAAGAGACAG ATCAAG ACGCAGAGTCCGCTC
**Adapter2-BC2-Adapter1-#DNA37**	GTCTCGTGGGCTCGGAGATGTGTATAAGAGACAG ATTGAC ACGCAGAGTCCGCTC
**Adapter2-BC2-Adapter1-#DNA38**	GTCTCGTGGGCTCGGAGATGTGTATAAGAGACAG AAGTCA ACGCAGAGTCCGCTC
**Adapter2-BC2-Adapter1-#DNA39**	GTCTCGTGGGCTCGGAGATGTGTATAAGAGACAG CCTAGT ACGCAGAGTCCGCTC
**Adapter2-BC2-Adapter1-#DNA40**	GTCTCGTGGGCTCGGAGATGTGTATAAGAGACAG CCAATA ACGCAGAGTCCGCTC
**Adapter2-BC2-Adapter1-#DNA41**	GTCTCGTGGGCTCGGAGATGTGTATAAGAGACAG CAAAGT ACGCAGAGTCCGCTC
**Adapter2-BC2-Adapter1-#DNA42**	GTCTCGTGGGCTCGGAGATGTGTATAAGAGACAG AAGCAT ACGCAGAGTCCGCTC
**Adapter2-BC2-Adapter1-#DNA43**	GTCTCGTGGGCTCGGAGATGTGTATAAGAGACAG CTCTCT ACGCAGAGTCCGCTC
**Adapter2-BC2-Adapter1-#DNA44**	GTCTCGTGGGCTCGGAGATGTGTATAAGAGACAG AGCAGT ACGCAGAGTCCGCTC
**Adapter2-BC2-Adapter1-#DNA45**	GTCTCGTGGGCTCGGAGATGTGTATAAGAGACAG ATGGCT ACGCAGAGTCCGCTC
**Adapter2-BC2-Adapter1-#DNA46**	GTCTCGTGGGCTCGGAGATGTGTATAAGAGACAG GAACCA ACGCAGAGTCCGCTC
**Adapter2-BC2-Adapter1-#DNA47**	GTCTCGTGGGCTCGGAGATGTGTATAAGAGACAG AAGATG ACGCAGAGTCCGCTC
**Adapter2-BC2-Adapter1-#DNA48**	GTCTCGTGGGCTCGGAGATGTGTATAAGAGACAG CATAAT ACGCAGAGTCCGCTC
**Adapter2-BC2-Adapter1-#DNA49**	GTCTCGTGGGCTCGGAGATGTGTATAAGAGACAG AGCTAG ACGCAGAGTCCGCTC
**Adapter2-BC2-Adapter1-#DNA50**	GTCTCGTGGGCTCGGAGATGTGTATAAGAGACAG GTTAAG ACGCAGAGTCCGCTC
**Adapter2-BC2-Adapter1-#DNA51**	GTCTCGTGGGCTCGGAGATGTGTATAAGAGACAG CCTGAA ACGCAGAGTCCGCTC
**Adapter2-BC2-Adapter1-#DNA52**	GTCTCGTGGGCTCGGAGATGTGTATAAGAGACAG CTTCCA ACGCAGAGTCCGCTC
**Adapter2-BC2-Adapter1-#DNA53**	GTCTCGTGGGCTCGGAGATGTGTATAAGAGACAG CTCAAT ACGCAGAGTCCGCTC

**Adapter2-BC2-Adapter1-#DNA54**	GTCTCGTGGGCTCGGAGATGTGTATAAGAGACAG GTTTCG ACGCAGAGTCCGCTC
**Adapter2-BC2-Adapter1-#DNA55**	GTCTCGTGGGCTCGGAGATGTGTATAAGAGACAG ATGACA ACGCAGAGTCCGCTC
**Adapter2-BC2-Adapter1-#DNA56**	GTCTCGTGGGCTCGGAGATGTGTATAAGAGACAG AAAGGG ACGCAGAGTCCGCTC
**Adapter2-BC2-Adapter1-#DNA57**	GTCTCGTGGGCTCGGAGATGTGTATAAGAGACAG ATTAGT ACGCAGAGTCCGCTC
**Adapter2-BC2-Adapter1-#DNA58**	GTCTCGTGGGCTCGGAGATGTGTATAAGAGACAG CTAACA ACGCAGAGTCCGCTC
**Adapter2-BC2-Adapter1-#DNA59**	GTCTCGTGGGCTCGGAGATGTGTATAAGAGACAG CCTTTC ACGCAGAGTCCGCTC
**Adapter2-BC2-Adapter1-#DNA60**	GTCTCGTGGGCTCGGAGATGTGTATAAGAGACAG AGTATT ACGCAGAGTCCGCTC
**Adapter2-BC2-Adapter1-#DNA61**	GTCTCGTGGGCTCGGAGATGTGTATAAGAGACAG CAACTT ACGCAGAGTCCGCTC
**Adapter2-BC2-Adapter1-#DNA62**	GTCTCGTGGGCTCGGAGATGTGTATAAGAGACAG AGAGCA ACGCAGAGTCCGCTC
**Adapter2-BC2-Adapter1-#DNA63**	GTCTCGTGGGCTCGGAGATGTGTATAAGAGACAG CTAGTG ACGCAGAGTCCGCTC
**Adapter2-BC2-Adapter1-#DNA64**	GTCTCGTGGGCTCGGAGATGTGTATAAGAGACAG CAGTAA ACGCAGAGTCCGCTC
**Adapter2-BC2-Adapter1-#DNA65**	GTCTCGTGGGCTCGGAGATGTGTATAAGAGACAG AACCTC ACGCAGAGTCCGCTC
**Adapter2-BC2-Adapter1-#DNA66**	GTCTCGTGGGCTCGGAGATGTGTATAAGAGACAG AATTAG ACGCAGAGTCCGCTC
**Adapter2-BC2-Adapter1-#DNA67**	GTCTCGTGGGCTCGGAGATGTGTATAAGAGACAG CATTTG ACGCAGAGTCCGCTC
**Adapter2-BC2-Adapter1-#DNA68**	GTCTCGTGGGCTCGGAGATGTGTATAAGAGACAG GATTAT ACGCAGAGTCCGCTC
**Adapter2-BC2-Adapter1-#DNA69**	GTCTCGTGGGCTCGGAGATGTGTATAAGAGACAG GTGTCT ACGCAGAGTCCGCTC
**Adapter2-BC2-Adapter1-#DNA70**	GTCTCGTGGGCTCGGAGATGTGTATAAGAGACAG GGTTCT ACGCAGAGTCCGCTC
**Adapter2-BC2-Adapter1-#DNA71**	GTCTCGTGGGCTCGGAGATGTGTATAAGAGACAG CTTAGG ACGCAGAGTCCGCTC
**Adapter2-BC2-Adapter1-#DNA72**	GTCTCGTGGGCTCGGAGATGTGTATAAGAGACAG GATATT ACGCAGAGTCCGCTC
**Adapter2-BC2-Adapter1-#DNA73**	GTCTCGTGGGCTCGGAGATGTGTATAAGAGACAG GTATTG ACGCAGAGTCCGCTC
**Adapter2-BC2-Adapter1-#DNA74**	GTCTCGTGGGCTCGGAGATGTGTATAAGAGACAG CATCCT ACGCAGAGTCCGCTC
**Adapter2-BC2-Adapter1-#DNA75**	GTCTCGTGGGCTCGGAGATGTGTATAAGAGACAG GAGTTC ACGCAGAGTCCGCTC
**Adapter2-BC2-Adapter1-#DNA76**	GTCTCGTGGGCTCGGAGATGTGTATAAGAGACAG AATGTC ACGCAGAGTCCGCTC
**Adapter2-BC2-Adapter1-#DNA77**	GTCTCGTGGGCTCGGAGATGTGTATAAGAGACAG CCTTCA ACGCAGAGTCCGCTC
**Adapter2-BC2-Adapter1-#DNA78**	GTCTCGTGGGCTCGGAGATGTGTATAAGAGACAG CATACG ACGCAGAGTCCGCTC
**Adapter2-BC2-Adapter1-#DNA79**	GTCTCGTGGGCTCGGAGATGTGTATAAGAGACAG ATATAG ACGCAGAGTCCGCTC
**Adapter2-BC2-Adapter1-#DNA80**	GTCTCGTGGGCTCGGAGATGTGTATAAGAGACAG GAAACG ACGCAGAGTCCGCTC
**Adapter2-BC2-Adapter1-#DNA81**	GTCTCGTGGGCTCGGAGATGTGTATAAGAGACAG AGAAGG ACGCAGAGTCCGCTC
**Adapter2-BC2-Adapter1-#DNA82**	GTCTCGTGGGCTCGGAGATGTGTATAAGAGACAG AAGGTT ACGCAGAGTCCGCTC
**Adapter2-BC2-Adapter1-#DNA83**	GTCTCGTGGGCTCGGAGATGTGTATAAGAGACAG ATCGCA ACGCAGAGTCCGCTC
**Adapter2-BC2-Adapter1-#DNA84**	GTCTCGTGGGCTCGGAGATGTGTATAAGAGACAG ATCAGC ACGCAGAGTCCGCTC
**Adapter2-BC2-Adapter1-#DNA85**	GTCTCGTGGGCTCGGAGATGTGTATAAGAGACAG GGTAAT ACGCAGAGTCCGCTC
**Adapter2-BC2-Adapter1-#DNA86**	GTCTCGTGGGCTCGGAGATGTGTATAAGAGACAG AGCAAC ACGCAGAGTCCGCTC
**Adapter2-BC2-Adapter1-#DNA87**	GTCTCGTGGGCTCGGAGATGTGTATAAGAGACAG GTCACA ACGCAGAGTCCGCTC
**Adapter2-BC2-Adapter1-#DNA88**	GTCTCGTGGGCTCGGAGATGTGTATAAGAGACAG ATGTGA ACGCAGAGTCCGCTC
**Adapter2-BC2-Adapter1-#DNA89**	GTCTCGTGGGCTCGGAGATGTGTATAAGAGACAG CATAGA ACGCAGAGTCCGCTC
**Adapter2-BC2-Adapter1-#DNA90**	GTCTCGTGGGCTCGGAGATGTGTATAAGAGACAG AACTTG ACGCAGAGTCCGCTC
**Adapter2-BC2-Adapter1-#DNA91**	GTCTCGTGGGCTCGGAGATGTGTATAAGAGACAG CAGAAG ACGCAGAGTCCGCTC
**Adapter2-BC2-Adapter1-#DNA92**	GTCTCGTGGGCTCGGAGATGTGTATAAGAGACAG GGAACT ACGCAGAGTCCGCTC
**Adapter2-BC2-Adapter1-#DNA93**	GTCTCGTGGGCTCGGAGATGTGTATAAGAGACAG AGTTCG ACGCAGAGTCCGCTC
**Adapter2-BC2-Adapter1-#DNA94**	GTCTCGTGGGCTCGGAGATGTGTATAAGAGACAG GAAGGT ACGCAGAGTCCGCTC
**Adapter2-BC2-Adapter1-#DNA95**	GTCTCGTGGGCTCGGAGATGTGTATAAGAGACAG AGAGTC ACGCAGAGTCCGCTC
**Adapter2-BC2-Adapter1-#DNA96**	GTCTCGTGGGCTCGGAGATGTGTATAAGAGACAG ATTCAG ACGCAGAGTCCGCTC

Supplemental Method S2

Single-Cell RNA BAG-seq Laboratory Protocol

Equipment (similar to Drop-seq/DroNc-seq set-up):

Name	Company	Catalog Number
Inverted Microscope	Motic	AE31
Legato 100 Syringe Pumps (3)	KD Scientific	788100
3 mL Syringes	Becton Dickinson	309657
10 mL Syringes	Becton Dickinson	302995
PE-2 Tubing	Scientific Commodities	BB31695-PE/2
26 Gauge Needles	Becton Dickinson	305111
22 Gauge Needles	Becton Dickinson	305155
16 Gauge Needles	Becton Dickinson	305197
PDMS co-flow microfluidic droplet generation device “DroNc-seq device”	Nanoshift, LLC	
40 µm cell strainers	Corning	352340
70 µm cell strainers	Corning	431751

Primers:

Name	Sequence
Linker A2	/5ACryd//iSp18/AAGCAGTGGTATCAACGCAGAGTNNWNNNSTTTTT TTTTTTTTTTTTTTTTTTTTTTTTT
Adapter1-BC1-vt-#RNAx	AGTGGAAAAGGAAGGTGGT <barcode1> NNNNNN rGrGrG (detailed sequences at bottom)
Adapter2-BC2-Adapter1-#RNAx	GACCGACTCGCATTACCCTAT <barcode2> AGTGGAAAAGGAAGGTGG/3InvdT/ (detailed sequences at bottom)
Adapter3-BC3-Adapter2-#RNAx	AAGGCTCTTGACACGAAGGATAG <barcode3>NN GACCGACTCGCATTACCCT*A*T (detailed sequences at bottom)
IS-short primer	AAGCAGTGGTATCAACGCAG
P5-SAM	AATGATACGGCGACCACCGAGATCTACAC CGCTGTCC AAGGCTCTTGACACGAAGGAT*A*G
Read1-SAM	CGCTGTCC AAGGCTCTTGACACGAAGGATAG
Adapter3-BC2a-Adapter1-#RNAx	AAGGCTCTTGACACGAAGGATAG <barcode2a> AGTGGAAAAGGAAGGTG*G*T (detailed sequences at bottom)

Chemical Reagents:

Name	Company	Catalog Number
**Cell Preparation**		
10X PBS	Thermo Fisher Scientific	AM9624
UltraPure BSA	Thermo Fisher Scientific	AM2618
Trypsin-EDTA(0.25%, phenol red)	Thermo Fisher Scientific	25200056
**Aqueous Phase 2**		
Acrylamide/bis-acrylamide, 40% solution	Sigma-Aldrich	A9926
Acrylamide solution, 40%	Sigma-Aldrich	A4058

0.5 M EDTA	Thermo Fisher Scientific	AM9260G
20% Sarkosyl	Sigma-Aldrich	L7414
SUPERase In RNase Inhibitor	Thermo Fisher Scientific	AM2696
0.1M DTT	Thermo Fisher Scientific	707265ML
Ammonium persulfate	Sigma-Aldrich	09913-100G
10% NP-40	Thermo Fisher Scientific	28324
**Oil Phase**		
HFE-7500	Oakwood Chemical	051243
008-Fluorosurfactant	Ran Biotechnologies	008-FluoroSurfactant-5G
TEMED	Bio-Rad	1610801
**Post-Droplet Generation**		
Mineral Oil	Sigma-Aldrich	69794-500ML
20X SSC	Thermo Fisher Scientific	15557044
Perfluorooctanol (PFO)	Sigma-Aldrich	370533
Maxima H Reverse Transcriptase	Thermos Fisher Scientific	EP0753
10 mM dNTP	Sigma-Aldrich	11814362001
Exonuclease I	New England Bio Labs	M0293
Ribonuclease H	Thermo Fisher Scientific	18021-071
Bst 2.0 DNA Polymerase	New England Bio Labs	M0537L
KAPA HiFi HotStart ReadyMix	Roche	KK2602
Agencourt AMPure XP beads	Beckman Coulter	A63880
Nextera XT DNA Library Prep Kit	Illumina	FC-131-1024

Chemical Solutions to Prepare

- PBS-BSA (1X PBS, 0.05% BSA)
- 20% Surfactant in HFE-7500
- 10% Ammonium persulfate
- 6X SSC
- TE-SDS (10 mM Tris pH 8.0, 1 mM EDTA, 0.5% SDS)
- STOP-25 (10 mM Tris pH 8.0, 25 mM EDTA, 0.1% Tween-20, 0.1 M KCl)
- STOP-10 (10 mM Tris pH 8.0, 10 mM EDTA, 0.1% Tween-20, 0.1 M KCl)
- STOP-1 (10 mM Tris pH 8.0, 1 mM EDTA, 0.1% Tween-20, 0.1 M KCl)
- 1X Exonuclease Reaction Buffer
- HBW (10 mM Tris pH 8.0, 1 mM EDTA, 0.1% Tween-20)

scRNA BAG-seq Experiment

Cell Solution Preparation

1. Warm trypsin, 1X PBS and tissue culture media in a 37°C water bath.
2. Prepare PBS-BSA solution on ice.
3. Retrieve adherent cells from incubator and discard tissue culture media from plates.
4. Add 8 mL 1X PBS to each plate. Tilt plates back and forth to wash adherent cells. Then, discard PBS.
5. Trypsinize cells for 5 minutes with 1 mL warm Trypsin-EDTA. Then, add 6 mL tissue culture media to plate and pipet up and down multiple times. Collect solution in a 15 mL conical tube.
6. Centrifuge cells 300 rcf for 5 minutes. Discard supernatant.
7. Resuspend pellet with 8 mL 1X PBS. Centrifuge cells 300 rcf for 5 minutes. Discard supernatant.
8. Resuspend pellet with 1.5 mL PBS-BSA and filter cell solution through a 40 μm strainer.
9. Count cells.
10. Prepare a 1 mL solution of cells with a concentration of 2.5E5 cells/mL.

Oil Phase Preparation

1. Combine the following; vortex to mix (*enough for 2 samples*):

600 μL	20% surfactant
9.6 μL	TEMED
1.8 mL	HFE7500

Aqueous Phase 2 Preparation

1. Combine the following; pipet to mix (*enough for 2 samples*):

Aqueous Phase 2 Solution	Volume (μL)	Final Concentration
AA/bis-acrylamide, 40%	180	
Acrylamide solution, 40%	129	
H_2O	191	
500 μM LinkerA2 primer	80	20 μM
0.5 M EDTA	50	12.5 mM
1 M Tris pH 7.5	70	35 mM
20 % Sarkosyl	10	0.1%
SUPERase In RNase Inhibitor	100	1 U/μL
0.1 M DTT	30	1.5 mM
10% NP-40	100	0.5%
10% Ammonium persulfate (APS)	60	0.3%
**Total**	**1 mL**	

Microfluidics Setup & Oil Droplet Collection

1. Turn on microscope. Place microfluidic device on microscope stage.
2. Insert a piece of PE/2 tubing into the output well and place the other end into a waste container (15 mL conical tube).
3. Use the following syringes to collect the three solutions.

    a. **Oil Phase:** Use a 10 mL syringe with a 16 G needle to aspirate solution into the syringe.
    b. **Cell Solution or Aqueous Phase 2**: Use a 3 mL syringe and a 22 G needle to aspirate solution into the syringe.
4. For each syringe, change the needle to a 26 G needle and insert into PE/2 tubing.
5. Place syringes into their designated syringe pumps and insert tubing into the corresponding wells of the microfluidics chamber
    a. From left to right: Oil – Aqueous 2 – Cell Solution (Aqueous 1) – Output
6. Once all syringes and tubing are in place, set the following flow rates:
    a. **Oil Phase:** 3,000 μL/hr
    b. **Cell Solution:** 750 μL/hr
    c. **Aqueous Phase 2:** 750 μL/hr
7. Begin by first turning on the cell solution pump.
8. Once cell solution is flowing, turn on the aqueous phase 2 pump.
9. Once the interphase between cell solution and aqueous phase two has stabilized, turn on the oil phase pump and wait for the droplet formation to stabilize.
10. Once the flow has stabilized, place the output tubing on a glass slide under the microscope to assess the size uniformity of oil droplets.
11. Once droplet size is uniform, collect droplets in 300 μL of mineral oil in 1.5 mL tubes.
12. After collection, incubate BAGs at room temperature for 2.5 hours.
13. Incubate BAGs at 50°C for 5 minutes, then incubate at room temperature for 10 minutes.

Droplet Breakage

1. Remove mineral oil in the top layer using pipet. Remove oil in the bottom layer using a 3 mL syringe and 22G needle.
2. Add 600 μL 6X SSC buffer and 150 μL perfluorooctanol (PFO) in a fume hood. Shake the tube by hand to break the droplets and release balls of acrylamide gel (BAGs).
3. Centrifuge 1000 rcf for 1.5 minutes.
4. Discard the top and bottom layer, leaving the translucent BAG layer in the tube.
5. Add 600 μL 6x SSC and shake tube multiple times. Centrifuge 1000 rcf for 1.5 minutes. Discard the top layer and any visible PFO from the bottom of the tube.

Reverse Transcription / First Split-Pool

1. Wash the BAG layer from previous step with the following RT wash solution.
    a. **RT wash solution**: 850 μL 5x Reverse Transcriptase Buffer, 25 μL 0.1M DTT, and 25 μL SUPERase In RNase inhibitor
    b. Pipet up and down, centrifuge 1000 rcf for 1.5 minutes, discard the supernatant.
2. Prepare the following BAG solution; pipet up and down to mix:

**BAG Solution**	**Volume (μL)**
5x RT Buffer	250
SUPERase In RNase Inhibitor	15
0.1 M DTT	15
BAGs	200
**Total**	**480**

    a. Transfer 60 μL BAG solution to each tube of 8-well PCR strip.
    b. Quickly transfer 5 μL into each tube of a 96-well plate using a multipipette.
3. Prepare the following reverse transcription master mix in a 5 ml tube; vortex to mix:

Reverse Transcription Master Mix	Volume (µL)
H_2O	1048
10 mM dNTP	200
0.1 M DTT	100
SUPERase In RNase Inhibitor	52
Maxima H- Reverse Transcriptase	100
**Total**	**1.5 mL**

    a. Transfer 185 µL Reverse Transcription Master Mix into each well of an 8-well PCR strip.
    b. Transfer 13.5 µL into each well of the 96-well plate.

4. Add 2 µL of well-specific 50 µM Template-Switch Oligos (“Adapter1-BC1-vt-#RNAx”) into each corresponding well. Each well should have a unique well-specific primer.
5. Incubate with rotation at room temperature for 30 minutes.
6. Incubate with rotation at 42°C for 1 hour, followed by 50°C for 1 hour.
7. Stop the reaction with the following steps:
    a. Add 100 µL TE-SDS to each reaction tube of the plate. Centrifuge plates 1000 rcf for 2 minutes at room temperature.
    b. Without disturbing the BAG pellet, discard 85 µL from **the top** of each tube.
    c. Add 5 ml TE-SDS into solution basin. Transfer the remaining BAG solutions from the plate into the solution basin (~35 µL per tube).
    d. Transfer BAG solution from solution basin into a 15 mL conical tube. Centrifuge 300 rcf for 4 minutes. Discard supernatant.
    e. Add 800 µL STOP-10, pipet up and down, and transfer BAG solution into a 1.5 mL tube. Centrifuge 1000 rcf for 1.5 minutes. Discard supernatant.
    f. Continue to exonuclease treatment or re-suspend in 800 µL STOP-10 and store at 4°C.

--------------------Safe Stopping Point: Store BAGs in STOP-10 at 4°C overnight--------------------

Exonuclease Treatment

1. If stored in STOP-10, centrifuge 1000 rcf for 1.5 minutes and discard supernatant.
2. Resuspend in STOP-1 for a wash. Centrifuge 1000 rcf for 1.5 minutes. Discard supernatant.
3. Resuspend in 1X Exonuclease I Reaction Buffer for a wash. Centrifuge 1000 rcf for 1.5 minutes. Discard supernatant.
4. Prepare the following exonuclease treatment solution; vortex to mix:

Exonuclease Treatment Solution	Volume (µL)
H_2O	665
10X Exonuclease I Reaction buffer	80
Exonuclease I enzyme	40
Ribonuclease H	15
**Total**	**800 µL**

5. Transfer 800 µL Exonuclease Treatment Solution to the tube of BAGs; pipet to mix.
6. Incubate tubes with rotation at 37°C for 1 hour.
7. Centrifuge 1000 rcf for 1 minute. Discard supernatant.
8. Stop the reaction with the following washes:

   a. Resuspend in 800 µL STOP-25. Centrifuge 1000 rcf for 1.5 minutes. Discard supernatant. Repeat for a total of two STOP-25 washes.
   b. Use the above wash method for one STOP-10 wash.

--------------------Safe Stopping Point: Store BAGs in STOP-10 at 4°C overnight--------------------

Second Split-Pool

0. Note: If one desires to do 2 cycles of split-pool in total, skip this section and go ahead to the next section "Third Split-Pool" and use primers "Adapter3-BC2a-Adapter1-#RNAx"
1. Preparation:
   a. If stored in STOP-10 overnight, centrifuge 1000 rcf for 1.5 minutes. Discard supernatant.
   b. Resuspend in 800 µL HBW for a wash. Centrifuge 1000 rcf for 1.5 minutes. Discard supernatant. Resuspend BAGs in 200 µL HBW.
2. Denaturation
   a. Prepare fresh Denaturation Solution: (Adapted from (Zilionis et al. 2017))
      i. 9.7 mL H_2O + 150 µL 10 M NaOH + 150 µL 30% (wt/wt) Brij-35
   b. Add 1 mL Denaturation Solution to BAGs. Pipet up and down 5 times. Incubate 10 minutes at room temperature on rotator. Centrifuge 400 rcf for 2 minutes at room temperature. Discard supernatant.
   c. Wash BAGs twice with Denaturation Solution
      i. Add 1 mL Denaturation Solution to BAGs. Pipet up and down 5 times. Incubate 3 minutes at room temperature on rotator. Centrifuge 400 rcf for 2 minutes at room temperature. Discard supernatant.
3. Neutralization
   a. Prepare Neutralization Solution
      i. 7.7 mL H_2O + 1 mL 1 M Tris (pH 8.0) + 1 mL 1 M NaCl + 200 µL 0.5 M EDTA + 100 µL 10% (vol/vol) Tween-20
   b. Wash BAGs twice with Neutralization Buffer
      i. Add 1 mL Neutralization Solution to BAGs. Pipet up and down 5 times. Incubate 3 minutes at room temperature on rotator. Centrifuge 400 rcf for 2 minutes at room temperature. Discard supernatant.
   c. Wash BAGs once with HBW
      i. Add 1 mL HBW to BAGs. Pipet up and down 5 times. Centrifuge 1000 rcf for 2 minutes. Discard supernatant.
   d. Re-suspend BAGs in HBW (400 µL total)
4. Preset thermocycler to 75°C and incubator to 57°C.
5. Prepare the following Isothermal Amplification Buffer (IAB) Mix; vortex to mix:

**IAB Mix**	**Volume (µL)** for one 96-well plate	*Volume (µL) per well*
H_2O	1150.4	*~12*
10x IABuffer	201.6	*2.1*
10 mM dNTP	144	*1.5*
BAGs in HBW	400	*~4.2*
**Total:**	**1896 µL**	*19.75 µL*

   a. Transfer 237 µL IAB mix to each tube of 8-well PCR strip.

   b. Quickly transfer 19.75 µL into each tube of a 96-well plate using a multipipette.
6. Add 1.25 µL of the 96 different 100 µM “Adapter2-BC2-Adapter1-#RNAx” primers into each corresponding well. Each well should have a unique primer.
7. Invert plate for 2 minutes. Then incubate the plate at 75°C for 5 minutes.
8. Incubate plate at 57°C with rotation for 20 minutes.
9. Prepare the following BST Master Mix on ice; pipet to mix:

**BST Master Mix**	**Volume (µL)** for one 96-well plate	*Volume (µL) per well*
H_2O	310	*3.1*
10x IABuffer	40	*0.4*
Bst2.0	50	*0.5*
**Total:**	**400 µL**	*4 µL*

   a. Transfer 50 µL Bst Mix to each tube of 8-well PCR strip.
   b. Transfer 4 µL into each tube of a 96-well plate using a multipipette. Pipet to mix.
10. Incubate at 57°C with rotation for 1 hr.
11. Stop the reaction with the following washes:
   a. Add 100 µL STOP-25 to each well of the plate. Centrifuge plates 1000 rcf for 2 minutes at room temperature.
   b. Without disturbing the BAG pellet, discard 85 µL from the top of each tube.
   a. Add 5 mL STOP-25 into solution basin. Transfer the remaining BAG solutions from the plate into the solution basin (40 µL per tube).
   b. Transfer BAG solution from solution basin into a 15 mL conical. Centrifuge 300 rcf for 4 minutes. Discard supernatant.
   c. Add 800 µL STOP-10, pipet up and down, and transfer BAG solution into a 1.5 mL tube. Centrifuge 1000 rcf for 1.5 minutes. Discard supernatant.
12. Continue to third split-pool or resuspend BAGs in 800 µL STOP-10 and store at 4°C.

--------------------Safe Stopping Point: Store BAGs in STOP-10 at 4°C overnight--------------------

Third Split-Pool

1. If stored in STOP-10 overnight, centrifuge 1000 rcf for 1.5 minutes. Discard supernatant.
2. Resuspend in 800 µL HBW for a wash. Centrifuge 1000 rcf for 1.5 minutes. Discard supernatant. Resuspend BAGs in 500 µL HBW.
3. Use a microscope to count the number of BAGs per µL.
4. Prepare the following BAG mix; pipet to mix:

**BAG Mix**	**Volume (µL)**
BAGs in HBW	x
H_2O	720 - x
Total	720 µL

   a. Transfer 90 µL BAG mix to each tube of 8-well PCR strip.
   b. Quickly transfer 7.5 µL into each tube of a 96-well plate using a multipipette.
5. Add 1.5 µL of 96 different 10 µM “Adapter3-BC3-Adapter2-#RNAx” primers into each corresponding well. Each well should have a unique primer.
6. On ice, add 10 µL KAPA HiFi HotStart ReadyMix to each tube of 96-well plate. Pipet to mix.
7. Place on thermal cycler for the following program:

**<u>RNA: Linear Extension</u>**
95°C 20 s
**8 cycles of:**
98°C 30 s
60°C 1 min
72°C 3 min
**Then:**
72°C 3 min
4°C infinite

8. On ice, add 1 µL of 10 µM “IS-short” primer. Use another set of tips to pipet to mix.
9. Place on thermal cycler for the following program:

**<u>RNA: Exponential amplification</u>**
95°C 2 minutes
**4 cycles of:**
98°C 20 s
60°C 1 min
72°C 3 min
**6 cycles of:**
98°C 20 s
62°C 20 s
72°C 3 min
**Then:**
72°C 5 min
4°C infinite

10. Purify sample twice with AMPure XP beads using the following guidelines:
    a. First Pool: Pool 10 ul from each of the eight tubes in each column into a new PCR tube.

(ex. 1A-1H) into a new PCR tube, as shown.

1A	2	3	4	5	6	7	8	9	10	11	12
B											
C											
D											
E											
F											
G											
H											

1	2	3	4	5	6	7	8	9	10	11	12

(After pool: 80 µL each)

    b. First Purification: Purify these 12 samples with 0.7x ratio (56 µL) of AMPure XP beads, and elute with 15 µL of H_2O using AMPure XP protocol.
    c. Second Pool

Pool 14 ul from six tubes into one tube.

1	2	3	4	5	6	7	8	9	10	11	12

1	2

(After pool: 84 µL each)

d. Second Purification: Purify these two samples with another 0.7x ratio (59 µL) of AMPure XP beads, and elute with 15 µL H_2O using AMPure XP protocol.

11. Run a High Sensitivity DNA Chip on an Agilent Bioanalyzer.

Tagmentation of cDNA and Making Final Sequencing Library

1. Using Bioanalyzer data, combine 1.5 ng of cDNA with H_2O in a total volume of 5 µL. Add 10 µL of Nextera TD buffer
2. On ice, add 5 µL of Amplicon Tagment enzyme. Pipet to mix.
3. Incubate sample at 55°C for 4 minutes.
4. Quickly add 5 µL of Neutralization Buffer. Pipet to mix.
5. Incubate at room temperature for 5 minutes
6. On ice, combine the following; pipet to mix:

**Final PCR Reaction Mix**	**Volume (in µL)**
H_2O	8
10 µM Nextera N70x oligo	1
10 µM "P5-SAM" primer	1
Nextera PCR mix	15
**Total**	**25 µL**

12. Place on thermal cycler for the following program:

**Final PCR gel**
95°C 30s
**12 cycles of:**
95°C 10 s
55°C 30 s
72°C 30 s
**Then:**
72°C 5 min
4°C infinite

13. Purify sample with 0.8x ratio (40 µL) of Ampure XP Beads, and elute with 15 µL H_2O using AMPure XP protocol.
14. Run a High Sensitivity DNA Chip on an Agilent Bioanalyzer to check final concentration of sample.
15. The library should have an average bp size of 400-500bp.
16. Sequence your sample using Illumina platform with custom Read1 sequencing primer "Read1-SAM".

Barcode Sequences

First Split-Pool Barcodes

Oligo name	Sequence
Adapter1-BC1-vt-#RNA1	AGTGGAAAAGGAAGGTGGT TATTAT NNNNNN rGrGrG
Adapter1-BC1-vt-#RNA2	AGTGGAAAAGGAAGGTGGT CTATGG NNNNNN rGrGrG
Adapter1-BC1-vt-#RNA3	AGTGGAAAAGGAAGGTGGT AAAACG NNNNNN rGrGrG
Adapter1-BC1-vt-#RNA4	AGTGGAAAAGGAAGGTGGT CAAACA NNNNNN rGrGrG
Adapter1-BC1-vt-#RNA5	AGTGGAAAAGGAAGGTGGT TATCGT NNNNNN rGrGrG
Adapter1-BC1-vt-#RNA6	AGTGGAAAAGGAAGGTGGT ACTAAG NNNNNN rGrGrG
Adapter1-BC1-vt-#RNA7	AGTGGAAAAGGAAGGTGGT GCATCT NNNNNN rGrGrG
Adapter1-BC1-vt-#RNA8	AGTGGAAAAGGAAGGTGGT AGTTTG NNNNNN rGrGrG
Adapter1-BC1-vt-#RNA9	AGTGGAAAAGGAAGGTGGT CGATTT NNNNNN rGrGrG
Adapter1-BC1-vt-#RNA10	AGTGGAAAAGGAAGGTGGT ATCAGC NNNNNN rGrGrG
Adapter1-BC1-vt-#RNA11	AGTGGAAAAGGAAGGTGGT AACAAG NNNNNN rGrGrG
Adapter1-BC1-vt-#RNA12	AGTGGAAAAGGAAGGTGGT GAAGTT NNNNNN rGrGrG
Adapter1-BC1-vt-#RNA13	AGTGGAAAAGGAAGGTGGT GGTCTA NNNNNN rGrGrG
Adapter1-BC1-vt-#RNA14	AGTGGAAAAGGAAGGTGGT GACTAT NNNNNN rGrGrG
Adapter1-BC1-vt-#RNA15	AGTGGAAAAGGAAGGTGGT GTATCA NNNNNN rGrGrG
Adapter1-BC1-vt-#RNA16	AGTGGAAAAGGAAGGTGGT TGCATA NNNNNN rGrGrG
Adapter1-BC1-vt-#RNA17	AGTGGAAAAGGAAGGTGGT TAATCA NNNNNN rGrGrG
Adapter1-BC1-vt-#RNA18	AGTGGAAAAGGAAGGTGGT TGGTAT NNNNNN rGrGrG
Adapter1-BC1-vt-#RNA19	AGTGGAAAAGGAAGGTGGT ATGTGT NNNNNN rGrGrG
Adapter1-BC1-vt-#RNA20	AGTGGAAAAGGAAGGTGGT TACTGG NNNNNN rGrGrG
Adapter1-BC1-vt-#RNA21	AGTGGAAAAGGAAGGTGGT AGCTGA NNNNNN rGrGrG
Adapter1-BC1-vt-#RNA22	AGTGGAAAAGGAAGGTGGT TAAATG NNNNNN rGrGrG
Adapter1-BC1-vt-#RNA23	AGTGGAAAAGGAAGGTGGT CTGAAC NNNNNN rGrGrG
Adapter1-BC1-vt-#RNA24	AGTGGAAAAGGAAGGTGGT TAATGC NNNNNN rGrGrG
Adapter1-BC1-vt-#RNA25	AGTGGAAAAGGAAGGTGGT TAACTA NNNNNN rGrGrG
Adapter1-BC1-vt-#RNA26	AGTGGAAAAGGAAGGTGGT CGAAAA NNNNNN rGrGrG
Adapter1-BC1-vt-#RNA27	AGTGGAAAAGGAAGGTGGT ACGAAT NNNNNN rGrGrG
Adapter1-BC1-vt-#RNA28	AGTGGAAAAGGAAGGTGGT ATGACG NNNNNN rGrGrG
Adapter1-BC1-vt-#RNA29	AGTGGAAAAGGAAGGTGGT AGGAGT NNNNNN rGrGrG
Adapter1-BC1-vt-#RNA30	AGTGGAAAAGGAAGGTGGT CTAGAC NNNNNN rGrGrG
Adapter1-BC1-vt-#RNA31	AGTGGAAAAGGAAGGTGGT TAGATT NNNNNN rGrGrG
Adapter1-BC1-vt-#RNA32	AGTGGAAAAGGAAGGTGGT TATGTC NNNNNN rGrGrG
Adapter1-BC1-vt-#RNA33	AGTGGAAAAGGAAGGTGGT CGGATA NNNNNN rGrGrG
Adapter1-BC1-vt-#RNA34	AGTGGAAAAGGAAGGTGGT ACGTTT NNNNNN rGrGrG
Adapter1-BC1-vt-#RNA35	AGTGGAAAAGGAAGGTGGT ACTTGT NNNNNN rGrGrG
Adapter1-BC1-vt-#RNA36	AGTGGAAAAGGAAGGTGGT GAGTAC NNNNNN rGrGrG
Adapter1-BC1-vt-#RNA37	AGTGGAAAAGGAAGGTGGT ATCTAA NNNNNN rGrGrG
Adapter1-BC1-vt-#RNA38	AGTGGAAAAGGAAGGTGGT GTTTGC NNNNNN rGrGrG
Adapter1-BC1-vt-#RNA39	AGTGGAAAAGGAAGGTGGT AGGATG NNNNNN rGrGrG
Adapter1-BC1-vt-#RNA40	AGTGGAAAAGGAAGGTGGT CTAGCA NNNNNN rGrGrG
Adapter1-BC1-vt-#RNA41	AGTGGAAAAGGAAGGTGGT AAACGT NNNNNN rGrGrG
Adapter1-BC1-vt-#RNA42	AGTGGAAAAGGAAGGTGGT GGTCAT NNNNNN rGrGrG
Adapter1-BC1-vt-#RNA43	AGTGGAAAAGGAAGGTGGT TCAAAG NNNNNN rGrGrG
Adapter1-BC1-vt-#RNA44	AGTGGAAAAGGAAGGTGGT AAAGCA NNNNNN rGrGrG
Adapter1-BC1-vt-#RNA45	AGTGGAAAAGGAAGGTGGT AGTGAC NNNNNN rGrGrG
Adapter1-BC1-vt-#RNA46	AGTGGAAAAGGAAGGTGGT GTAGTC NNNNNN rGrGrG
Adapter1-BC1-vt-#RNA47	AGTGGAAAAGGAAGGTGGT CATGAG NNNNNN rGrGrG
Adapter1-BC1-vt-#RNA48	AGTGGAAAAGGAAGGTGGT GACTTG NNNNNN rGrGrG
Adapter1-BC1-vt-#RNA49	AGTGGAAAAGGAAGGTGGT ACAATC NNNNNN rGrGrG
Adapter1-BC1-vt-#RNA50	AGTGGAAAAGGAAGGTGGT ATTATG NNNNNN rGrGrG
Adapter1-BC1-vt-#RNA51	AGTGGAAAAGGAAGGTGGT GGATTG NNNNNN rGrGrG

**Adapter1-BC1-vt-#RNA52**	AGTGGAAAAGGAAGGTGGT TAGGCA NNNNNN rGrGrG
**Adapter1-BC1-vt-#RNA53**	AGTGGAAAAGGAAGGTGGT TACGTG NNNNNN rGrGrG
**Adapter1-BC1-vt-#RNA54**	AGTGGAAAAGGAAGGTGGT ATAGGT NNNNNN rGrGrG
**Adapter1-BC1-vt-#RNA55**	AGTGGAAAAGGAAGGTGGT TTAGAT NNNNNN rGrGrG
**Adapter1-BC1-vt-#RNA56**	AGTGGAAAAGGAAGGTGGT TCGGAT NNNNNN rGrGrG
**Adapter1-BC1-vt-#RNA57**	AGTGGAAAAGGAAGGTGGT TCTAGC NNNNNN rGrGrG
**Adapter1-BC1-vt-#RNA58**	AGTGGAAAAGGAAGGTGGT TCTGAC NNNNNN rGrGrG
**Adapter1-BC1-vt-#RNA59**	AGTGGAAAAGGAAGGTGGT GGAAAC NNNNNN rGrGrG
**Adapter1-BC1-vt-#RNA60**	AGTGGAAAAGGAAGGTGGT GAGGAT NNNNNN rGrGrG
**Adapter1-BC1-vt-#RNA61**	AGTGGAAAAGGAAGGTGGT CAATTC NNNNNN rGrGrG
**Adapter1-BC1-vt-#RNA62**	AGTGGAAAAGGAAGGTGGT TCGTTA NNNNNN rGrGrG
**Adapter1-BC1-vt-#RNA63**	AGTGGAAAAGGAAGGTGGT AGGACA NNNNNN rGrGrG
**Adapter1-BC1-vt-#RNA64**	AGTGGAAAAGGAAGGTGGT TACAGT NNNNNN rGrGrG
**Adapter1-BC1-vt-#RNA65**	AGTGGAAAAGGAAGGTGGT TTACGG NNNNNN rGrGrG
**Adapter1-BC1-vt-#RNA66**	AGTGGAAAAGGAAGGTGGT TACGGA NNNNNN rGrGrG
**Adapter1-BC1-vt-#RNA67**	AGTGGAAAAGGAAGGTGGT TATACT NNNNNN rGrGrG
**Adapter1-BC1-vt-#RNA68**	AGTGGAAAAGGAAGGTGGT GTAAGG NNNNNN rGrGrG
**Adapter1-BC1-vt-#RNA69**	AGTGGAAAAGGAAGGTGGT AGTAGA NNNNNN rGrGrG
**Adapter1-BC1-vt-#RNA70**	AGTGGAAAAGGAAGGTGGT TTCGTA NNNNNN rGrGrG
**Adapter1-BC1-vt-#RNA71**	AGTGGAAAAGGAAGGTGGT AATAGG NNNNNN rGrGrG
**Adapter1-BC1-vt-#RNA72**	AGTGGAAAAGGAAGGTGGT CTAGTT NNNNNN rGrGrG
**Adapter1-BC1-vt-#RNA73**	AGTGGAAAAGGAAGGTGGT AAGTCG NNNNNN rGrGrG
**Adapter1-BC1-vt-#RNA74**	AGTGGAAAAGGAAGGTGGT CTAAGT NNNNNN rGrGrG
**Adapter1-BC1-vt-#RNA75**	AGTGGAAAAGGAAGGTGGT CAGAGT NNNNNN rGrGrG
**Adapter1-BC1-vt-#RNA76**	AGTGGAAAAGGAAGGTGGT TAGGGT NNNNNN rGrGrG
**Adapter1-BC1-vt-#RNA77**	AGTGGAAAAGGAAGGTGGT GAACAT NNNNNN rGrGrG
**Adapter1-BC1-vt-#RNA78**	AGTGGAAAAGGAAGGTGGT CTTGGT NNNNNN rGrGrG
**Adapter1-BC1-vt-#RNA79**	AGTGGAAAAGGAAGGTGGT ATTCGT NNNNNN rGrGrG
**Adapter1-BC1-vt-#RNA80**	AGTGGAAAAGGAAGGTGGT AGGTTC NNNNNN rGrGrG
**Adapter1-BC1-vt-#RNA81**	AGTGGAAAAGGAAGGTGGT GAATGG NNNNNN rGrGrG
**Adapter1-BC1-vt-#RNA82**	AGTGGAAAAGGAAGGTGGT CAAAAG NNNNNN rGrGrG
**Adapter1-BC1-vt-#RNA83**	AGTGGAAAAGGAAGGTGGT AATGCG NNNNNN rGrGrG
**Adapter1-BC1-vt-#RNA84**	AGTGGAAAAGGAAGGTGGT TTCAAT NNNNNN rGrGrG
**Adapter1-BC1-vt-#RNA85**	AGTGGAAAAGGAAGGTGGT GTTAAA NNNNNN rGrGrG
**Adapter1-BC1-vt-#RNA86**	AGTGGAAAAGGAAGGTGGT TAGTAA NNNNNN rGrGrG
**Adapter1-BC1-vt-#RNA87**	AGTGGAAAAGGAAGGTGGT ATTGTT NNNNNN rGrGrG
**Adapter1-BC1-vt-#RNA88**	AGTGGAAAAGGAAGGTGGT TCATTC NNNNNN rGrGrG
**Adapter1-BC1-vt-#RNA89**	AGTGGAAAAGGAAGGTGGT ATATAC NNNNNN rGrGrG
**Adapter1-BC1-vt-#RNA90**	AGTGGAAAAGGAAGGTGGT CAACGA NNNNNN rGrGrG
**Adapter1-BC1-vt-#RNA91**	AGTGGAAAAGGAAGGTGGT AATTCT NNNNNN rGrGrG
**Adapter1-BC1-vt-#RNA92**	AGTGGAAAAGGAAGGTGGT AAGAAC NNNNNN rGrGrG
**Adapter1-BC1-vt-#RNA93**	AGTGGAAAAGGAAGGTGGT TGTTGC NNNNNN rGrGrG
**Adapter1-BC1-vt-#RNA94**	AGTGGAAAAGGAAGGTGGT AACAGA NNNNNN rGrGrG
**Adapter1-BC1-vt-#RNA95**	AGTGGAAAAGGAAGGTGGT GGAATA NNNNNN rGrGrG
**Adapter1-BC1-vt-#RNA96**	AGTGGAAAAGGAAGGTGGT ATATTA NNNNNN rGrGrG

Second Split-Pool Barcodes

Oligo name	Sequence
**Adapter2-BC2-Adapter1-#RNA1**	GACCGACTCGCATTACCCTATTCACCAAGTGGAAAAGGAAGGTGG/3InvdT/
**Adapter2-BC2-Adapter1-#RNA2**	GACCGACTCGCATTACCCTATCCAGTAAGTGGAAAAGGAAGGTGG/3InvdT/
**Adapter2-BC2-Adapter1-#RNA3**	GACCGACTCGCATTACCCTATTCCTAAAGTGGAAAAGGAAGGTGG/3InvdT/
**Adapter2-BC2-Adapter1-#RNA4**	GACCGACTCGCATTACCCTATCCATTCAGTGGAAAAGGAAGGTGG/3InvdT/
**Adapter2-BC2-Adapter1-#RNA5**	GACCGACTCGCATTACCCTATTCAACCAGTGGAAAAGGAAGGTGG/3InvdT/
**Adapter2-BC2-Adapter1-#RNA6**	GACCGACTCGCATTACCCTATCTGCAAAGTGGAAAAGGAAGGTGG/3InvdT/
**Adapter2-BC2-Adapter1-#RNA7**	GACCGACTCGCATTACCCTATGAAATAAGTGGAAAAGGAAGGTGG/3InvdT/
**Adapter2-BC2-Adapter1-#RNA8**	GACCGACTCGCATTACCCTATAGGTATAGTGGAAAAGGAAGGTGG/3InvdT/
**Adapter2-BC2-Adapter1-#RNA9**	GACCGACTCGCATTACCCTATACTCTAAGTGGAAAAGGAAGGTGG/3InvdT/
**Adapter2-BC2-Adapter1-#RNA10**	GACCGACTCGCATTACCCTATTCCCATAGTGGAAAAGGAAGGTGG/3InvdT/
**Adapter2-BC2-Adapter1-#RNA11**	GACCGACTCGCATTACCCTATGGTTTTAGTGGAAAAGGAAGGTGG/3InvdT/
**Adapter2-BC2-Adapter1-#RNA12**	GACCGACTCGCATTACCCTATTCTGTAAGTGGAAAAGGAAGGTGG/3InvdT/
**Adapter2-BC2-Adapter1-#RNA13**	GACCGACTCGCATTACCCTATAGTCACAGTGGAAAAGGAAGGTGG/3InvdT/
**Adapter2-BC2-Adapter1-#RNA14**	GACCGACTCGCATTACCCTATAACACAAGTGGAAAAGGAAGGTGG/3InvdT/
**Adapter2-BC2-Adapter1-#RNA15**	GACCGACTCGCATTACCCTATTGTATTAGTGGAAAAGGAAGGTGG/3InvdT/
**Adapter2-BC2-Adapter1-#RNA16**	GACCGACTCGCATTACCCTATTTACAAAGTGGAAAAGGAAGGTGG/3InvdT/
**Adapter2-BC2-Adapter1-#RNA17**	GACCGACTCGCATTACCCTATATATTCAGTGGAAAAGGAAGGTGG/3InvdT/
**Adapter2-BC2-Adapter1-#RNA18**	GACCGACTCGCATTACCCTATGCCTATAGTGGAAAAGGAAGGTGG/3InvdT/
**Adapter2-BC2-Adapter1-#RNA19**	GACCGACTCGCATTACCCTATAGCGAAAGTGGAAAAGGAAGGTGG/3InvdT/
**Adapter2-BC2-Adapter1-#RNA20**	GACCGACTCGCATTACCCTATAAACCTAGTGGAAAAGGAAGGTGG/3InvdT/
**Adapter2-BC2-Adapter1-#RNA21**	GACCGACTCGCATTACCCTATAACAACAGTGGAAAAGGAAGGTGG/3InvdT/
**Adapter2-BC2-Adapter1-#RNA22**	GACCGACTCGCATTACCCTATCTTATTAGTGGAAAAGGAAGGTGG/3InvdT/
**Adapter2-BC2-Adapter1-#RNA23**	GACCGACTCGCATTACCCTATGACTGAAGTGGAAAAGGAAGGTGG/3InvdT/
**Adapter2-BC2-Adapter1-#RNA24**	GACCGACTCGCATTACCCTATCCTAACAGTGGAAAAGGAAGGTGG/3InvdT/
**Adapter2-BC2-Adapter1-#RNA25**	GACCGACTCGCATTACCCTATTTTCTCAGTGGAAAAGGAAGGTGG/3InvdT/
**Adapter2-BC2-Adapter1-#RNA26**	GACCGACTCGCATTACCCTATGAGAATAGTGGAAAAGGAAGGTGG/3InvdT/
**Adapter2-BC2-Adapter1-#RNA27**	GACCGACTCGCATTACCCTATATGTAAAGTGGAAAAGGAAGGTGG/3InvdT/
**Adapter2-BC2-Adapter1-#RNA28**	GACCGACTCGCATTACCCTATATTTATAGTGGAAAAGGAAGGTGG/3InvdT/
**Adapter2-BC2-Adapter1-#RNA29**	GACCGACTCGCATTACCCTATGCGATAAGTGGAAAAGGAAGGTGG/3InvdT/
**Adapter2-BC2-Adapter1-#RNA30**	GACCGACTCGCATTACCCTATATGAACAGTGGAAAAGGAAGGTGG/3InvdT/
**Adapter2-BC2-Adapter1-#RNA31**	GACCGACTCGCATTACCCTATAATGTCAGTGGAAAAGGAAGGTGG/3InvdT/
**Adapter2-BC2-Adapter1-#RNA32**	GACCGACTCGCATTACCCTATTCGCTTAGTGGAAAAGGAAGGTGG/3InvdT/
**Adapter2-BC2-Adapter1-#RNA33**	GACCGACTCGCATTACCCTATAACGGTAGTGGAAAAGGAAGGTGG/3InvdT/
**Adapter2-BC2-Adapter1-#RNA34**	GACCGACTCGCATTACCCTATATAGACAGTGGAAAAGGAAGGTGG/3InvdT/
**Adapter2-BC2-Adapter1-#RNA35**	GACCGACTCGCATTACCCTATAATACTAGTGGAAAAGGAAGGTGG/3InvdT/
**Adapter2-BC2-Adapter1-#RNA36**	GACCGACTCGCATTACCCTATGAACCAAGTGGAAAAGGAAGGTGG/3InvdT/
**Adapter2-BC2-Adapter1-#RNA37**	GACCGACTCGCATTACCCTATGGACATAGTGGAAAAGGAAGGTGG/3InvdT/
**Adapter2-BC2-Adapter1-#RNA38**	GACCGACTCGCATTACCCTATGCAAATAGTGGAAAAGGAAGGTGG/3InvdT/
**Adapter2-BC2-Adapter1-#RNA39**	GACCGACTCGCATTACCCTATATTTGCAGTGGAAAAGGAAGGTGG/3InvdT/
**Adapter2-BC2-Adapter1-#RNA40**	GACCGACTCGCATTACCCTATCGCATTAGTGGAAAAGGAAGGTGG/3InvdT/
**Adapter2-BC2-Adapter1-#RNA41**	GACCGACTCGCATTACCCTATAGCTCAAGTGGAAAAGGAAGGTGG/3InvdT/
**Adapter2-BC2-Adapter1-#RNA42**	GACCGACTCGCATTACCCTATTAAGCTAGTGGAAAAGGAAGGTGG/3InvdT/
**Adapter2-BC2-Adapter1-#RNA43**	GACCGACTCGCATTACCCTATCGTACAAGTGGAAAAGGAAGGTGG/3InvdT/
**Adapter2-BC2-Adapter1-#RNA44**	GACCGACTCGCATTACCCTATCTGAATAGTGGAAAAGGAAGGTGG/3InvdT/
**Adapter2-BC2-Adapter1-#RNA45**	GACCGACTCGCATTACCCTATCCCAATAGTGGAAAAGGAAGGTGG/3InvdT/
**Adapter2-BC2-Adapter1-#RNA46**	GACCGACTCGCATTACCCTATCCTCAAAGTGGAAAAGGAAGGTGG/3InvdT/
**Adapter2-BC2-Adapter1-#RNA47**	GACCGACTCGCATTACCCTATGCAATCAGTGGAAAAGGAAGGTGG/3InvdT/
**Adapter2-BC2-Adapter1-#RNA48**	GACCGACTCGCATTACCCTATGAATCCAGTGGAAAAGGAAGGTGG/3InvdT/
**Adapter2-BC2-Adapter1-#RNA49**	GACCGACTCGCATTACCCTATAAGCCAAGTGGAAAAGGAAGGTGG/3InvdT/
**Adapter2-BC2-Adapter1-#RNA50**	GACCGACTCGCATTACCCTATGCATTAAGTGGAAAAGGAAGGTGG/3InvdT/
**Adapter2-BC2-Adapter1-#RNA51**	GACCGACTCGCATTACCCTATGAACTTAGTGGAAAAGGAAGGTGG/3InvdT/
**Adapter2-BC2-Adapter1-#RNA52**	GACCGACTCGCATTACCCTATACTTACAGTGGAAAAGGAAGGTGG/3InvdT/

**Adapter2-BC2-Adapter1-#RNA53**	GACCGACTCGCATTACCCTATAAGTGAAGTGGAAAAGGAAGGTGG/3InvdT/
**Adapter2-BC2-Adapter1-#RNA54**	GACCGACTCGCATTACCCTATAAACTAAGTGGAAAAGGAAGGTGG/3InvdT/
**Adapter2-BC2-Adapter1-#RNA55**	GACCGACTCGCATTACCCTATGAGTTAAGTGGAAAAGGAAGGTGG/3InvdT/
**Adapter2-BC2-Adapter1-#RNA56**	GACCGACTCGCATTACCCTATAATCAAAGTGGAAAAGGAAGGTGG/3InvdT/
**Adapter2-BC2-Adapter1-#RNA57**	GACCGACTCGCATTACCCTATCTGTGAAGTGGAAAAGGAAGGTGG/3InvdT/
**Adapter2-BC2-Adapter1-#RNA58**	GACCGACTCGCATTACCCTATAAAGTTAGTGGAAAAGGAAGGTGG/3InvdT/
**Adapter2-BC2-Adapter1-#RNA59**	GACCGACTCGCATTACCCTATAACTAAAGTGGAAAAGGAAGGTGG/3InvdT/
**Adapter2-BC2-Adapter1-#RNA60**	GACCGACTCGCATTACCCTATTTACCTAGTGGAAAAGGAAGGTGG/3InvdT/
**Adapter2-BC2-Adapter1-#RNA61**	GACCGACTCGCATTACCCTATGGATTCAGTGGAAAAGGAAGGTGG/3InvdT/
**Adapter2-BC2-Adapter1-#RNA62**	GACCGACTCGCATTACCCTATAGAATTAGTGGAAAAGGAAGGTGG/3InvdT/
**Adapter2-BC2-Adapter1-#RNA63**	GACCGACTCGCATTACCCTATTTTGGTAGTGGAAAAGGAAGGTGG/3InvdT/
**Adapter2-BC2-Adapter1-#RNA64**	GACCGACTCGCATTACCCTATAACTCCAGTGGAAAAGGAAGGTGG/3InvdT/
**Adapter2-BC2-Adapter1-#RNA65**	GACCGACTCGCATTACCCTATAACCATAGTGGAAAAGGAAGGTGG/3InvdT/
**Adapter2-BC2-Adapter1-#RNA66**	GACCGACTCGCATTACCCTATATAGTAAGTGGAAAAGGAAGGTGG/3InvdT/
**Adapter2-BC2-Adapter1-#RNA67**	GACCGACTCGCATTACCCTATAAGTTCAGTGGAAAAGGAAGGTGG/3InvdT/
**Adapter2-BC2-Adapter1-#RNA68**	GACCGACTCGCATTACCCTATGAATAAAGTGGAAAAGGAAGGTGG/3InvdT/
**Adapter2-BC2-Adapter1-#RNA69**	GACCGACTCGCATTACCCTATAATCCCAGTGGAAAAGGAAGGTGG/3InvdT/
**Adapter2-BC2-Adapter1-#RNA70**	GACCGACTCGCATTACCCTATCCATGAAGTGGAAAAGGAAGGTGG/3InvdT/
**Adapter2-BC2-Adapter1-#RNA71**	GACCGACTCGCATTACCCTATACAATAAGTGGAAAAGGAAGGTGG/3InvdT/
**Adapter2-BC2-Adapter1-#RNA72**	GACCGACTCGCATTACCCTATTCAGACAGTGGAAAAGGAAGGTGG/3InvdT/
**Adapter2-BC2-Adapter1-#RNA73**	GACCGACTCGCATTACCCTATATTTTAAGTGGAAAAGGAAGGTGG/3InvdT/
**Adapter2-BC2-Adapter1-#RNA74**	GACCGACTCGCATTACCCTATGACTTTAGTGGAAAAGGAAGGTGG/3InvdT/
**Adapter2-BC2-Adapter1-#RNA75**	GACCGACTCGCATTACCCTATATGTTTAGTGGAAAAGGAAGGTGG/3InvdT/
**Adapter2-BC2-Adapter1-#RNA76**	GACCGACTCGCATTACCCTATAGACAAAGTGGAAAAGGAAGGTGG/3InvdT/
**Adapter2-BC2-Adapter1-#RNA77**	GACCGACTCGCATTACCCTATAAACGCAGTGGAAAAGGAAGGTGG/3InvdT/
**Adapter2-BC2-Adapter1-#RNA78**	GACCGACTCGCATTACCCTATATTGAAAGTGGAAAAGGAAGGTGG/3InvdT/
**Adapter2-BC2-Adapter1-#RNA79**	GACCGACTCGCATTACCCTATTTACGCAGTGGAAAAGGAAGGTGG/3InvdT/
**Adapter2-BC2-Adapter1-#RNA80**	GACCGACTCGCATTACCCTATCCTACTAGTGGAAAAGGAAGGTGG/3InvdT/
**Adapter2-BC2-Adapter1-#RNA81**	GACCGACTCGCATTACCCTATGCAGTTAGTGGAAAAGGAAGGTGG/3InvdT/
**Adapter2-BC2-Adapter1-#RNA82**	GACCGACTCGCATTACCCTATCTTTCAAGTGGAAAAGGAAGGTGG/3InvdT/
**Adapter2-BC2-Adapter1-#RNA83**	GACCGACTCGCATTACCCTATCGTTACAGTGGAAAAGGAAGGTGG/3InvdT/
**Adapter2-BC2-Adapter1-#RNA84**	GACCGACTCGCATTACCCTATAGTTTCAGTGGAAAAGGAAGGTGG/3InvdT/
**Adapter2-BC2-Adapter1-#RNA85**	GACCGACTCGCATTACCCTATAGAAACAGTGGAAAAGGAAGGTGG/3InvdT/
**Adapter2-BC2-Adapter1-#RNA86**	GACCGACTCGCATTACCCTATAAATATAGTGGAAAAGGAAGGTGG/3InvdT/
**Adapter2-BC2-Adapter1-#RNA87**	GACCGACTCGCATTACCCTATTATCGCAGTGGAAAAGGAAGGTGG/3InvdT/
**Adapter2-BC2-Adapter1-#RNA88**	GACCGACTCGCATTACCCTATCGATTAAGTGGAAAAGGAAGGTGG/3InvdT/
**Adapter2-BC2-Adapter1-#RNA89**	GACCGACTCGCATTACCCTATAGGACTAGTGGAAAAGGAAGGTGG/3InvdT/
**Adapter2-BC2-Adapter1-#RNA90**	GACCGACTCGCATTACCCTATCCAAGTAGTGGAAAAGGAAGGTGG/3InvdT/
**Adapter2-BC2-Adapter1-#RNA91**	GACCGACTCGCATTACCCTATATGGGTAGTGGAAAAGGAAGGTGG/3InvdT/
**Adapter2-BC2-Adapter1-#RNA92**	GACCGACTCGCATTACCCTATTTTAATAGTGGAAAAGGAAGGTGG/3InvdT/
**Adapter2-BC2-Adapter1-#RNA93**	GACCGACTCGCATTACCCTATAACGTAAGTGGAAAAGGAAGGTGG/3InvdT/
**Adapter2-BC2-Adapter1-#RNA94**	GACCGACTCGCATTACCCTATTCAGGTAGTGGAAAAGGAAGGTGG/3InvdT/
**Adapter2-BC2-Adapter1-#RNA95**	GACCGACTCGCATTACCCTATAATTCAAGTGGAAAAGGAAGGTGG/3InvdT/
**Adapter2-BC2-Adapter1-#RNA96**	GACCGACTCGCATTACCCTATACATGCAGTGGAAAAGGAAGGTGG/3InvdT/

Third Split-Pool Barcodes

Oligo name	Sequence
Adapter3-BC3-Adapter2-#RNA1	AAGGCTCTTGACACGAAGGATAG TACCGTNNGACCGACTCGCATTACCCT*A*T
Adapter3-BC3-Adapter2-#RNA2	AAGGCTCTTGACACGAAGGATAG CCATTTNNGACCGACTCGCATTACCCT*A*T
Adapter3-BC3-Adapter2-#RNA3	AAGGCTCTTGACACGAAGGATAG TCTTTTNNGACCGACTCGCATTACCCT*A*T
Adapter3-BC3-Adapter2-#RNA4	AAGGCTCTTGACACGAAGGATAG AAACATNNGACCGACTCGCATTACCCT*A*T
Adapter3-BC3-Adapter2-#RNA5	AAGGCTCTTGACACGAAGGATAG GTTTGGNNGACCGACTCGCATTACCCT*A*T
Adapter3-BC3-Adapter2-#RNA6	AAGGCTCTTGACACGAAGGATAG CAAACTNNGACCGACTCGCATTACCCT*A*T
Adapter3-BC3-Adapter2-#RNA7	AAGGCTCTTGACACGAAGGATAG ACCTATNNGACCGACTCGCATTACCCT*A*T
Adapter3-BC3-Adapter2-#RNA8	AAGGCTCTTGACACGAAGGATAG TCACTTNNGACCGACTCGCATTACCCT*A*T
Adapter3-BC3-Adapter2-#RNA9	AAGGCTCTTGACACGAAGGATAG AGTTGTNNGACCGACTCGCATTACCCT*A*T
Adapter3-BC3-Adapter2-#RNA10	AAGGCTCTTGACACGAAGGATAG CTTGGANNGACCGACTCGCATTACCCT*A*T
Adapter3-BC3-Adapter2-#RNA11	AAGGCTCTTGACACGAAGGATAG CTGGATNNGACCGACTCGCATTACCCT*A*T
Adapter3-BC3-Adapter2-#RNA12	AAGGCTCTTGACACGAAGGATAG TTCACGNNGACCGACTCGCATTACCCT*A*T
Adapter3-BC3-Adapter2-#RNA13	AAGGCTCTTGACACGAAGGATAG CTCTATNNGACCGACTCGCATTACCCT*A*T
Adapter3-BC3-Adapter2-#RNA14	AAGGCTCTTGACACGAAGGATAG AGAATGNNGACCGACTCGCATTACCCT*A*T
Adapter3-BC3-Adapter2-#RNA15	AAGGCTCTTGACACGAAGGATAG CATCCANNGACCGACTCGCATTACCCT*A*T
Adapter3-BC3-Adapter2-#RNA16	AAGGCTCTTGACACGAAGGATAG GTTTCTNNGACCGACTCGCATTACCCT*A*T
Adapter3-BC3-Adapter2-#RNA17	AAGGCTCTTGACACGAAGGATAG TCAGATNNGACCGACTCGCATTACCCT*A*T
Adapter3-BC3-Adapter2-#RNA18	AAGGCTCTTGACACGAAGGATAG CAATGGNNGACCGACTCGCATTACCCT*A*T
Adapter3-BC3-Adapter2-#RNA19	AAGGCTCTTGACACGAAGGATAG TGCTCANNGACCGACTCGCATTACCCT*A*T
Adapter3-BC3-Adapter2-#RNA20	AAGGCTCTTGACACGAAGGATAG TCTGGANNGACCGACTCGCATTACCCT*A*T
Adapter3-BC3-Adapter2-#RNA21	AAGGCTCTTGACACGAAGGATAG CTTTTGNNGACCGACTCGCATTACCCT*A*T
Adapter3-BC3-Adapter2-#RNA22	AAGGCTCTTGACACGAAGGATAG ACTCAGNNGACCGACTCGCATTACCCT*A*T
Adapter3-BC3-Adapter2-#RNA23	AAGGCTCTTGACACGAAGGATAG AACTAGNNGACCGACTCGCATTACCCT*A*T
Adapter3-BC3-Adapter2-#RNA24	AAGGCTCTTGACACGAAGGATAG CCCTTANNGACCGACTCGCATTACCCT*A*T
Adapter3-BC3-Adapter2-#RNA25	AAGGCTCTTGACACGAAGGATAG CTGATGNNGACCGACTCGCATTACCCT*A*T
Adapter3-BC3-Adapter2-#RNA26	AAGGCTCTTGACACGAAGGATAG CATCAGNNGACCGACTCGCATTACCCT*A*T
Adapter3-BC3-Adapter2-#RNA27	AAGGCTCTTGACACGAAGGATAG AACCTGNNGACCGACTCGCATTACCCT*A*T
Adapter3-BC3-Adapter2-#RNA28	AAGGCTCTTGACACGAAGGATAG CAAATGNNGACCGACTCGCATTACCCT*A*T
Adapter3-BC3-Adapter2-#RNA29	AAGGCTCTTGACACGAAGGATAG TCCCTANNGACCGACTCGCATTACCCT*A*T
Adapter3-BC3-Adapter2-#RNA30	AAGGCTCTTGACACGAAGGATAG TTTATGNNGACCGACTCGCATTACCCT*A*T
Adapter3-BC3-Adapter2-#RNA31	AAGGCTCTTGACACGAAGGATAG CTAAGANNGACCGACTCGCATTACCCT*A*T
Adapter3-BC3-Adapter2-#RNA32	AAGGCTCTTGACACGAAGGATAG AATACANNGACCGACTCGCATTACCCT*A*T
Adapter3-BC3-Adapter2-#RNA33	AAGGCTCTTGACACGAAGGATAG CTAACGNNGACCGACTCGCATTACCCT*A*T
Adapter3-BC3-Adapter2-#RNA34	AAGGCTCTTGACACGAAGGATAG TTCTTANNGACCGACTCGCATTACCCT*A*T
Adapter3-BC3-Adapter2-#RNA35	AAGGCTCTTGACACGAAGGATAG GATGGANNGACCGACTCGCATTACCCT*A*T
Adapter3-BC3-Adapter2-#RNA36	AAGGCTCTTGACACGAAGGATAG GGGATTNNGACCGACTCGCATTACCCT*A*T
Adapter3-BC3-Adapter2-#RNA37	AAGGCTCTTGACACGAAGGATAG GATATTNNGACCGACTCGCATTACCCT*A*T
Adapter3-BC3-Adapter2-#RNA38	AAGGCTCTTGACACGAAGGATAG AACTTTNNGACCGACTCGCATTACCCT*A*T
Adapter3-BC3-Adapter2-#RNA39	AAGGCTCTTGACACGAAGGATAG GCAACANNGACCGACTCGCATTACCCT*A*T
Adapter3-BC3-Adapter2-#RNA40	AAGGCTCTTGACACGAAGGATAG AGAGAGNNGACCGACTCGCATTACCCT*A*T
Adapter3-BC3-Adapter2-#RNA41	AAGGCTCTTGACACGAAGGATAG TTTGGGNNGACCGACTCGCATTACCCT*A*T
Adapter3-BC3-Adapter2-#RNA42	AAGGCTCTTGACACGAAGGATAG GAGAGANNGACCGACTCGCATTACCCT*A*T
Adapter3-BC3-Adapter2-#RNA43	AAGGCTCTTGACACGAAGGATAG TCCTCTNNGACCGACTCGCATTACCCT*A*T
Adapter3-BC3-Adapter2-#RNA44	AAGGCTCTTGACACGAAGGATAG CCTCTANNGACCGACTCGCATTACCCT*A*T
Adapter3-BC3-Adapter2-#RNA45	AAGGCTCTTGACACGAAGGATAG CTTAGGNNGACCGACTCGCATTACCCT*A*T
Adapter3-BC3-Adapter2-#RNA46	AAGGCTCTTGACACGAAGGATAG TTTTATNNGACCGACTCGCATTACCCT*A*T
Adapter3-BC3-Adapter2-#RNA47	AAGGCTCTTGACACGAAGGATAG TAGCTTNNGACCGACTCGCATTACCCT*A*T
Adapter3-BC3-Adapter2-#RNA48	AAGGCTCTTGACACGAAGGATAG TCAACTNNGACCGACTCGCATTACCCT*A*T
Adapter3-BC3-Adapter2-#RNA49	AAGGCTCTTGACACGAAGGATAG AGTATTNNGACCGACTCGCATTACCCT*A*T
Adapter3-BC3-Adapter2-#RNA50	AAGGCTCTTGACACGAAGGATAG TGCTATNNGACCGACTCGCATTACCCT*A*T
Adapter3-BC3-Adapter2-#RNA51	AAGGCTCTTGACACGAAGGATAG GTTCCANNGACCGACTCGCATTACCCT*A*T
Adapter3-BC3-Adapter2-#RNA52	AAGGCTCTTGACACGAAGGATAG TGAACGNNGACCGACTCGCATTACCCT*A*T

**Adapter3-BC3-Adapter2-#RNA53**	AAGGCTCTTGACACGAAGGATAG TGATGGNNGACCGACTCGCATTACCCT*A*T
**Adapter3-BC3-Adapter2-#RNA54**	AAGGCTCTTGACACGAAGGATAG GTATTTNNGACCGACTCGCATTACCCT*A*T
**Adapter3-BC3-Adapter2-#RNA55**	AAGGCTCTTGACACGAAGGATAG GTACAGNNGACCGACTCGCATTACCCT*A*T
**Adapter3-BC3-Adapter2-#RNA56**	AAGGCTCTTGACACGAAGGATAG AAACCGNNGACCGACTCGCATTACCCT*A*T
**Adapter3-BC3-Adapter2-#RNA57**	AAGGCTCTTGACACGAAGGATAG CATTTANNGACCGACTCGCATTACCCT*A*T
**Adapter3-BC3-Adapter2-#RNA58**	AAGGCTCTTGACACGAAGGATAG AGGGATNNGACCGACTCGCATTACCCT*A*T
**Adapter3-BC3-Adapter2-#RNA59**	AAGGCTCTTGACACGAAGGATAG AAATCANNGACCGACTCGCATTACCCT*A*T
**Adapter3-BC3-Adapter2-#RNA60**	AAGGCTCTTGACACGAAGGATAG GGAACTNNGACCGACTCGCATTACCCT*A*T
**Adapter3-BC3-Adapter2-#RNA61**	AAGGCTCTTGACACGAAGGATAG CTCATANNGACCGACTCGCATTACCCT*A*T
**Adapter3-BC3-Adapter2-#RNA62**	AAGGCTCTTGACACGAAGGATAG TAATCGNNGACCGACTCGCATTACCCT*A*T
**Adapter3-BC3-Adapter2-#RNA63**	AAGGCTCTTGACACGAAGGATAG TAGTTANNGACCGACTCGCATTACCCT*A*T
**Adapter3-BC3-Adapter2-#RNA64**	AAGGCTCTTGACACGAAGGATAG AATATGNNGACCGACTCGCATTACCCT*A*T
**Adapter3-BC3-Adapter2-#RNA65**	AAGGCTCTTGACACGAAGGATAG TAGTCTNNGACCGACTCGCATTACCCT*A*T
**Adapter3-BC3-Adapter2-#RNA66**	AAGGCTCTTGACACGAAGGATAG CATTCTNNGACCGACTCGCATTACCCT*A*T
**Adapter3-BC3-Adapter2-#RNA67**	AAGGCTCTTGACACGAAGGATAG TTATGANNGACCGACTCGCATTACCCT*A*T
**Adapter3-BC3-Adapter2-#RNA68**	AAGGCTCTTGACACGAAGGATAG CCTCATNNGACCGACTCGCATTACCCT*A*T
**Adapter3-BC3-Adapter2-#RNA69**	AAGGCTCTTGACACGAAGGATAG GACTTANNGACCGACTCGCATTACCCT*A*T
**Adapter3-BC3-Adapter2-#RNA70**	AAGGCTCTTGACACGAAGGATAG ACAACGNNGACCGACTCGCATTACCCT*A*T
**Adapter3-BC3-Adapter2-#RNA71**	AAGGCTCTTGACACGAAGGATAG GATTTGNNGACCGACTCGCATTACCCT*A*T
**Adapter3-BC3-Adapter2-#RNA72**	AAGGCTCTTGACACGAAGGATAG GACCTTNNGACCGACTCGCATTACCCT*A*T
**Adapter3-BC3-Adapter2-#RNA73**	AAGGCTCTTGACACGAAGGATAG CGAATTNNGACCGACTCGCATTACCCT*A*T
**Adapter3-BC3-Adapter2-#RNA74**	AAGGCTCTTGACACGAAGGATAG TTATTGNNGACCGACTCGCATTACCCT*A*T
**Adapter3-BC3-Adapter2-#RNA75**	AAGGCTCTTGACACGAAGGATAG ACTTTGNNGACCGACTCGCATTACCCT*A*T
**Adapter3-BC3-Adapter2-#RNA76**	AAGGCTCTTGACACGAAGGATAG AACTGANNGACCGACTCGCATTACCCT*A*T
**Adapter3-BC3-Adapter2-#RNA77**	AAGGCTCTTGACACGAAGGATAG GCTACTNNGACCGACTCGCATTACCCT*A*T
**Adapter3-BC3-Adapter2-#RNA78**	AAGGCTCTTGACACGAAGGATAG AAATTGNNGACCGACTCGCATTACCCT*A*T
**Adapter3-BC3-Adapter2-#RNA79**	AAGGCTCTTGACACGAAGGATAG ACCTCANNGACCGACTCGCATTACCCT*A*T
**Adapter3-BC3-Adapter2-#RNA80**	AAGGCTCTTGACACGAAGGATAG GGATGANNGACCGACTCGCATTACCCT*A*T
**Adapter3-BC3-Adapter2-#RNA81**	AAGGCTCTTGACACGAAGGATAG GACATGNNGACCGACTCGCATTACCCT*A*T
**Adapter3-BC3-Adapter2-#RNA82**	AAGGCTCTTGACACGAAGGATAG AATCTTNNGACCGACTCGCATTACCCT*A*T
**Adapter3-BC3-Adapter2-#RNA83**	AAGGCTCTTGACACGAAGGATAG TAATTTNNGACCGACTCGCATTACCCT*A*T
**Adapter3-BC3-Adapter2-#RNA84**	AAGGCTCTTGACACGAAGGATAG TCCTGANNGACCGACTCGCATTACCCT*A*T
**Adapter3-BC3-Adapter2-#RNA85**	AAGGCTCTTGACACGAAGGATAG GTTCTTNNGACCGACTCGCATTACCCT*A*T
**Adapter3-BC3-Adapter2-#RNA86**	AAGGCTCTTGACACGAAGGATAG GCTTCANNGACCGACTCGCATTACCCT*A*T
**Adapter3-BC3-Adapter2-#RNA87**	AAGGCTCTTGACACGAAGGATAG TTAGAGNNGACCGACTCGCATTACCCT*A*T
**Adapter3-BC3-Adapter2-#RNA88**	AAGGCTCTTGACACGAAGGATAG TAACTGNNGACCGACTCGCATTACCCT*A*T
**Adapter3-BC3-Adapter2-#RNA89**	AAGGCTCTTGACACGAAGGATAG CTGTTANNGACCGACTCGCATTACCCT*A*T
**Adapter3-BC3-Adapter2-#RNA90**	AAGGCTCTTGACACGAAGGATAG CACCATNNGACCGACTCGCATTACCCT*A*T
**Adapter3-BC3-Adapter2-#RNA91**	AAGGCTCTTGACACGAAGGATAG AAATGTNNGACCGACTCGCATTACCCT*A*T
**Adapter3-BC3-Adapter2-#RNA92**	AAGGCTCTTGACACGAAGGATAG CTCTCANNGACCGACTCGCATTACCCT*A*T
**Adapter3-BC3-Adapter2-#RNA93**	AAGGCTCTTGACACGAAGGATAG TTCCCTNNGACCGACTCGCATTACCCT*A*T
**Adapter3-BC3-Adapter2-#RNA94**	AAGGCTCTTGACACGAAGGATAG TTCGATNNGACCGACTCGCATTACCCT*A*T
**Adapter3-BC3-Adapter2-#RNA95**	AAGGCTCTTGACACGAAGGATAG CTATGTNNGACCGACTCGCATTACCCT*A*T
**Adapter3-BC3-Adapter2-#RNA96**	AAGGCTCTTGACACGAAGGATAG TCATCANNGACCGACTCGCATTACCCT*A*T

Second Split-Pool Barcodes for 2 cycles Split-Pool

Oligo name	Sequence
Adapter3-BC2a-Adapter1-#RNA1	AAGGCTCTTGACACGAAGGATAG CACAGAAGTGGAAAAGGAAGGTG*G*T
Adapter3-BC2a-Adapter1-#RNA2	AAGGCTCTTGACACGAAGGATAG TAGTAAAGTGGAAAAGGAAGGTG*G*T
Adapter3-BC2a-Adapter1-#RNA3	AAGGCTCTTGACACGAAGGATAG TACGCTAGTGGAAAAGGAAGGTG*G*T
Adapter3-BC2a-Adapter1-#RNA4	AAGGCTCTTGACACGAAGGATAG TTTAGCAGTGGAAAAGGAAGGTG*G*T
Adapter3-BC2a-Adapter1-#RNA5	AAGGCTCTTGACACGAAGGATAG GGAAGAAGTGGAAAAGGAAGGTG*G*T
Adapter3-BC2a-Adapter1-#RNA6	AAGGCTCTTGACACGAAGGATAG TACCTGAGTGGAAAAGGAAGGTG*G*T
Adapter3-BC2a-Adapter1-#RNA7	AAGGCTCTTGACACGAAGGATAG AAAGTCAGTGGAAAAGGAAGGTG*G*T
Adapter3-BC2a-Adapter1-#RNA8	AAGGCTCTTGACACGAAGGATAG GGTACAAGTGGAAAAGGAAGGTG*G*T
Adapter3-BC2a-Adapter1-#RNA9	AAGGCTCTTGACACGAAGGATAG AATGAGAGTGGAAAAGGAAGGTG*G*T
Adapter3-BC2a-Adapter1-#RNA10	AAGGCTCTTGACACGAAGGATAG GGATTCAGTGGAAAAGGAAGGTG*G*T
Adapter3-BC2a-Adapter1-#RNA11	AAGGCTCTTGACACGAAGGATAG TTAGCCAGTGGAAAAGGAAGGTG*G*T
Adapter3-BC2a-Adapter1-#RNA12	AAGGCTCTTGACACGAAGGATAG TCATCGAGTGGAAAAGGAAGGTG*G*T
Adapter3-BC2a-Adapter1-#RNA13	AAGGCTCTTGACACGAAGGATAG CAGGTTAGTGGAAAAGGAAGGTG*G*T
Adapter3-BC2a-Adapter1-#RNA14	AAGGCTCTTGACACGAAGGATAG AACGGAAGTGGAAAAGGAAGGTG*G*T
Adapter3-BC2a-Adapter1-#RNA15	AAGGCTCTTGACACGAAGGATAG ACTGTGAGTGGAAAAGGAAGGTG*G*T
Adapter3-BC2a-Adapter1-#RNA16	AAGGCTCTTGACACGAAGGATAG CCAATAAGTGGAAAAGGAAGGTG*G*T
Adapter3-BC2a-Adapter1-#RNA17	AAGGCTCTTGACACGAAGGATAG TTCAGGAGTGGAAAAGGAAGGTG*G*T
Adapter3-BC2a-Adapter1-#RNA18	AAGGCTCTTGACACGAAGGATAG CTTAATAGTGGAAAAGGAAGGTG*G*T
Adapter3-BC2a-Adapter1-#RNA19	AAGGCTCTTGACACGAAGGATAG GTTCAAAGTGGAAAAGGAAGGTG*G*T
Adapter3-BC2a-Adapter1-#RNA20	AAGGCTCTTGACACGAAGGATAG TTTGTAAGTGGAAAAGGAAGGTG*G*T
Adapter3-BC2a-Adapter1-#RNA21	AAGGCTCTTGACACGAAGGATAG CTAAAAAGTGGAAAAGGAAGGTG*G*T
Adapter3-BC2a-Adapter1-#RNA22	AAGGCTCTTGACACGAAGGATAG TGGAATAGTGGAAAAGGAAGGTG*G*T
Adapter3-BC2a-Adapter1-#RNA23	AAGGCTCTTGACACGAAGGATAG GAAACAAGTGGAAAAGGAAGGTG*G*T
Adapter3-BC2a-Adapter1-#RNA24	AAGGCTCTTGACACGAAGGATAG AGTTTTAGTGGAAAAGGAAGGTG*G*T
Adapter3-BC2a-Adapter1-#RNA25	AAGGCTCTTGACACGAAGGATAG GCGAAAAGTGGAAAAGGAAGGTG*G*T
Adapter3-BC2a-Adapter1-#RNA26	AAGGCTCTTGACACGAAGGATAG TCTAAAAGTGGAAAAGGAAGGTG*G*T
Adapter3-BC2a-Adapter1-#RNA27	AAGGCTCTTGACACGAAGGATAG CGAGTTAGTGGAAAAGGAAGGTG*G*T
Adapter3-BC2a-Adapter1-#RNA28	AAGGCTCTTGACACGAAGGATAG AAATGGAGTGGAAAAGGAAGGTG*G*T
Adapter3-BC2a-Adapter1-#RNA29	AAGGCTCTTGACACGAAGGATAG CTAATGAGTGGAAAAGGAAGGTG*G*T
Adapter3-BC2a-Adapter1-#RNA30	AAGGCTCTTGACACGAAGGATAG CTAAGTAGTGGAAAAGGAAGGTG*G*T
Adapter3-BC2a-Adapter1-#RNA31	AAGGCTCTTGACACGAAGGATAG TTTGAGAGTGGAAAAGGAAGGTG*G*T
Adapter3-BC2a-Adapter1-#RNA32	AAGGCTCTTGACACGAAGGATAG TCTGGAAGTGGAAAAGGAAGGTG*G*T
Adapter3-BC2a-Adapter1-#RNA33	AAGGCTCTTGACACGAAGGATAG GGATAAAGTGGAAAAGGAAGGTG*G*T
Adapter3-BC2a-Adapter1-#RNA34	AAGGCTCTTGACACGAAGGATAG TAGGATAGTGGAAAAGGAAGGTG*G*T
Adapter3-BC2a-Adapter1-#RNA35	AAGGCTCTTGACACGAAGGATAG TAAGTGAGTGGAAAAGGAAGGTG*G*T
Adapter3-BC2a-Adapter1-#RNA36	AAGGCTCTTGACACGAAGGATAG CTTCACAGTGGAAAAGGAAGGTG*G*T
Adapter3-BC2a-Adapter1-#RNA37	AAGGCTCTTGACACGAAGGATAG GTCATAAGTGGAAAAGGAAGGTG*G*T
Adapter3-BC2a-Adapter1-#RNA38	AAGGCTCTTGACACGAAGGATAG AGGTGAAGTGGAAAAGGAAGGTG*G*T
Adapter3-BC2a-Adapter1-#RNA39	AAGGCTCTTGACACGAAGGATAG ACGATCAGTGGAAAAGGAAGGTG*G*T
Adapter3-BC2a-Adapter1-#RNA40	AAGGCTCTTGACACGAAGGATAG GAGTTTAGTGGAAAAGGAAGGTG*G*T
Adapter3-BC2a-Adapter1-#RNA41	AAGGCTCTTGACACGAAGGATAG GTATTGAGTGGAAAAGGAAGGTG*G*T
Adapter3-BC2a-Adapter1-#RNA42	AAGGCTCTTGACACGAAGGATAG GCAATCAGTGGAAAAGGAAGGTG*G*T
Adapter3-BC2a-Adapter1-#RNA43	AAGGCTCTTGACACGAAGGATAG GATATTAGTGGAAAAGGAAGGTG*G*T
Adapter3-BC2a-Adapter1-#RNA44	AAGGCTCTTGACACGAAGGATAG TTACAGAGTGGAAAAGGAAGGTG*G*T
Adapter3-BC2a-Adapter1-#RNA45	AAGGCTCTTGACACGAAGGATAG TCGTTAAGTGGAAAAGGAAGGTG*G*T
Adapter3-BC2a-Adapter1-#RNA46	AAGGCTCTTGACACGAAGGATAG TAAGAAAGTGGAAAAGGAAGGTG*G*T
Adapter3-BC2a-Adapter1-#RNA47	AAGGCTCTTGACACGAAGGATAG TCCTAAAGTGGAAAAGGAAGGTG*G*T
Adapter3-BC2a-Adapter1-#RNA48	AAGGCTCTTGACACGAAGGATAG AAACTTAGTGGAAAAGGAAGGTG*G*T
Adapter3-BC2a-Adapter1-#RNA49	AAGGCTCTTGACACGAAGGATAG CAGTGAAGTGGAAAAGGAAGGTG*G*T

**Adapter3-BC2a-Adapter1-#RNA50**	AAGGCTCTTGACACGAAGGATAG CCAGAAAGTGGAAAAGGAAGGTG*G*T
**Adapter3-BC2a-Adapter1-#RNA51**	AAGGCTCTTGACACGAAGGATAG ACAACAAGTGGAAAAGGAAGGTG*G*T
**Adapter3-BC2a-Adapter1-#RNA52**	AAGGCTCTTGACACGAAGGATAG CAGGAAAGTGGAAAAGGAAGGTG*G*T
**Adapter3-BC2a-Adapter1-#RNA53**	AAGGCTCTTGACACGAAGGATAG TGAGTAAGTGGAAAAGGAAGGTG*G*T
**Adapter3-BC2a-Adapter1-#RNA54**	AAGGCTCTTGACACGAAGGATAG GATTTAAGTGGAAAAGGAAGGTG*G*T
**Adapter3-BC2a-Adapter1-#RNA55**	AAGGCTCTTGACACGAAGGATAG AGAGAAAGTGGAAAAGGAAGGTG*G*T
**Adapter3-BC2a-Adapter1-#RNA56**	AAGGCTCTTGACACGAAGGATAG CTGAACAGTGGAAAAGGAAGGTG*G*T
**Adapter3-BC2a-Adapter1-#RNA57**	AAGGCTCTTGACACGAAGGATAG AAGCATAGTGGAAAAGGAAGGTG*G*T
**Adapter3-BC2a-Adapter1-#RNA58**	AAGGCTCTTGACACGAAGGATAG TTGTGAAGTGGAAAAGGAAGGTG*G*T
**Adapter3-BC2a-Adapter1-#RNA59**	AAGGCTCTTGACACGAAGGATAG AAACGAAGTGGAAAAGGAAGGTG*G*T
**Adapter3-BC2a-Adapter1-#RNA60**	AAGGCTCTTGACACGAAGGATAG TCCCTAAGTGGAAAAGGAAGGTG*G*T
**Adapter3-BC2a-Adapter1-#RNA61**	AAGGCTCTTGACACGAAGGATAG GAATATAGTGGAAAAGGAAGGTG*G*T
**Adapter3-BC2a-Adapter1-#RNA62**	AAGGCTCTTGACACGAAGGATAG TTGTTCAGTGGAAAAGGAAGGTG*G*T
**Adapter3-BC2a-Adapter1-#RNA63**	AAGGCTCTTGACACGAAGGATAG AACGTGAGTGGAAAAGGAAGGTG*G*T
**Adapter3-BC2a-Adapter1-#RNA64**	AAGGCTCTTGACACGAAGGATAG CATGTAAGTGGAAAAGGAAGGTG*G*T
**Adapter3-BC2a-Adapter1-#RNA65**	AAGGCTCTTGACACGAAGGATAG CATAGCAGTGGAAAAGGAAGGTG*G*T
**Adapter3-BC2a-Adapter1-#RNA66**	AAGGCTCTTGACACGAAGGATAG CAGTTCAGTGGAAAAGGAAGGTG*G*T
**Adapter3-BC2a-Adapter1-#RNA67**	AAGGCTCTTGACACGAAGGATAG TAAAACAGTGGAAAAGGAAGGTG*G*T
**Adapter3-BC2a-Adapter1-#RNA68**	AAGGCTCTTGACACGAAGGATAG AGGATTAGTGGAAAAGGAAGGTG*G*T
**Adapter3-BC2a-Adapter1-#RNA69**	AAGGCTCTTGACACGAAGGATAG GATGAAAGTGGAAAAGGAAGGTG*G*T
**Adapter3-BC2a-Adapter1-#RNA70**	AAGGCTCTTGACACGAAGGATAG CTGCAAAGTGGAAAAGGAAGGTG*G*T
**Adapter3-BC2a-Adapter1-#RNA71**	AAGGCTCTTGACACGAAGGATAG GAAGTAAGTGGAAAAGGAAGGTG*G*T
**Adapter3-BC2a-Adapter1-#RNA72**	AAGGCTCTTGACACGAAGGATAG TCAGGTAGTGGAAAAGGAAGGTG*G*T
**Adapter3-BC2a-Adapter1-#RNA73**	AAGGCTCTTGACACGAAGGATAG GAGCAAAGTGGAAAAGGAAGGTG*G*T
**Adapter3-BC2a-Adapter1-#RNA74**	AAGGCTCTTGACACGAAGGATAG CTCACAAGTGGAAAAGGAAGGTG*G*T
**Adapter3-BC2a-Adapter1-#RNA75**	AAGGCTCTTGACACGAAGGATAG ACGGTTAGTGGAAAAGGAAGGTG*G*T
**Adapter3-BC2a-Adapter1-#RNA76**	AAGGCTCTTGACACGAAGGATAG CATAAAAGTGGAAAAGGAAGGTG*G*T
**Adapter3-BC2a-Adapter1-#RNA77**	AAGGCTCTTGACACGAAGGATAG AAGCTAAGTGGAAAAGGAAGGTG*G*T
**Adapter3-BC2a-Adapter1-#RNA78**	AAGGCTCTTGACACGAAGGATAG GAAGGTAGTGGAAAAGGAAGGTG*G*T
**Adapter3-BC2a-Adapter1-#RNA79**	AAGGCTCTTGACACGAAGGATAG CATTTGAGTGGAAAAGGAAGGTG*G*T
**Adapter3-BC2a-Adapter1-#RNA80**	AAGGCTCTTGACACGAAGGATAG CAATAAAGTGGAAAAGGAAGGTG*G*T
**Adapter3-BC2a-Adapter1-#RNA81**	AAGGCTCTTGACACGAAGGATAG TAATTCAGTGGAAAAGGAAGGTG*G*T
**Adapter3-BC2a-Adapter1-#RNA82**	AAGGCTCTTGACACGAAGGATAG TCCATGAGTGGAAAAGGAAGGTG*G*T
**Adapter3-BC2a-Adapter1-#RNA83**	AAGGCTCTTGACACGAAGGATAG TGCTATAGTGGAAAAGGAAGGTG*G*T
**Adapter3-BC2a-Adapter1-#RNA84**	AAGGCTCTTGACACGAAGGATAG AGGCAAAGTGGAAAAGGAAGGTG*G*T
**Adapter3-BC2a-Adapter1-#RNA85**	AAGGCTCTTGACACGAAGGATAG AACAGGAGTGGAAAAGGAAGGTG*G*T
**Adapter3-BC2a-Adapter1-#RNA86**	AAGGCTCTTGACACGAAGGATAG CAACATAGTGGAAAAGGAAGGTG*G*T
**Adapter3-BC2a-Adapter1-#RNA87**	AAGGCTCTTGACACGAAGGATAG TCTCTCAGTGGAAAAGGAAGGTG*G*T
**Adapter3-BC2a-Adapter1-#RNA88**	AAGGCTCTTGACACGAAGGATAG AGTTCCAGTGGAAAAGGAAGGTG*G*T
**Adapter3-BC2a-Adapter1-#RNA89**	AAGGCTCTTGACACGAAGGATAG TCCAACAGTGGAAAAGGAAGGTG*G*T
**Adapter3-BC2a-Adapter1-#RNA90**	AAGGCTCTTGACACGAAGGATAG AGGAACAGTGGAAAAGGAAGGTG*G*T
**Adapter3-BC2a-Adapter1-#RNA91**	AAGGCTCTTGACACGAAGGATAG GCTTTCAGTGGAAAAGGAAGGTG*G*T
**Adapter3-BC2a-Adapter1-#RNA92**	AAGGCTCTTGACACGAAGGATAG AATGGCAGTGGAAAAGGAAGGTG*G*T
**Adapter3-BC2a-Adapter1-#RNA93**	AAGGCTCTTGACACGAAGGATAG ACACTAAGTGGAAAAGGAAGGTG*G*T
**Adapter3-BC2a-Adapter1-#RNA94**	AAGGCTCTTGACACGAAGGATAG TTGCTGAGTGGAAAAGGAAGGTG*G*T
**Adapter3-BC2a-Adapter1-#RNA95**	AAGGCTCTTGACACGAAGGATAG GGTTCTAGTGGAAAAGGAAGGTG*G*T
**Adapter3-BC2a-Adapter1-#RNA96**	AAGGCTCTTGACACGAAGGATAG CAATTTAGTGGAAAAGGAAGGTG*G*T
